# Common peptides shed light on evolution of Olfactory Receptors

**DOI:** 10.1186/1471-2148-9-91

**Published:** 2009-05-05

**Authors:** Assaf Gottlieb, Tsviya Olender, Doron Lancet, David Horn

**Affiliations:** 1 School of Physics and Astronomy, Tel Aviv University, Tel Aviv 69978, Israel; 2 Dept. of Molecular Genetics, Weizmann Institute of Science, Rehovot 76100, Israel

## Abstract

**Background:**

Olfactory Receptors (ORs) form the largest multigene family in vertebrates. Their evolution and their expansion in the vertebrate genomes was the subject of many studies. In this paper we apply a motif-based approach to this problem in order to uncover evolutionary characteristics.

**Results:**

We extract deterministic motifs from ORs belonging to ten species using the MEX (Motif Extraction) algorithm, thus defining Common Peptides (CPs) characteristic to ORs. We identify species-specific CPs and show that their relative abundance is high only in fish and frog, suggesting relevance to water-soluble odorants. We estimate the origins of CPs according to the tree of life and track the gains and losses of CPs through evolution. We identify major CP gain in tetrapods and major losses in reptiles. Although the number of human ORs is less than half of the number of ORs in other mammals, the fraction of lost CPs is only 11%.

By examining the positions of CPs along the OR sequence, we find two regions that expanded only in tetrapods. Using CPs we are able to establish remote homology relations between ORs and non-OR GPCRs.

Selecting CPs according to their evolutionary age, we bicluster ORs and CPs for each species. Clean biclustering emerges when using relatively novel CPs. Evolutionary age is used to track the history of CP acquisition in the collection of mammalian OR families within HORDE (Human Olfactory Receptor Data Explorer).

**Conclusion:**

The CP method provides a novel perspective that reveals interesting traits in the evolution of olfactory receptors. It is consistent with previous knowledge, and provides finer details. Using available phylogenetic trees, evolution can be rephrased in terms of CP origins.

Supplementary information is also available at

## Background

Odor recognition in vertebrates is mediated by a large superfamily of olfactory receptor (OR) genes, G-protein coupled receptors (GPCRs) with seven trans-membrane domains [1,2]. Whole genome studies discovered hundreds of intact ORs in the vertebrate genome, ranging in size from ~100 in fishes to ~1000 in mouse [3-5] and [6].

A recent study of OR evolutionary dynamics indicated the existence of nine ancestral genes common to fish and tetrapods, of which only two are found in birds and mammals. Specifically one of these, known as Class II, has expanded enormously in mammals [6]. Several studies have applied computational sequence analysis and phylogeny methods to study the evolution of the OR repertoire in vertebrates [6,7]. One of these studies [8] used motifs to analyze human and mouse OR repertoires, focusing on classification of the motifs into classes and classification of the ORs using these motifs as features.

We adopt a different motif-based approach that extracts deterministic motifs, i.e. peptides, and explores their appearance along OR evolution. We apply the motif extraction algorithm MEX [9], the efficacy of which has been previously demonstrated in the study of enzymes [10], to 4027 OR sequences of 10 vertebrates. A short explanation of MEX is also provided in the Methods section. The union of all motifs leads to a list of 2717 MEX-derived peptides, to be referred to as Common Peptides (CPs). These motifs can be mapped onto specific locations on the seven trans-membrane domains.

Following CP occurrences on ORs of different species we can trace the development of these domains with evolution. Using the Tree of Life, we perform an ancestral reconstruction of CPs and determine their evolutionary ages.

For each species we perform biclustering of the matrix of CP occurrences on ORs. Choosing CP groups according to their evolutionary age we get different clustering patterns.

The use of CPs for studying OR sequences enables us to explore different aspects regarding OR evolution than those uncovered by phylogenetic methods. It also enables us to uncover some fine details of OR groups that were previously studied using regular-expression motifs, due to the deterministic nature of our motifs (see also [11]).

## Results

### CP mapping on the Tree of Life

We used 4027 OR sequences representing the complete intact OR repertoires in 10 vertebrates (Table 1). We extracted a list of CPs by applying MEX to OR sequences of each species individually, followed by a unification procedure to remove redundancy (see Methods for a detailed description).

**Table 1:** Distribution of 3983 OR sequences, total CPs and species-specific CPs according to species

**Species**	**Number of ORs**	**Number of observed CPs**	**Number of species-specific CPs**	**Percentage of species-specific CPs**
pufferfish	44	193	11	5.7%

zebrafish	97	352	60	17.0%

Frog	409	1179	143	12.1%

Lizard	120	945	17	1.8%

Chicken	78	644	15	2.3%

Platypus	250	1406	26	1.8%

Opossum	846	2030	48	2.4%

Dog	814	2083	40	1.9%

Mouse	978	2179	66	3.0%

Human	391	1889	8	0.4%

All CPs are tested for their occurrence on all ORs, irrespective of which species lead to their extraction. We define *species-specific CPs *as CPs observed only in one species.

On average an OR is matched by 48 CPs, covering 147 amino acids on its sequence. Some CPs partially overlap with one another. The total number of CPs found in sequences of one species (column 3 in Table 1) is highly correlated (Pearson correlation = 0.9) with the number of ORs per species (column 2 in Table 1).

The percentage of species-specific CPs is particularly high in fish and frog (although less than 6% of the pufferfish CPs are pufferfish-specific, the percentage of fish-specific, including both fish, is 18%). The percentage of species-specific CPs drops significantly to an average of 2% in other species, with human having the smallest amount of species-specific CPs. This finding might be attributed to the difference between aquatic environment, characteristic of fish and the amphibian frog *X. tropicalis *that remains aquatic also in its adult life (see [12] and [13]), and terrestrial environments characteristic of the other species: presumably CPs were lost – together with their ORs (groups δ, ε, ζ and η in [6])- in terrestrial species that have developed later.

**Figure 1:**
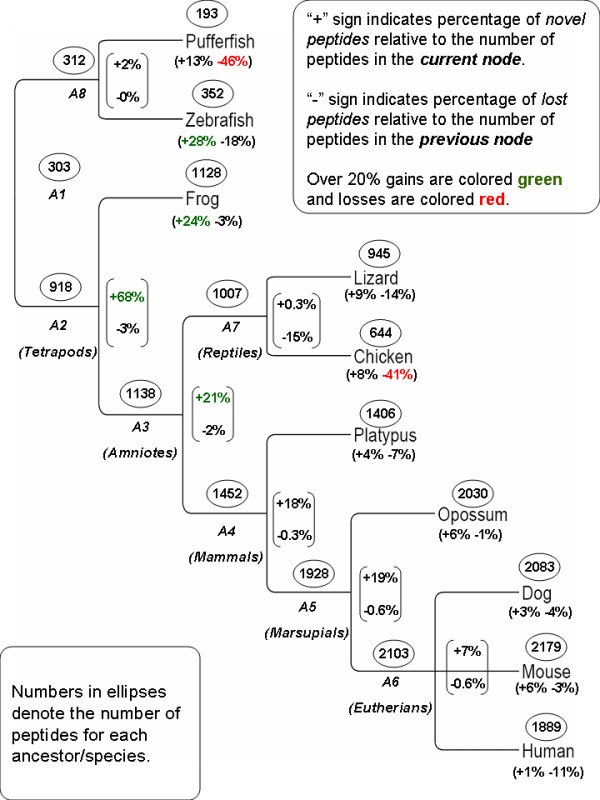
**CP reconstruction on the tree of life**. Number of CPs occurring in each species and parsimoniously estimated number of CPs occurring in each ancestor (in ellipses). Numbers in brackets indicate the percentage of *novel CPs *relative to the total number of CPs in the current node (+ sign) and the percentage of *lost CPs *relative to the total number of CPs in the previous node (- sign). Over 20% gains are colored green and lost are colored red. Ancestor names are enumerated from the most recent ancestor of fish and tetrapods (A1) to pufferfish and zebrafish ancestor (A8). As an example, zebrafish contains 97 novel CPs, which constitute 28% out of its 352 CPs. It also lost 57 CPs, which occurred in its ancestor, which constitute 18% of the CPs existing in A8.

We evaluate the emergence and loss of CPs on a commonly accepted tree of life representation (figure 1), using the parsimony method (see details on the chosen method and other tested ancestral reconstruction methods in the Methods section).

We identify "*novel CPs*" as those that exist in the current ancestor/species but did not exist in previous ancestors, and "*lost CPs*" as those that do not exist in the current ancestor/species but did exist in the previous ancestor. CPs that date back to previous ancestors are referred to as "*conserved CPs"*.

The analysis detects one major addition of novel CPs in the ancestor of tetrapods, A2. Judging by [14] the branch length between A1 and A3 is about the same as that between A3 to A6. 47% of the CPs at A6 are novel with regard to A3. This should be compared with the fact that 75% of CPs at A3 are novel with regard to A1. We thus may conclude that the main expansion of OR CPs has taken place at, or before, A3.

Reptiles have suffered major losses of CPs, a trend that was further increased in chicken. Another major loss occurred in pufferfish.

Interestingly, while humans lost more than half of their ORs relative to other mammals, they lost only 11% of the CPs existing in A6. This suggests that some redundancy in mammalian ORs has been removed by OR pseudogenization in human. This result is surprising considering the fact that the human intact OR repertoire contains much less subfamilies relative to other mammals (according to HORDE classification system [15]). For example, there are 242 and 227 subfamilies in mouse and dog respectively, but only 175 subfamilies in human. Investigating subfamilies of mouse and dog ORs that are not matched by human subfamilies, we nonetheless find many of their CPs (68% of mouse CPs and 35% of dog CPs) elsewhere in. other human subfamilies. In other words, according to the CP perspective the similarity between human and mouse or dog is larger than observed by the sequence similarity which is the basis of the subfamily classifications. [16] hypothesize that the reduced sense of smell in human could correlate with the loss of functional genes. The high co-occurrences of CPs in functional human, mouse and dog genes hints, however, that the reduction of the human OR repertoire may not necessarily cause loss of functionality.

### CPs that make a difference

The CP method extracts CPs that bear statistical significance. It is reasonable to assume that some of them also have biological significance. We first looked for CPs that differentiate between water-dwelling species (i.e. pufferfish, zebrafish and possibly frog) and purely terrestrial species. We find 10 CPs that exist in fish (one of them occurs also in frog) but not in any other land-dwelling species. Similarly, we find 44 CPs which are terrestrial specific (none of them exist in frog). Of special interest are CPs that reside in the outer region of the membrane (extracellular loops and the external half of the transmembrane domains). Such CPs might participate in ligand binding. Table 2 lists the CPs residing only in water-dwelling species. CPs that potentially play part in ligand binding are marked. Of particular interest is the CP "RLPLCG", which resides on the extracellular loop 2 and contains a Cysteine, possibly crosslinking with another Cysteine on the ORs.

**Table 2:** CPs specific to water-dwelling species. CPs facing the extracellular side of the membrane are in bold.

**CP**	**Domain**	**# of occurrences**
RYILF	TM2	15

**YGATGFYP**	**TM2**	**6**

**AGFFPR**	**TM2**	**11**

LAYDRL	IL2	9

YHSVM	IL2	10

RLPLCG *	**EL2**	**17**

KFMQTC	IL3	8

ALKTC	IL3	16

QTCVPH	IL3	16

PPILNPL	TM7	13

Domains start from the N-terminal (N), through Transmembrane domains 1–7 (T1–T7), Intracellular loops (I1–I3) and extracellular loops (E1–E3) and end in the C-terminal (C)

* – appears also in frog

Table 3 lists the CPs residing only in terrestrial species. CPs that potentially play part in ligand binding are marked. More than 2/3 of these CPs occur in ORs that belong predominantly (more than 40% of the total OR occurrences) to one HORDE family.

**Table 3:** CPs specific to land-dwelling species. CPs facing the extracellular side of the membrane are in bold.

**CP**	**Domain**	**# of occurrences**
NHTTV	N	30

QVLLF	TM1	53

TLMGN	TM1	89

GNLGM	TM1	211

LGNGTIL	TM1	20

NLGMI	TM1	181

FLSSLS	TM2	53

VDICF	TM2	71

**CFSSV**	**TM2**	**59**

**GVTEF**	**TM2**	**55**

**TVPKS**	**TM2**	**39**

**TTTVP**	**TM2**	**64**

**PKMIAD**	**TM2**	**19**

**MLVNF**	**TM2**	**153**

**LPRML**	**TM2**	**39**

**KVISF**	**EL1**	**85**

**ISFTGC**	**EL1**	**45**

**GCATQ**	**TM3**	**117**

**SYSGC**	**TM3**	**47**

**AQLFF**	**TM3**	**107**

LVAMA	TM3	122

NPLLY	IL2	349

PLHYL	IL2	110

PLLYP	TM4	68

SWLGG	TM4	54

**GLFVA**	**EL2**	**60**

**YTVIL**	**TM5**	**50**

SYGLI	TM5	34

LAVVTL	TM5	23

ILRIR	IL3	142

LRIRS	IL3	159

RKALS	IL3	161

LLFMY	TM6	61

LFFGP	TM6	133

**AYLKP**	**TM6**	**54**

**TYIRP**	**TM6**	**29**

**YLRPSS**	**TM6**	**50**

**IYARP**	**TM6**	**49**

**VALFY**	**TM6**	**50**

**RPSSS**	**TM6**	**86**

**LFYTI**	**TM7**	**115**

EVKGA	C	108

GALRR	C	65

AMRKL	C	61

Domains are the same as in table 2.

### GPCR remote homologies

**Figure 2:**
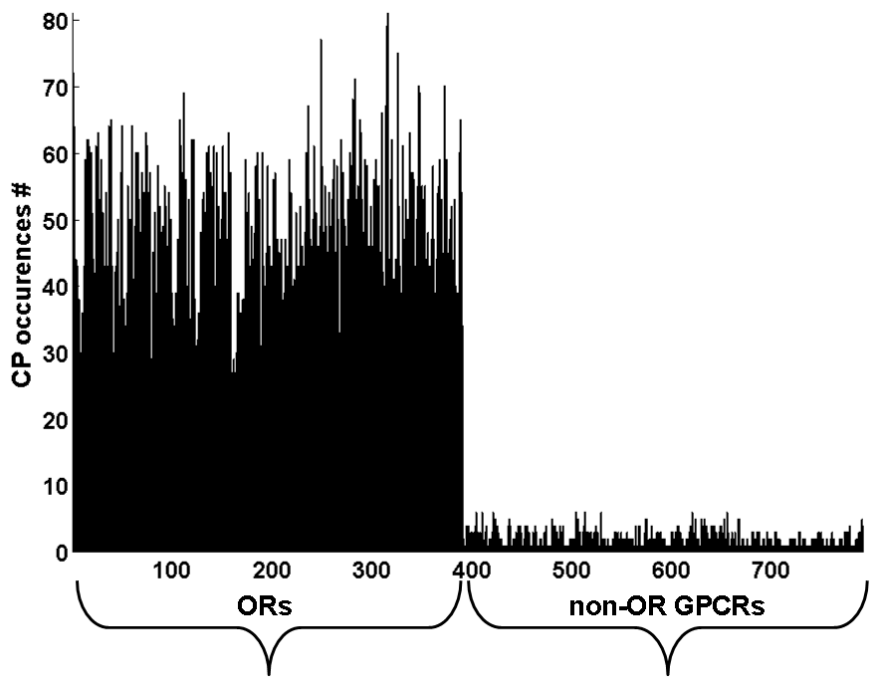
**CP occurrences on human GPCRs**. The number of CP occurrences (hits) for each of the 391 human ORs (ordered by HORDE) and, followed by 400 human non-OR GPCRs (ordered by [14]).

ORs are part of a larger protein superfamily of G-Protein Coupled Receptors (GPCRs). We searched 967 chicken, human and mouse non-OR GPCRs taken from [17] and [18] and found 526 of the OR CPs to appear in this dataset (figure 2). The number of CP occurrences (hits) on an OR is easily distinguishable from other GPCRs. The number of CP hits on non-OR GPCRs exceeds that of a random model, from which one expects to observe at most one or two CP hits. Our observation of up to 6 CP hits for some non-OR GPCRs indicates an ancestral relation between ORs and some non-OR GPCRs, i.e. remote homology (see histograms S6–S9 in Additional file 1] and explanation of the random model in the Methods section).

Figures S1 and S2 are histograms of the same kind for chicken and mouse respectively.

In figures S3–S5 we study the loci of OR CPs on non-OR GPCRs in chicken and mammals respectively. Sharp peaks in mammals correspond to known motifs [19]. No sharp peaks are observed in chicken.

### Locations of CPs on the OR sequence

**Figure 3:**
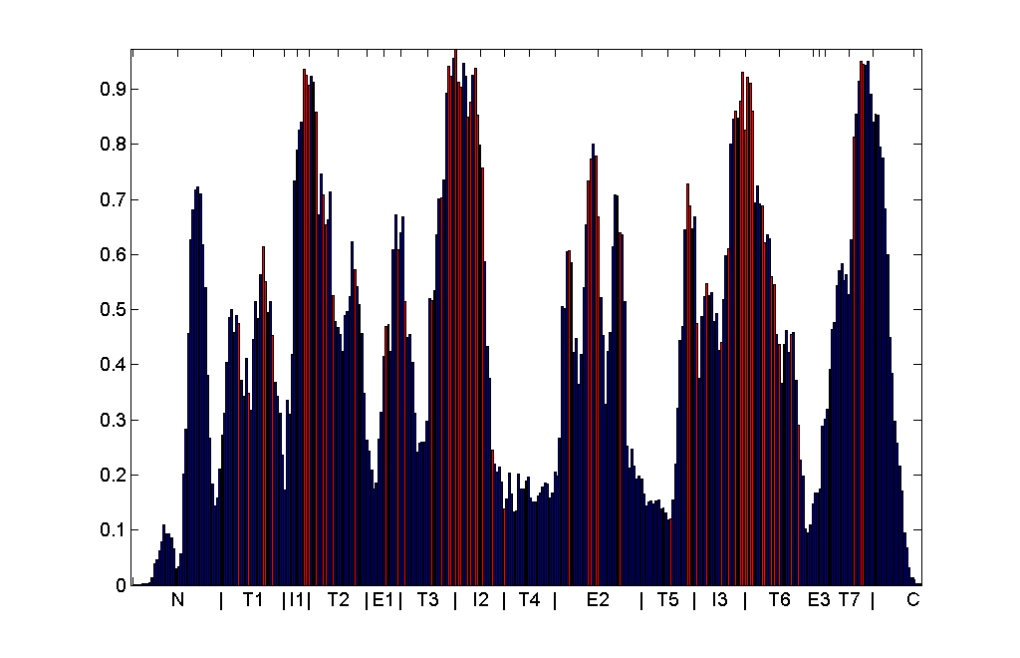
**CP coverage of positions along the OR sequence**. Positions start from the N-terminal (N), through Transmembrane domains 1–7 (T1–T7), Intracellular loops (I1–I3) and extracellular loops (E1–E3) and end in the C-terminal (C). 65 known highly-conserved positions are indicated by red.

We investigate the locations of the CPs along the 7 trans-membrane (TM) domains. The resulting histograms are compared with conservation loci of single amino-acids [20]. Locations are determined relative to a highly curated multiple alignment of human and mouse ORs. The histogram in figure 3 displays the relative coverage by CPs of each position along the OR chain (see Methods section 3.4 for a description of normalization of positions between ORs). Highly conserved positions of amino-acids, as deduced by [20] from mouse and dog data, are indicated by red coloring of the histogram on 65 positions.

**Figure 4:**
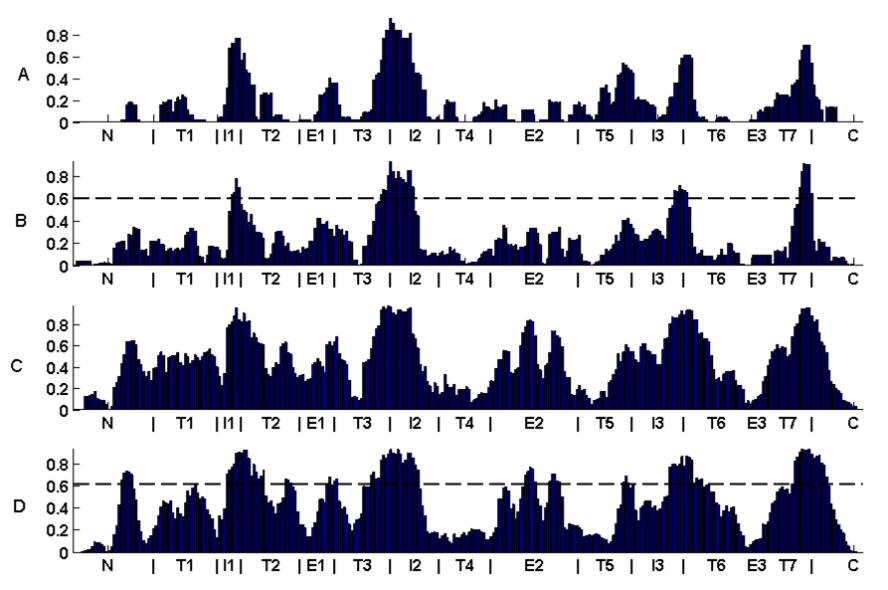
**CP coverage of positions along the OR sequence for selected species**. CPs coverage of positions along the OR sequence for pufferfish (A), zebrafish (B), Frog (C) and Human (D). Thresholds mark the regions that are common to all ten species (B) and new to vertebrates (D). Positions are the same as in Figure. 3.

Figure 4 shows the CP position coverage for four species. Figures displaying all CP positions for these three species, all other species, assessed ancestor CPs, novel and lost CPs, are provided in (figures S10–S15) [see Additional file 1].

Figure 4 indicates four regions which are highly populated with CPs along all vertebrate evolution. These regions are marked using a threshold drawn at 60% sequence population in zebrafish, displayed in figure 4B. All four regions reside in the interface between the transmembrane domains and the intracellular regions (IL1–3 and the C-terminal). These regions may be connected to structural constraints in the interface that binds the G-proteins. Figures displaying OR coverage by position for all other species ranging from frog to human look very similar (figures S10, S11 [see Additional file 1]). We observe that CPs within some regions have developed much higher coverage only in tetrapods. These regions are marked in figure 4D. They are: the end of the N-terminal, the interface between extracellular loop 1 (EL1) and TM1 and TM2 and the middle of extracellular loop 2 (EL2). Most of the newly emerged regions are facing the extracellular side of the membrane. This imposes structural constraints on the regions connected to odorant binding and might be specific to airborne odorants.

### CP-space reveals internal clusters

Using biclustering, we obtain simultaneous co-occurrences of ORs and CPs for each species. This provides a powerful visualization and allows the study of evolutionary trends across species. Details of the biclustering algorithm and its application are found in the Methods section.

We perform the analysis using different sets of CPs characterized by their evolutionary ages.

**Figure 5:**
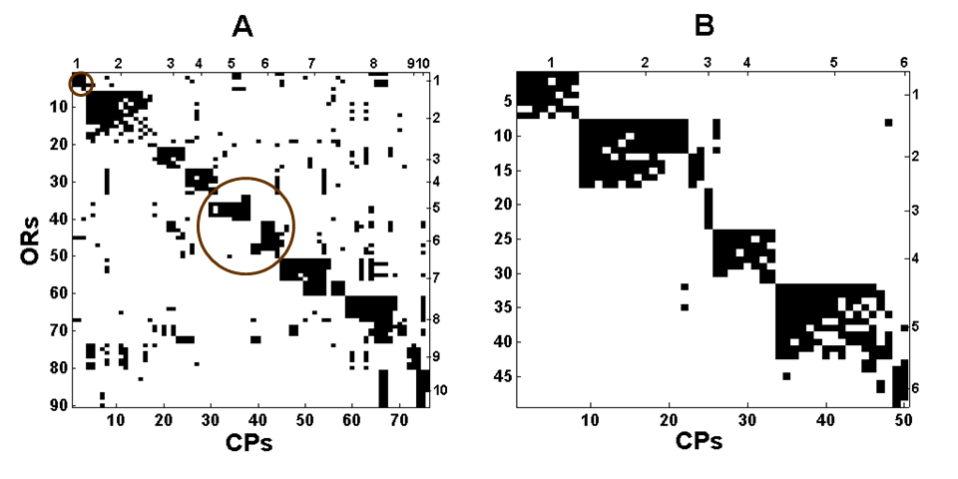
**Biclustering results of zebrafish**. Y-axis corresponds to ORs and X-axis to (A) A1 (root ancestor) CPs and (B) zebrafish novel CPs. Circled clusters in (A) have no corresponding biclusters of novel CPs in B.

First, we apply the procedure to zebrafish ORs, represented either by the *conserved CPs*, i.e. CPs shared with tetrapods (A1) or by zebrafish novel CPs (see figure 1 for reference). There are only nine CPs novel to A8 (the common ancestor of zebrafish and pufferfish) hence they are not used in the clustering analysis. The results are displayed in figure 5. We identify an interesting pattern in this figure. Zebrafish novel CPs form almost disjoint biclusters, while OR clusters based on conserved CPs (CPs originating high in the tree) tend to share CPs (figure. 5A). Conserved CPs cover almost all ORs (seven ORs did not pass the threshold of minimal CP number specified in the Methods section). Novel CPs cover only half of the ORs.

We identify ten clusters in zebrafish using ancestral (A1) CPs and six using zebrafish-novel CPs. Each of the latter six clusters matches one of the former clusters. The detailed cluster assignments are displayed in the supplementary material [see Additional file 1].

Novel CPs emerge from speciation and duplication events occurring after the split of fish from A1. We find 10 ORs that do not have any novel CPs in zebrafish and fish common ancestor (A8). This can serve as a first estimate of the number of ORs that existed in A1. They reside in the OR clusters indicated by red circles in Figure. 5A.

Classification of zebrafish ORs into groups has been studied by [6] and [21]. Both found eight groups with different OR membership (four groups of [6] and one of [21] contain only one OR each). Biclusters of novel CPs (Figure. 5B) map perfectly to some groups (groups δ, ζ and η of [6]), where some groups are further split to reveal finer details (e.g. groups δ and ζ of [6] and group E of [21] are split into two biclusters). The 10 ORs which contain no novel CPs have members only from groups δ, θ and κ of [6]. For mapping between our clusters, and the groups of [6] and [21], see additional files 2, 3 and 4.

The biclustering algorithm allows us also to differentiate between the different zebrafish clusters. The assumption is that OR clusters which relate to recent ancestry might also bear functional similarity. While some of the CPs that differentiate between the OR clusters are conserved remnants of duplication events, other CPs represent segments of these ORs that might contribute to a common functionality of the OR cluster. A table of the CPs of each cluster is provided [see Additional file 5].

Pufferfish has few novel CPs. Biclusters formed using CPs belonging to A1 look similar to the ones displayed in Figure 5A. The biclustering of pufferfish appears in figure S16 [see Additional file 1].

**Figure 6:**
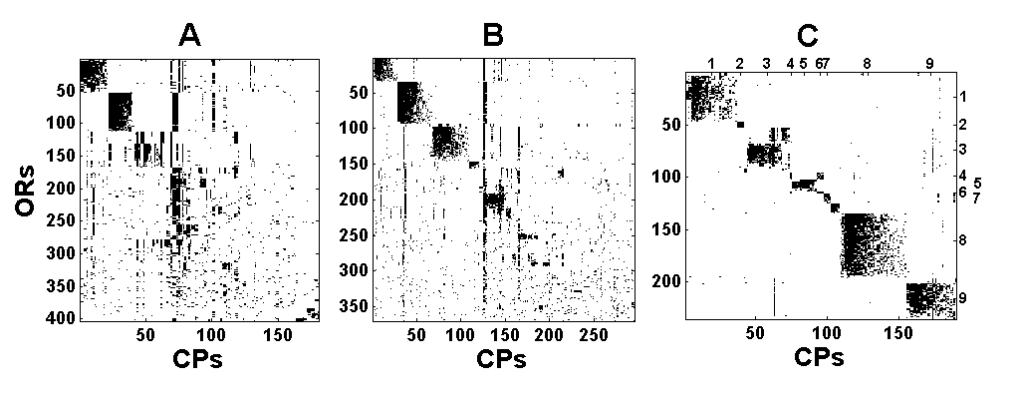
**Biclustering results of Frog**. Y-axis corresponds to ORs and X-axis to CPs novel to A1 (A), to A2 (B) and CPs novel to frog (C).

Figure 6 displays biclustering results of frog. Three sets of CPs are being used, those novel to A1, novel to the tetrapods' ancestor (A2) and novel to frog. Ancestral CPs form noisy clusters, while CPs novel to frog form almost disjoint clusters, similar to the zebrafish biclusters. As in zebrafish, the number of ORs covered by CPs drops with the age of the CP (i.e. the node in the ToL where it first appears). We identify nine clusters using CPs novel to frog. They map almost perfectly to clusters identified using either novel CPs of A1 or A2 [see Additional file 3].

Unlike zebrafish clusters, not all the A1 and A2 conserved CPs form identifiable biclusters. This suggests that they have been subjected to a higher mutation rate than observed in zebrafish, which may relate to the appearance of class II ORs in frog [22]. The clusters in figure 6c relate to the groups γ and δ of [6], [see Additional file 4].

Chicken and lizard have too few novel A3 and A7 CPs, to construct biclusters. The novel CPs of chicken form one big cluster, while novel CPs of lizard form small disjoint clusters. Novel CPs to A1 and A2 also show difference between chicken and lizard. While the former reveals a robust big cluster, the latter show no clusters at all. This implies large number of recent duplications in chicken. The biclustering of chicken and lizard appear in figures S17–S18 [see Additional file 1].

Biclusters in mammals are displayed in figures S19–S23 [see Additional file 1]. Biclusters are significant for CPs novel to A3–A6. They can be mapped to class I (fish-like) and class II (mammals-like) ORs, and to families of the Human Olfactory Receptor Data Explorer (HORDE). The mapping appears in Additional files 6, 7, 8, 9, 10, 11 and 12.

### Novel CPs and mammalian families

**Figure 7:**
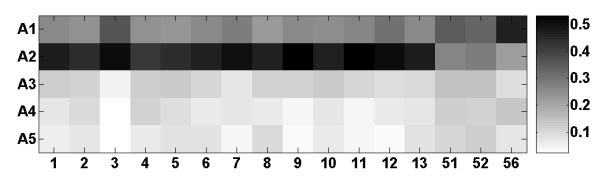
**Distribution of CP age, novel to A1–A5 ancestors for each mammalian HORDE family**. X-axis corresponds to family number. Color scale corresponds to percentage from the total number of CPs of each family, ranging from 0 (white) to 1 (black).

Figure 7 shows the correspondence between mammalian CPs and the classification of the OR superfamily into families, using the HORDE classification system [15]. Class II (families 1–13) ORs contain predominantly CPs of A2. In contrast, class I (families 51, 52 and 56) ORs have equal distribution of novel CPs from A1 and A2. We also observe that family 3 almost ceased to evolve after A2 and families 9 and 11 stopped evolving after A3.

## Discussion and conclusion

We use CPs extracted by MEX (Motif Extraction algorithm) to study evolutionary processes in olfactory receptors. Such conserved CPs are known to have biological importance [23] and are expected to play structural and functional roles in olfactory receptors. Having extracted such CPs from ten species, we use evolutionary constraints to further employ the extracted CPs in making sense of the complex relationships of ORs of different species with one another.

The evolutionary perspective is obtained by applying the parsimony principle to a tree-of-life accommodating the studied species. It allows us to construct an ancestral phyletic pattern of the presence or absence of CPs in internal nodes of the tree. Using this construction, we show that the number of species-specific CPs is relatively high in fish and frog, but remains fixed in terrestrial species. The species-specific CPs in the aquatic species might be related to ORs detecting water-soluble odorants. We observe a major emergence of CPs in the ancestor of tetrapods and major losses of CPs in pufferfish and in chicken. A surprising result stemming from this mapping is that although humans lost half of the intact mammalian ORs, they lost only 11% of the conserved CPs, suggesting a controlled process of loss of redundant ORs. In other words, the potential odorant recognition of humans may have suffered only a minor damage by the severe diminution of their OR repertoire.

CPs that differentiate between water-dwelling species and terrestrial species have potential biological significance and are candidates for further biochemical studies.

We show that some of the OR-extracted CPs exist in the general GPCR population, demonstrating the ancient origin of ORs and several other GPCRs.

The fact that the OR history stretches back to fish was made by [6] who claimed that 85%–90% of frog, chicken, mouse and human OR repertoires was constructed from duplication of a single fish OR of group γ, Dr3OR5.4. One or more of these 35 fish group γ CPs are also observed in 98% of the tetrapod ORs. This is larger than the coverage observed for CPs in any other fish ORs. These 35 CPs are also almost exclusively located in the five most conserved positions in figure 3 (boundary between IL1 and TM2, boundary between IL2 and TM3, middle of EL2, boundary between IL3 and TM6 and TM7). We point out, however, that major changes have occurred in other nodes of evolutionary history. By studying loci of CPs we identify two regions that show high CP coverage starting from tetrapods: the N-terminal and the middle of the second extracellular loop. This might imply that these regions are important for the adaptation of ORs to airborne odorants.

Gene multiplication events are most naturally exhibited by the existence of clusters of ORs. Using the evolutionary separation into novel and conserved CPs, we are able to demonstrate clean OR clusters. This is done by applying a biclustering algorithm to matrices associating CPs with ORs within species: clean clusters emerge when novel CPs are being employed. Results vary with increasing evolutionary age of the species in question. Our biclustering results of the species studied by [6,21] (zebrafish, frog and chicken) generally support their phylogenetic models, but provide finer OR grouping and a cleaner selection of the responsible ancestor (where CP formation has occurred). Finally, we are able to use the CP analysis to provide developmental details of OR families of the Human Olfactory Receptor Data Explorer (HORDE).

## Methods

### Data

For the described study we selected a set of 4027 intact olfactory receptors (ORs) from ten vertebrate species including pufferfish (*Takifugu rubripes*), zebrafish (*Danio rerio*), frog (*Xenopus tropicalis*), chicken (*Gallus gallus*), lizard (*Anolis carolinensis*), platypus (*Ornithorhynchus anatinus*), opossum (*Monodelphis domestica*), dog (*Canis familiaris*), mouse (*Mus musculus*) and human (*Homo sapiens*).

All mammalian, chicken and lizard OR sequences are available at the HORDE [15]. OR sequences of fish and frog were taken from the study of [6]. Lizard and Platypus ORs appear in [24]. The number of ORs for each species is listed in Table 1.

967 chicken, human and mouse non-OR GPCRs were taken from [17] and [18].

### MEX algorithm

MEX is a motif extraction algorithm introduced by [9] as part of a method for grammar induction from texts and was later used on proteins [10]. Given a set of proteins, they are represented as different paths over a graph that consists of 20 vertices, corresponding to the 'alphabet' of 20 amino-acids. MEX proceeds by looking for convergence of many paths onto strings of amino-acids, and the subsequent divergence from such strings. The latter are defined as motifs if both convergence and divergence obey some statistical conditions. These conditions are imposed on context-dependent variable-order Markov chains that are constructed out of the data-paths. The algorithm has two parameters, η and α, specifying the amount of convergence/divergence and its statistical significance given the number of paths involved in the process. More information can be found on the website [25].

In the present analysis we ran MEX on the proteins of each species separately, using the parameter values η = 0.9 and α = 0.01. We restricted ourselves to peptides of length 5 amino-acids or more and appearing in at least 4 ORs. These peptides were merged into one list, where duplicates and peptides containing other peptides were removed. The resulting non-redundant list contains 2717 Common Peptides (CPs). Each of the CPs was then searched on the ORs of all species. CPs that appear only in the ORs of one of the studied species are defined to be *species-specific*.

### Fitting CPs to the tree of life and phylogenetic analysis

We used the tree of life web project, available at [26] to construct the relationships between the species. The relations between the species is consistent with the tree of life of [14]. Dog, Mouse and Human were put under one common ancestor according to the tree of life web project, although there are other possible ancestral orders based on different set of genes (see also[27,28]-[29]). Trying other arrangements for Dog, Mouse and Human did not alter the derived conclusions. The assessment of CP origins uses the Wagner parsimony, as implemented by the Phylogeny Inference Package computer programs PHYLIP. Similar results are also obtained by Dollo parsimony.

Since some CPs differ by only one amino acid from others, we have also checked whether loss and gain of a CP on any internal node corresponds to a mutation of a single amino-acid (interpreted as a loss of the CP) into another amino-acid (interpreted as a gain of a CP). We have found that the number of such events is negligible (1 such event in an ancestral node on average and 7 on average in the species, occurring mainly in chicken and lizard).

Following Parsimony estimation, each internal node A1–A8, and each species, has a list of CPs associated with it. We identify "*novel CPs*" as those that exist in the current ancestor/species but did not exist in previous ancestors and "*lost CPs*" are defined as those that exist in the current ancestor/species but did exist in the previous ancestor. CPs that date back to previous ancestors are referred to as "*conserved CPs"*.

### Normalizing CP positions

Each CP contains a set of positions relative to the start of each OR. Due to variable N-Terminal length and gaps, we needed to normalize the different positions of each CP appearing in different ORs. We normalized the OR relative positions using ClustalW2 (available at [30]). We first aligned the five sequences used in [31] to construct a profile (replacing MOR257-1 that was not available in our set with MOR257-10). Each OR was then aligned to this profile.

### Biclustering

Biclustering is performed on the ORs of each species, using subsets of CPs, each subset corresponding to a different origin on the tree of life. Each OR is represented by a binary vector that signifies the existence or non-existence of each of the CPs on its sequence. In order to clear noise, we first removed all ORs having less than 5 CPs from the relevant tree of life node. We then removed CPs that appear in less than 5 ORs from the remaining set. ORs left with no CPs after the previous removal were also removed. We used a bipartite spectral graph partitioning algorithm of [32]. Initially designed for documents and words, this bi-clustering algorithm handles sparse data well. This algorithm produces biclusters of ORs and CPs. We augmented the algorithm to produce good biclusters' images. This was achieved by applying single linkage hierarchical algorithm for each produced bicluster and sorting each bicluster according to the hierarchical clustering, thus handling less homogenous clusters better. This augmentation of the algorithm does not alter the assignment of ORs and CPs to biclusters, but merely provides better visualization of the biclusters.

## Authors' contributions

AG carried out the CP extraction from ORs, analyzed the results and drafted the manuscript. TO supplied the data, contributed to the analysis and presentation of the results and reviewed the manuscript. DL and DH designed this collaborative project and helped analyze the results. DH participated in drafting the manuscript. All authors read and approved the final manuscript.

## Supplementary Material

Additional file 1 **Additional figures and graphs**. Additional figures and graphs. Click here for file

Additional file 2 **Zebrafish ORs CP numbers and cluster assignment**. Number of CPs from each ancestor occurring in each zebrafish OR and cluster assignment for each zebrafish OR. Click here for file

Additional file 3 **Frog ORs CP numbers and cluster assignment**. Number of CPs from each ancestor occurring in each Frog OR and cluster assignment for each Frog OR. Click here for file

Additional file 4 **Chicken ORs CP numbers and cluster assignment**. Number of CPs from each ancestor occurring in each Chicken OR and cluster assignment for each Chicken OR. Click here for file

Additional file 5 **Zebrafish CPs locations and cluster assignment**. Zebrafish CPs with sequence locations and cluster assignments. Click here for file

Additional file 6 **Opossum ORs CP numbers and cluster assignment**. Number of CPs from each ancestor occurring in each Opossum OR and cluster assignment for each Opossum OR. Click here for file

Additional file 7 **Dog ORs CP numbers and cluster assignment**. Number of CPs from each ancestor occurring in each Dog OR and cluster assignment for each Dog OR. Click here for file

Additional file 8 **Mouse ORs CP numbers and cluster assignment**. Number of CPs from each ancestor occurring in each Mouse OR and cluster assignment for each Mouse OR. Click here for file

Additional file 9 **Human ORs CP numbers and cluster assignment**. Number of CPs from each ancestor occurring in each Human OR and cluster assignment for each Human OR. Click here for file

Additional file 10 **Pufferfish ORs CP numbers and cluster assignment**. Number of CPs from each ancestor occurring in each pufferfish OR and cluster assignment for each pufferfish OR. Click here for file

Additional file 11 **Lizard ORs CP numbers and cluster assignment**. Number of CPs from each ancestor occurring in each Lizard OR and cluster assignment for each Lizard OR. Click here for file

Additional file 12 **Platypus ORs CP numbers and cluster assignment**. Number of CPs from each ancestor occurring in each Platypus OR and cluster assignment for each Platypus OR. Click here for file
