# Supplementary material for: Common peptides shed light on evolution of Olfactory Receptors
Source: BMC Evol Biol. 2009 May 5;9:91. doi: 10.1186/1471-2148-9-91 (PMC2681464; doi:10.1186/1471-2148-9-91)
Supplement: Additional file 1 — Additional figures and graphs. Additional figures and graphs. [file 1471-2148-9-91-S1.pdf]

Supplementary Material

Common peptides shed light on evolution of Olfactory Receptors

Assaf Gottlieb[1*], Tsviya Olender[2], Doron Lancet[2] and David Horn [1]

[1] School of Physics and Astronomy, Tel Aviv University, Tel Aviv 69978, Israel

[2] Dept of Molecular Genetics, Weizmann Institute of Science, Rehovot 76100, Israel

Abbr. CP=Common Peptide.

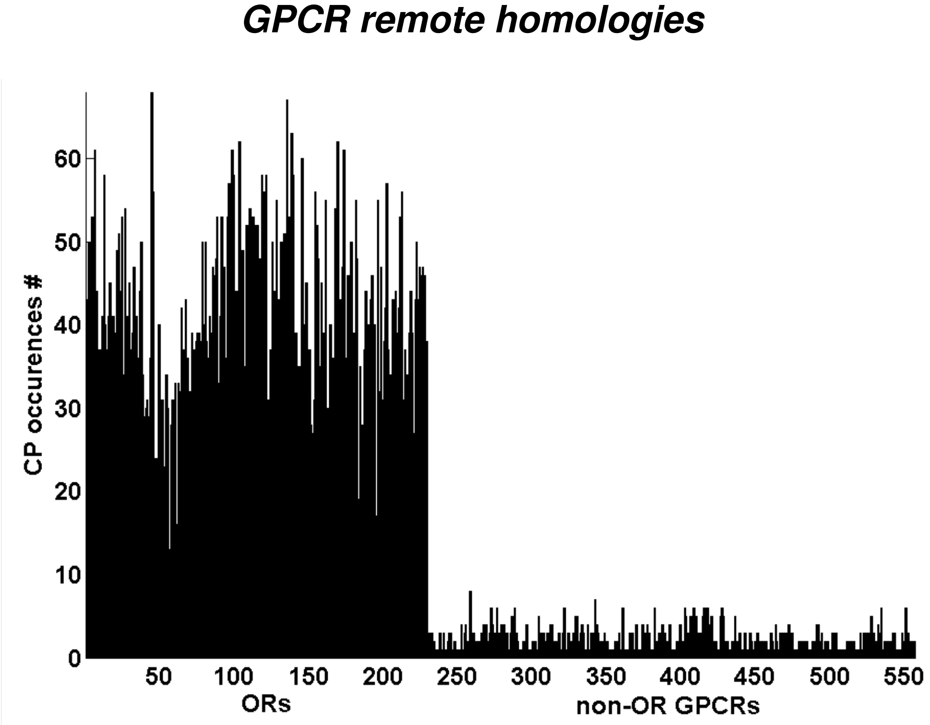

Figure S1. The number of CP occurrences (hits) for each of the chicken 229 intact and pseudogene ORs and 281 non-OR GPCR from [1]

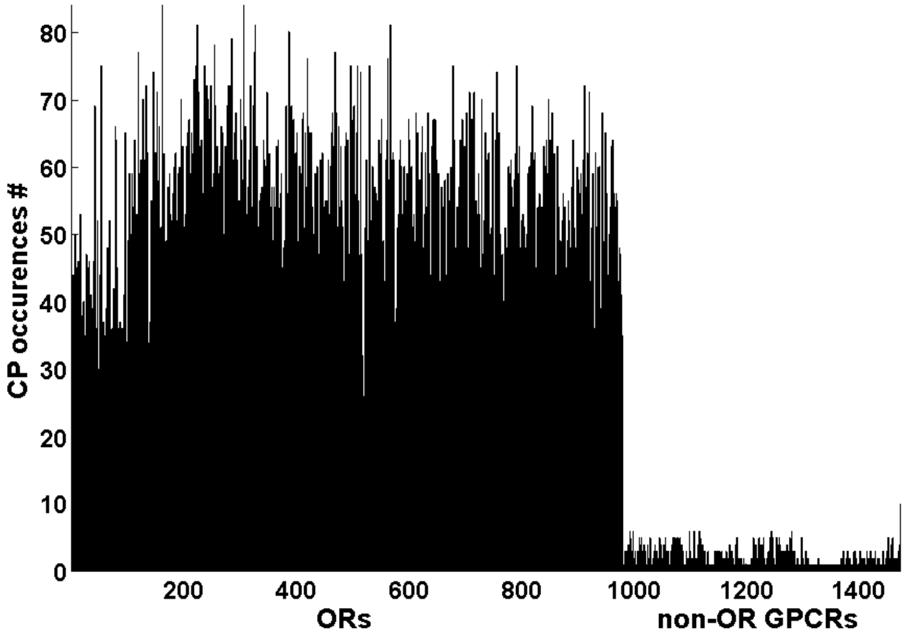

Figure S2. The number of CP occurrences (hits) for each of the mouse 978 intact ORs and 386 non-OR GPCR from [2].

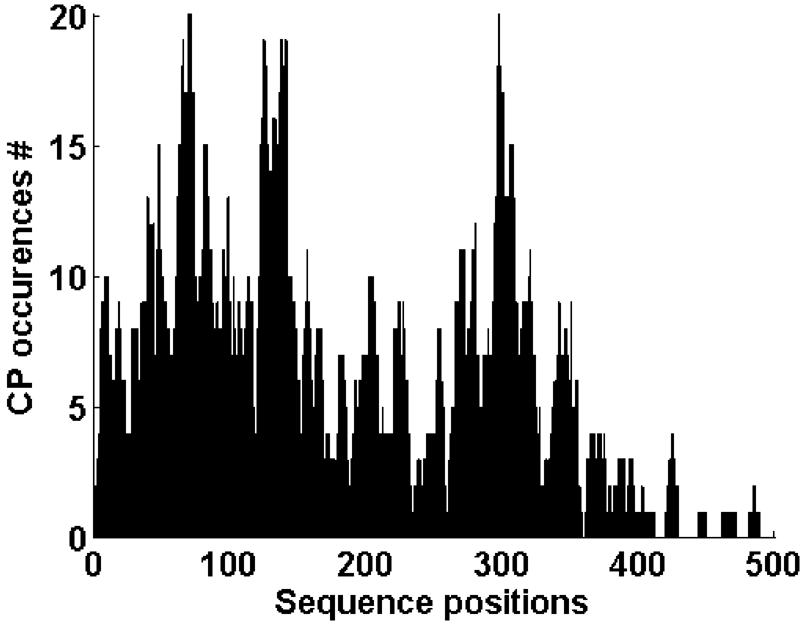

Figure S3. CP coverage of amino acid positions along chicken non-OR GPCR sequences. The positions are shown up to 500 amino-acids for clarity.

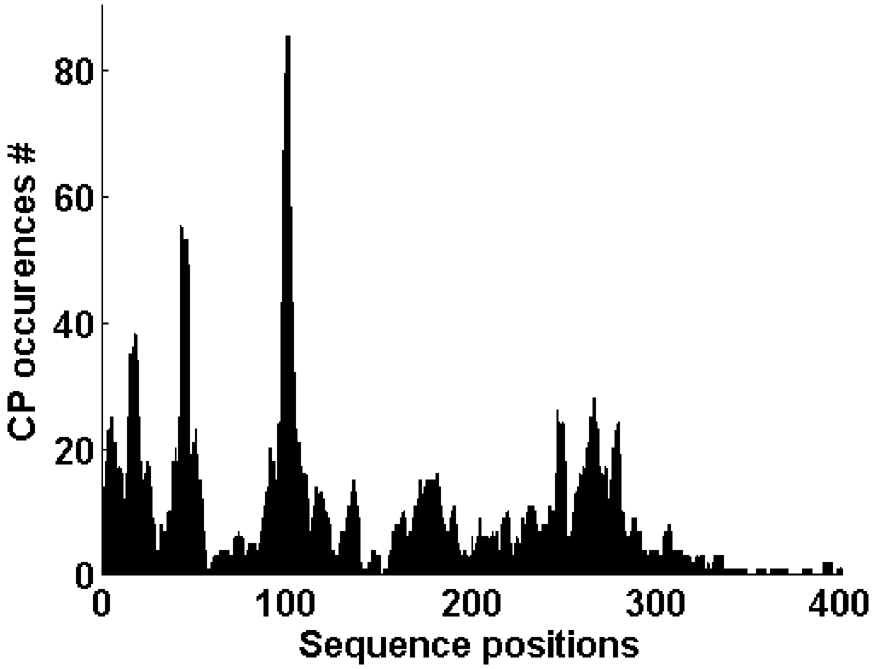

Figure S4. CP coverage of amino acid positions along mouse non-OR GPCR sequences. The positions are shown up to 400 amino-acids for clarity.

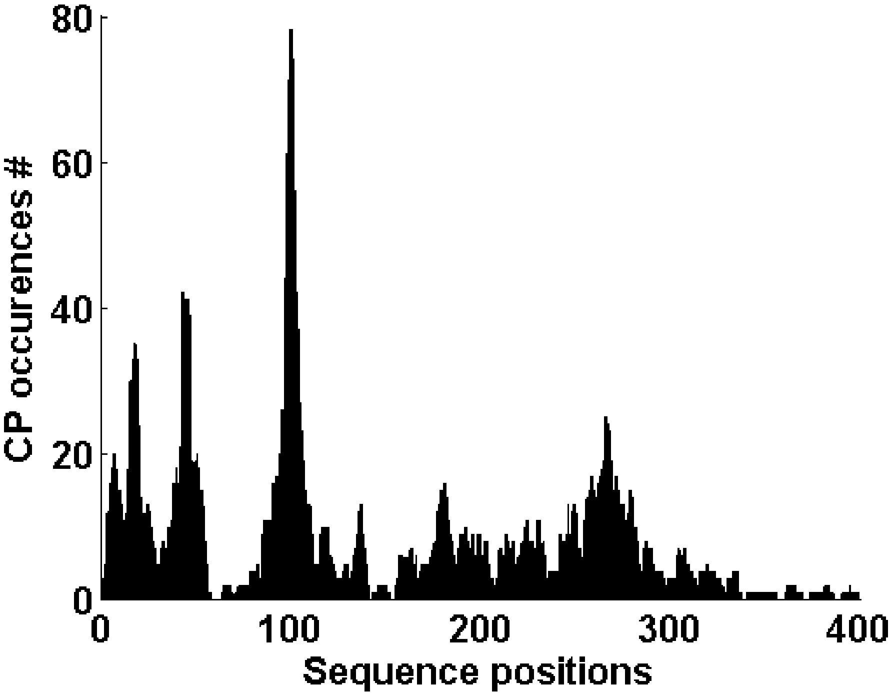

Figure S5. CP coverage of amino acid positions along mouse non-OR GPCR sequences. The positions are shown up to 400 amino-acids for clarity.

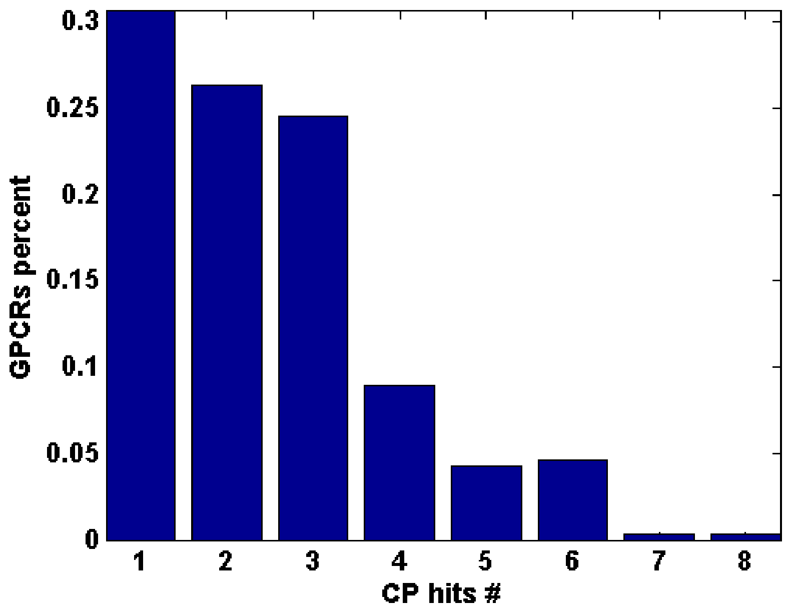

Figure S6. Histogram of the percent of chicken non-OR GPCRs as a function of the number of CPs occurring in them.

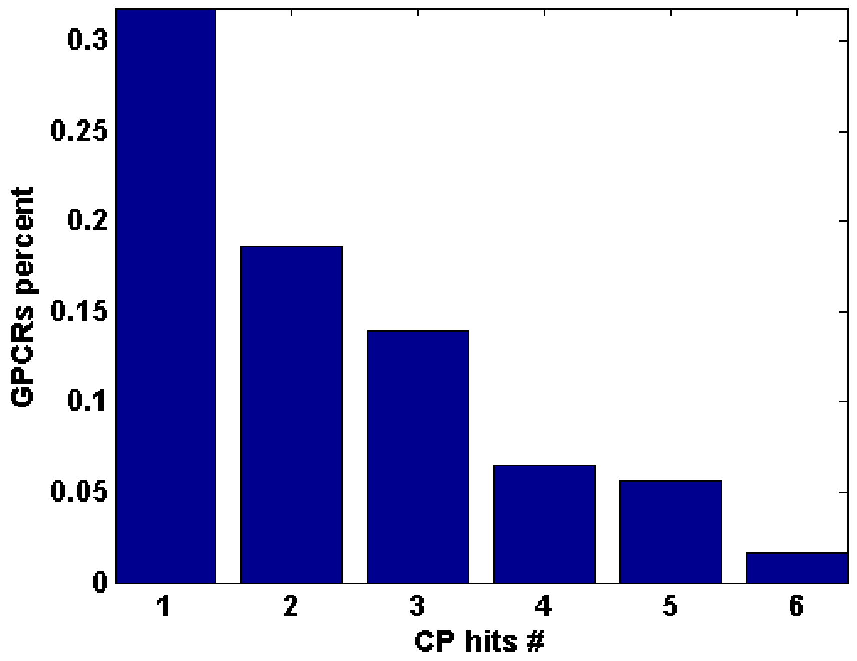

Figure S7. Histogram of the percent of mouse non-OR GPCRs as a function of the number of CPs occurring in them.

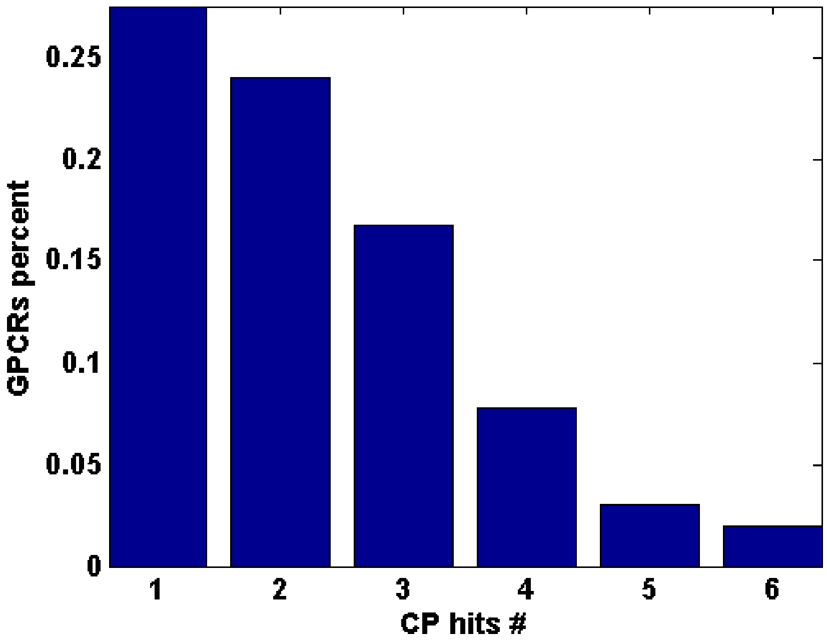

Figure S8. Histogram of the percent of human non-OR GPCRs as a function of the number of CPs occurring in them.

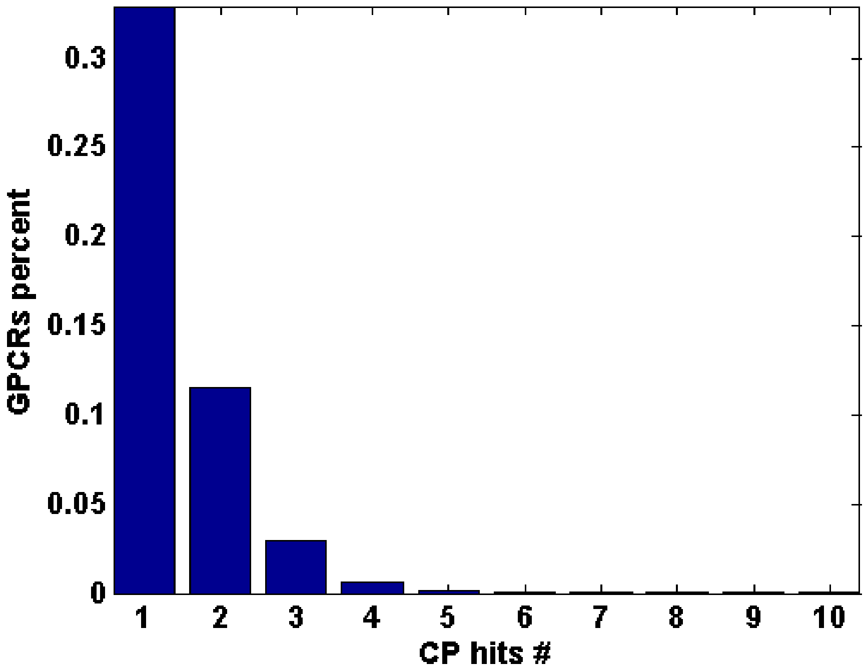

Figure S9. Histogram of the percent of human and mouse randomly permuted non-OR GPCRs as a function of the number of CPs occurring in them.

Locations of CPs on the OR sequence

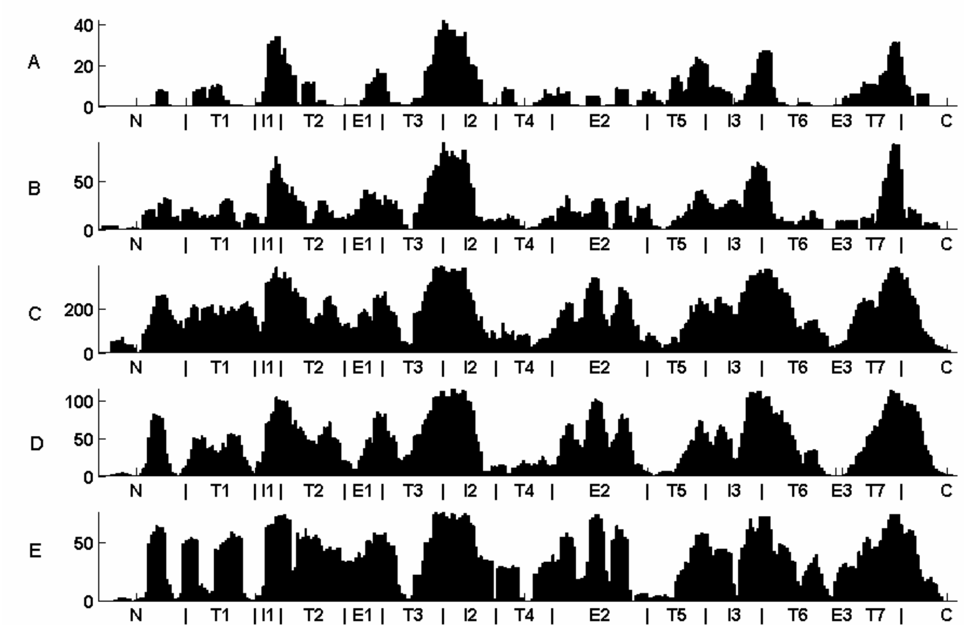

Figure S10. Coverage of ORs by CPs as a function of positions along the OR sequence for Pufferfish (A), Zebrafish (B), Frog (C), Lizard (D) and Chicken (E). Positions start from the N-terminal (N), through Transmembrane domains 1-7 (T1-T7), Intracellular loops (I1-I3) and extracellular loops (E1-E3), ending with the C-terminal (C).

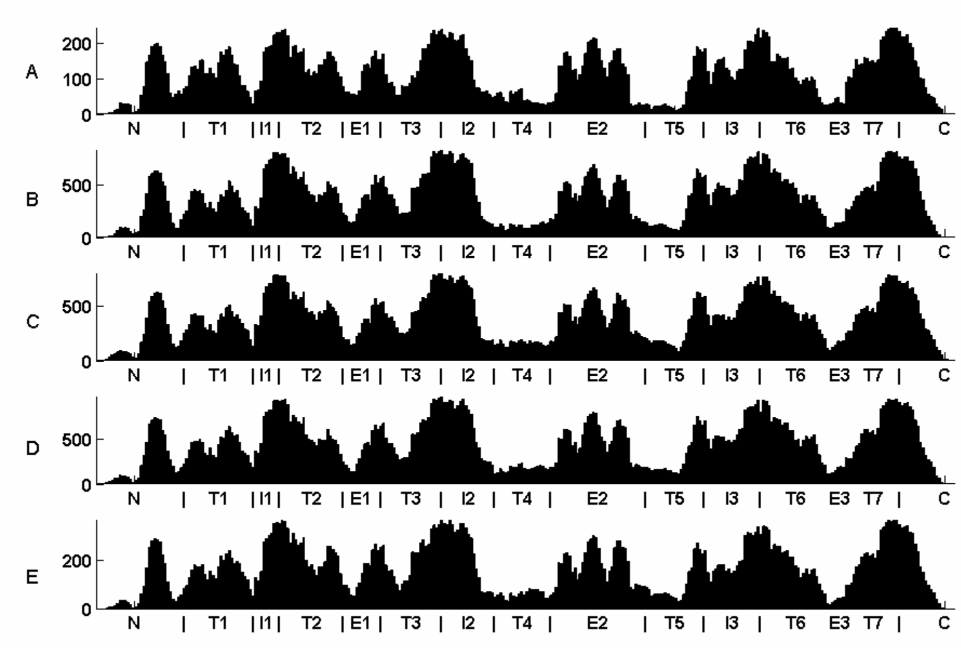

Figure S11. Coverage of ORs by CPs as a function of positions along the OR sequence for Platypus (A), Opossum (B), Dog (C), Mouse (D) and Human (E). Positions are ordered using the same coordinates as in Figure S10.

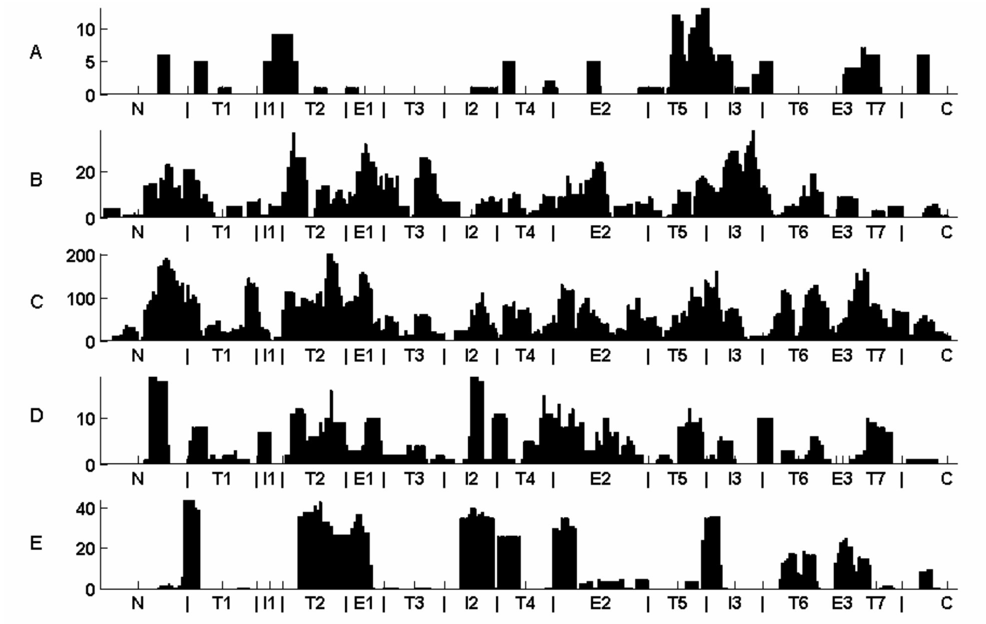

Figure S12. Coverage of ORs by CPs as a function of positions along the OR sequence for *novel* CPs of Pufferfish (A), Zebrafish (B), Frog (C), Lizard (D) and Chicken (E). Positions are ordered using the same coordinates as in Figure S10.

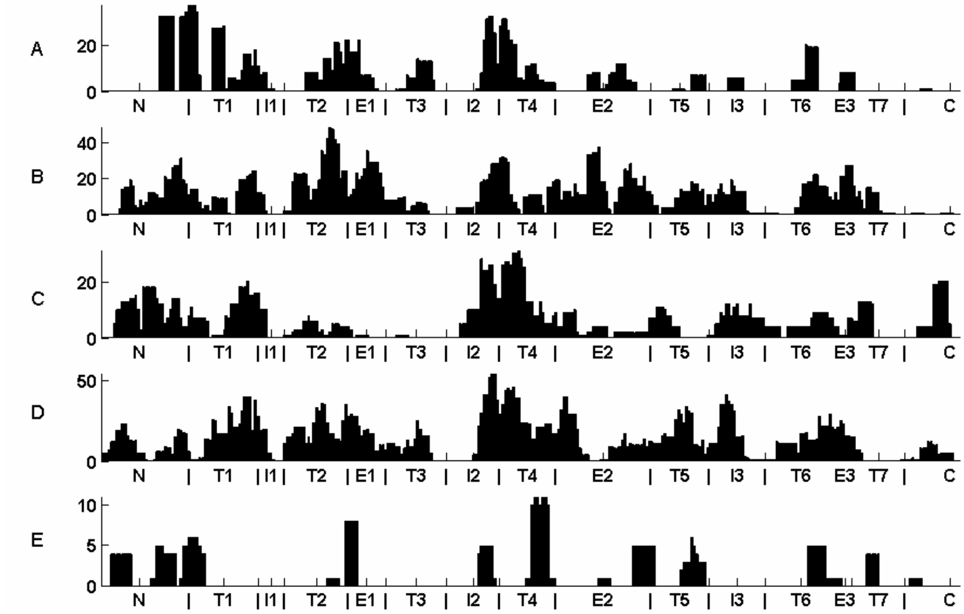

Figure S13. Coverage of ORs by CPs as a function of positions along the OR sequence for *novel* CPs of Platypus (A), Opossum (B), Dog (C), Mouse (D) and Human (E). Positions are ordered using the same coordinates as in Figure S10

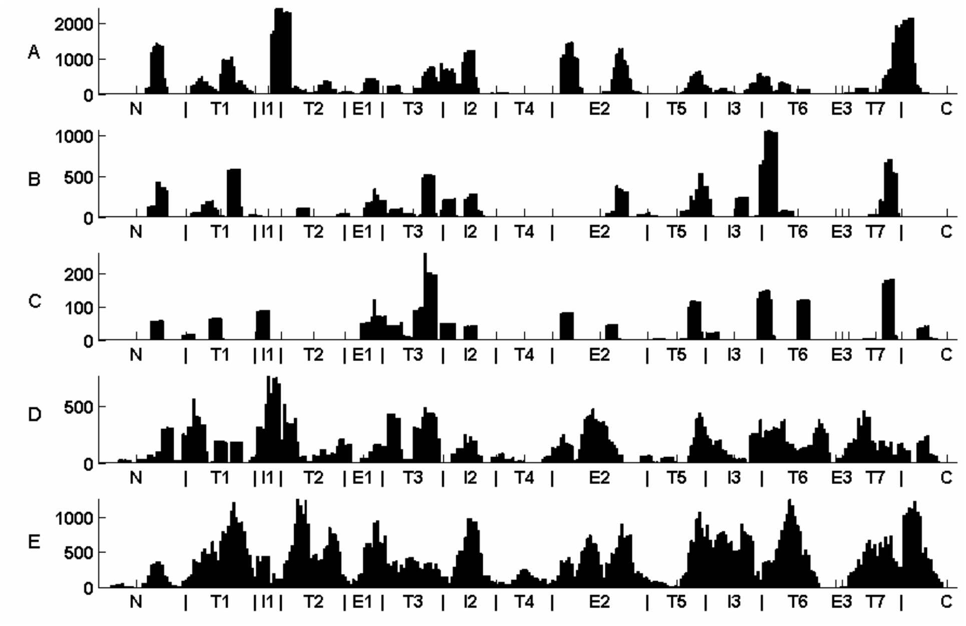

Figure S14. Coverage of ORs by CPs as a function of positions along the OR sequence for CPs *lost* in Pufferfish (A), Zebrafish (B), Frog (C), Lizard (D) and Chicken (E). Positions are calculated over all ORs other than the specific species and ordered using the same coordinates as in Figure S10.

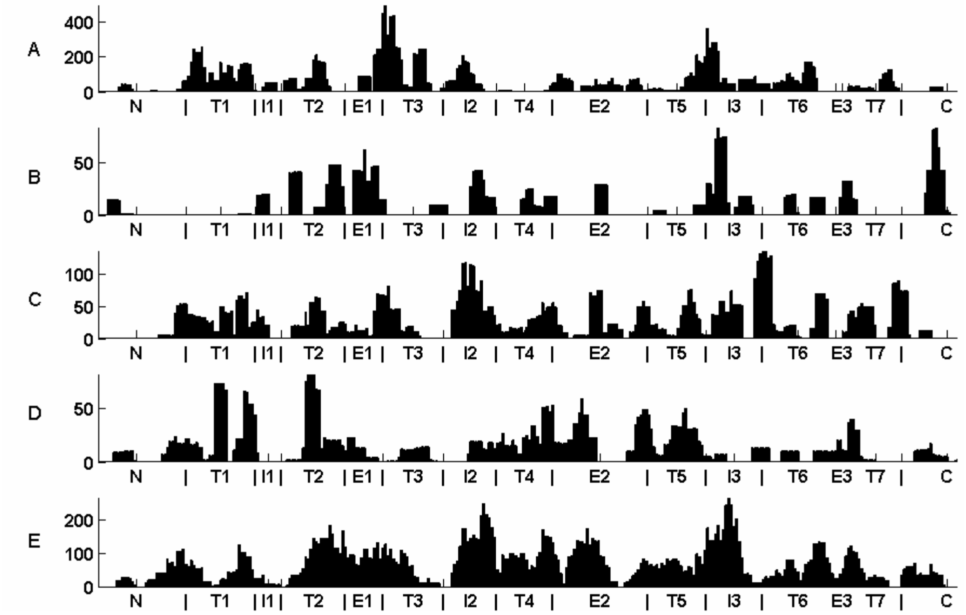

Figure S15. Coverage of ORs by CPs as a function of positions along the OR sequence for CPs *lost* in Platypus (A), Opossum (B), Dog (C), Mouse (D) and Human (E). Positions are calculated over all ORs other than the specific species and ordered using the same coordinates as in Figure S10

CP-space reveals internal clusters

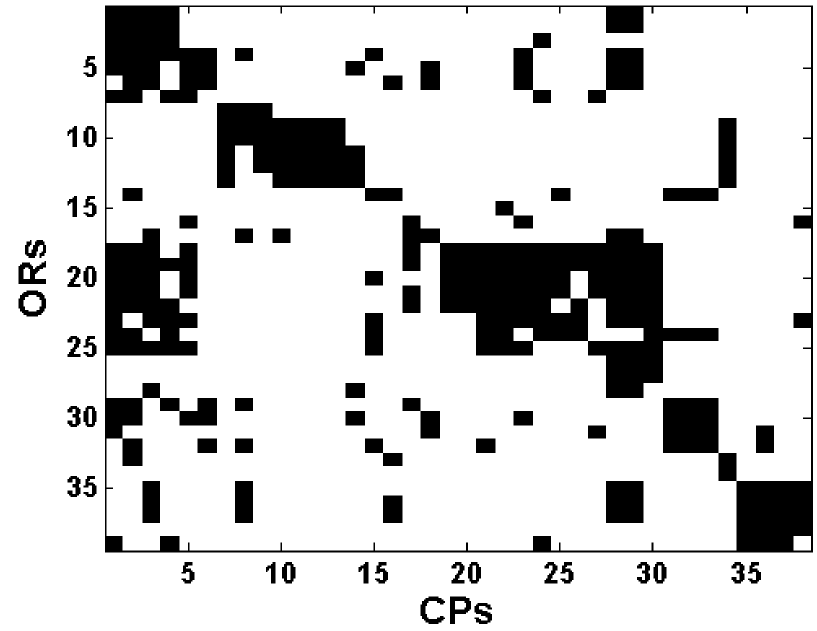

Figure S16. Biclustering results of Pufferfish. Y-axis corresponds to chicken ORs and X-axis to CPs novel to pufferfish.

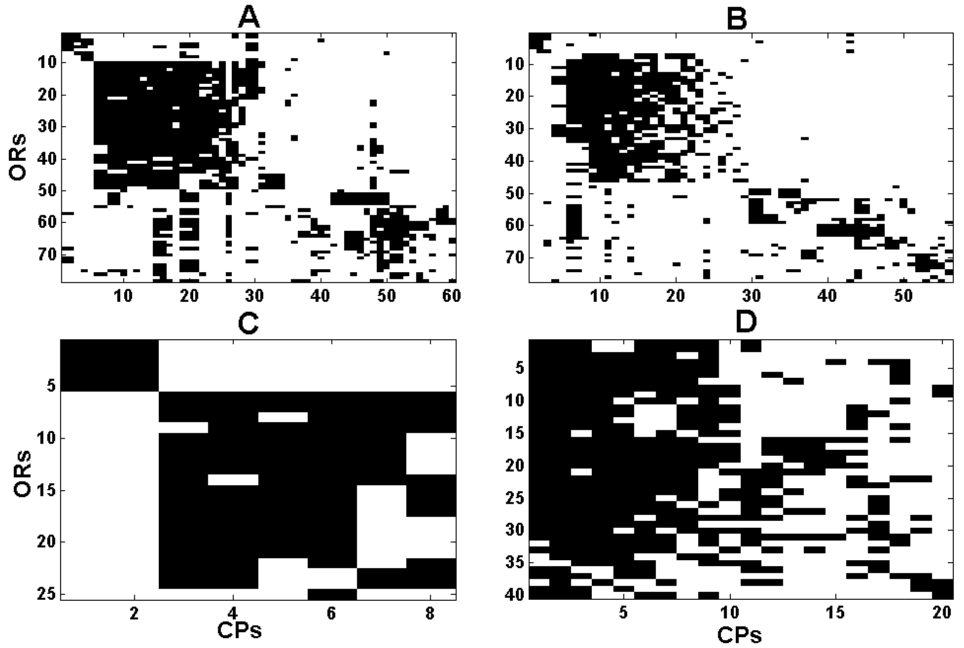

Figure S17. Biclustering results of Chicken. Y-axis corresponds to chicken ORs and X-axis to CPs novel to the MRCA of fish and tetrapods (A), tetrapods ancestor (B), amniotes ancestor (C) and CPs novel to chicken (D).

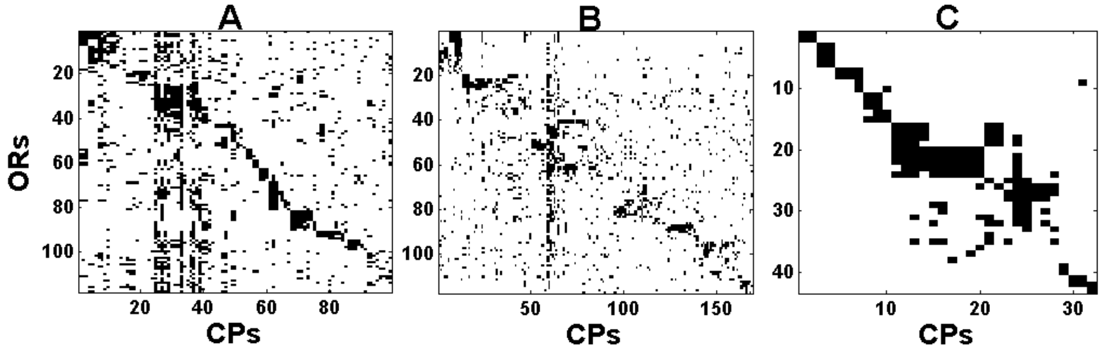

Figure S18. Biclustering results of Lizard. Y-axis corresponds to lizard ORs and X-axis to CPs novel to the MRCA of fish and tetrapods (A), ancestor of tetrapods (B) and CPs novel to lizard (C).

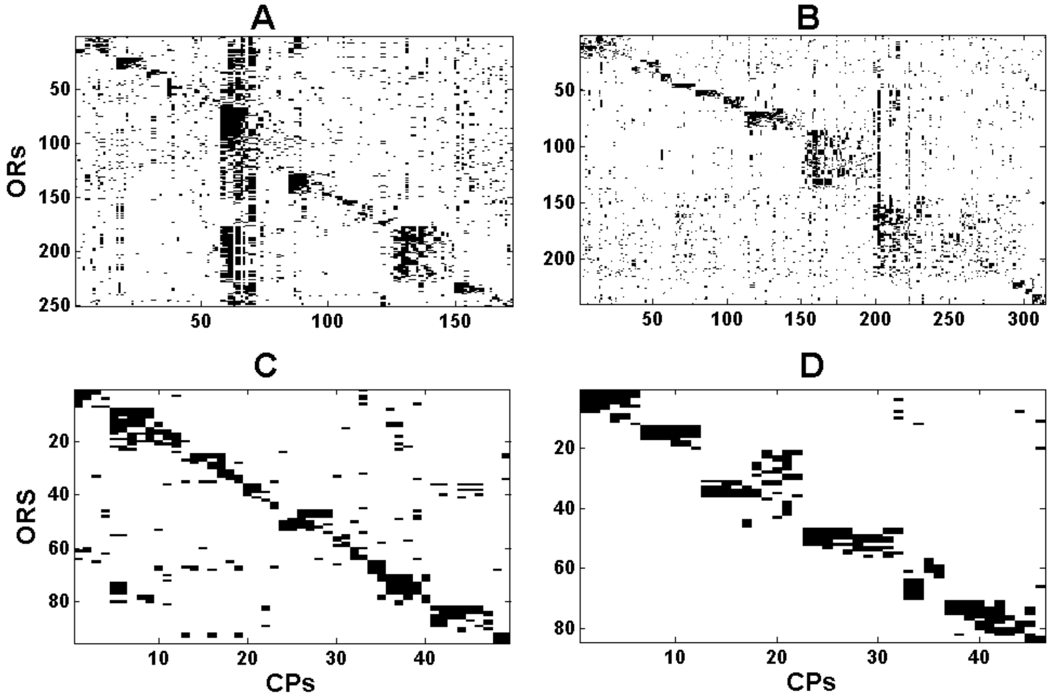

Figure S19. Biclustering results of Platypus. Y-axis corresponds to platypus ORs and X-axis to CPs novel to MRCA of fish and tetrapods (A), ancestor of tetrapods (B), ancestor of amniotes (C) and ancestor of mammals (D) CPs.

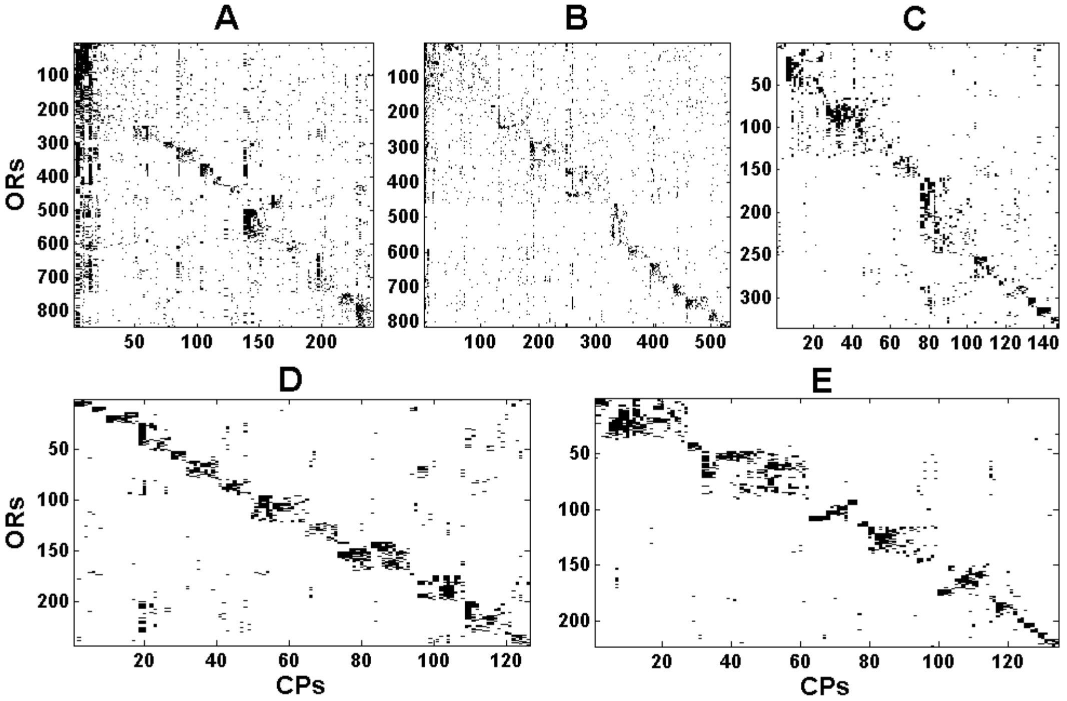

Figure S20. Biclustering results of Opossum. Y-axis corresponds to opossum ORs and X-axis to CPs novel to MRCA of fish and tetrapods (A), ancestor of tetrapods (B), ancestor of amniotes (C), ancestor of mammals (D) and ancestor of marsupials (E).

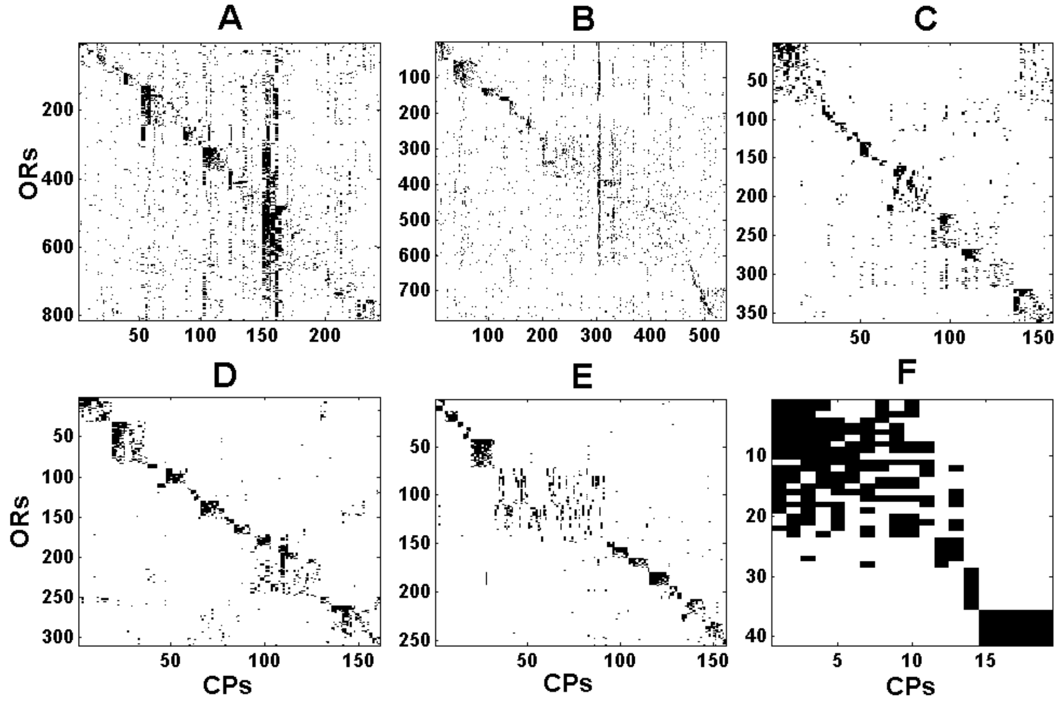

Figure S21. Biclustering results of Dog. Y-axis corresponds to dog ORs and X-axis to CPs novel to MRCA of fish and tetrapods (A), ancestor of tetrapods (B), ancestor of amniotes (C), ancestor of mammals (D), ancestor of marsupials (E) and ancestor of eutherians (F).

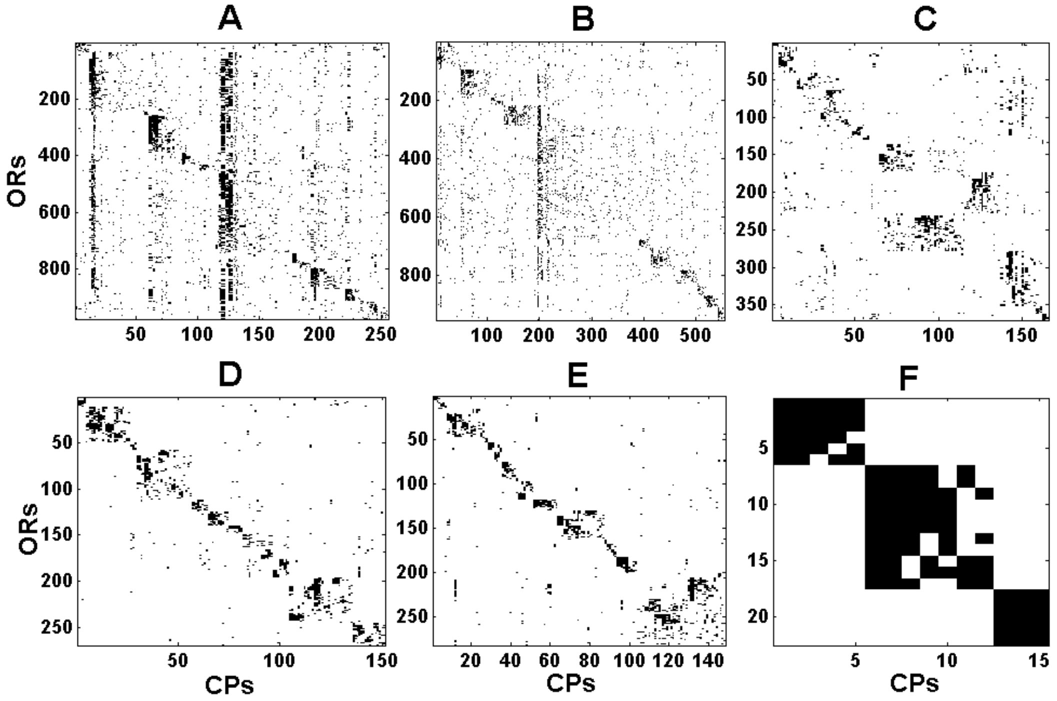

Figure S22. Biclustering results of Mouse Y-axis corresponds to mouse ORs and X-axis to CPs novel to MRCA of fish and tetrapods (A), ancestor of tetrapods (B), ancestor of amniotes (C), ancestor of mammals (D), ancestor of marsupials (E) and CPs novel to mouse (F).

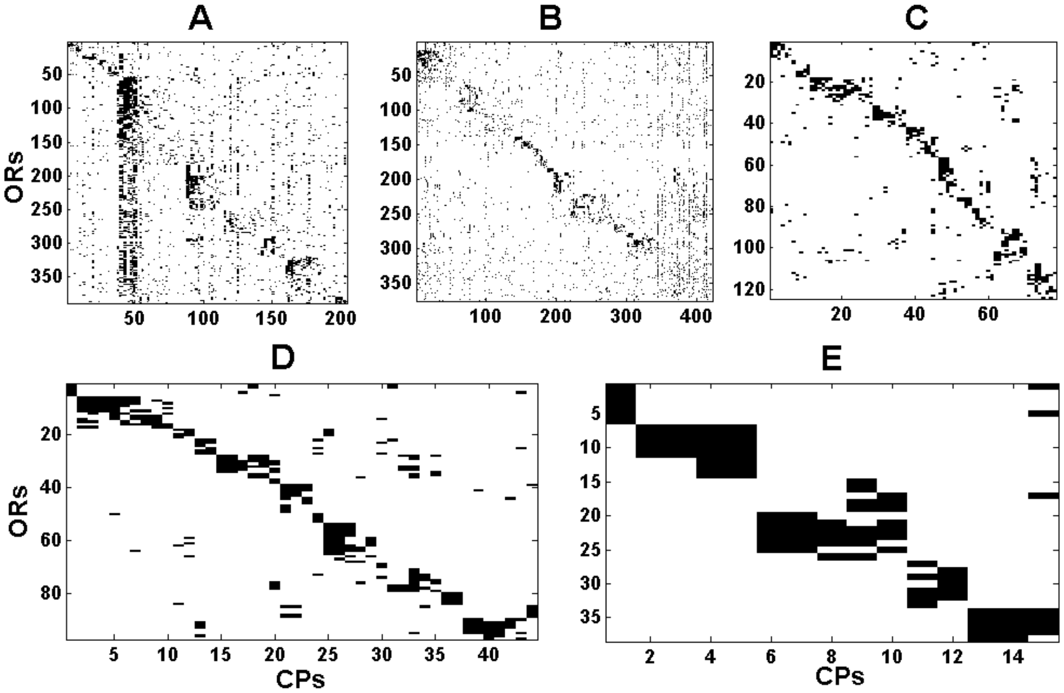

Figure S23. Biclustering results of Human. Y-axis corresponds to human ORs and X-axis to CPs novel to MRCA of fish and tetrapods (A), ancestor of tetrapods (B), ancestor of amniotes (C), ancestor of mammals (D) and ancestor of marsupials (E).

References

1. Lagerström MC, Hellström AR, Gloriam DE, Larsson TP, Schiöth HB, Fredriksson R: **The G Protein–Coupled Receptor Subset of the Chicken Genome**. *PLoS Comput Biol* 2006, **2**(6):e54.
2. Bjarnadóttir TK, Gloriam DE, Hellstrand SH, Kristiansson H, Fredriksson R, Schiöth HB: **Comprehensive repertoire and phylogenetic analysis of the G protein-coupled receptors in human and mouse**. *Genomics* 2006, **88**:263-273.
