# Supplementary material for: Common peptides shed light on evolution of Olfactory Receptors
Source: BMC Evol Biol. 2009 May 5;9:91. doi: 10.1186/1471-2148-9-91 (PMC2681464; doi:10.1186/1471-2148-9-91)
Supplement: Additional file 2 — Zebrafish ORs CP numbers and cluster assignment. Number of CPs from each ancestor occurring in each zebrafish OR and cluster assignment for each zebrafish OR. [file 1471-2148-9-91-S2.pdf]

Legend

A	Number of A1 CPs
B	Number of A8 novel CPs
C	Number of Zebrafish novel CPs
D	Cluster number A1 novel CPs
E	Cluster number Zebrafish novel CPs
F	Niimura group
G	Alioto group

**Name**	**A**	**B**	**C**	**D**	**E**	**F**	**G**
Dr3OR5.4	34	0	1	1	-	γ	B
Dr3OR5.1	13	0	1	1	-	δ	F
Dr3OR15.43	17	0	2	1	-	ε	C
Dr3OR15.49	17	0	3	1	-	ε	C
Dr3OR15.50	9	0	4	1	-	ε	C
Dr3OR15.31	15	1	12	2	5	ζ	D
Dr3OR15.32	16	1	13	2	5	ζ	D
Dr3OR15.34	14	1	16	2	5	ζ	D
Dr3OR15.35	16	1	16	2	5	ζ	D
Dr3OR15.36	14	1	12	2	5	ζ	D
Dr3OR15.37	16	1	9	2	5	ζ	D
Dr3OR15.38	14	1	10	2	5	ζ	D
Dr3OR15.39	8	1	12	2	5	ζ	D
Dr3OR15.40	9	1	12	2	5	ζ	D
Dr3OR15.41	18	1	14	2	5	ζ	D
Dr3OR15.42	11	1	10	2	5	ζ	D
Dr3OR10.17	12	0	6	2	6	ζ	D
Dr3OR9.2	23	0	2	2	-	β	A
Dr3OR10.1	20	0	4	2	-	ε	C
Dr3OR15.30	11	1	1	2	-	ζ	D
Dr3OR21.12	10	1	6	3	3	δ	E
Dr3OR21.14	10	1	5	3	3	δ	E
Dr3OR21.15	11	1	5	3	3	δ	E
Dr3OR21.11	10	1	7	3	6	δ	E
Dr3OR21.6	12	1	1	3	-	δ	E
Dr3OR21.10	10	1	4	3	-	δ	E
Dr3OR10.4	11	0	6	4	1	ζ	G
Dr3OR10.5	10	0	8	4	1	ζ	G
Dr3OR10.8	11	0	6	4	1	ζ	G
Dr3OR10.11	12	0	7	4	1	ζ	G
Dr3OR10.12	12	0	8	4	1	ζ	G
Dr3OR10.14	12	0	6	4	1	ζ	G
Dr3OR10.15	12	0	8	4	1	ζ	G
Dr3OR15.44	13	0	3	5	-	ε	C
Dr3OR15.45	14	0	3	5	-	ε	C
Dr3OR15.46	17	0	3	5	-	ε	C
Dr3OR15.47	14	0	2	5	-	ε	C
Dr3OR15.48	13	0	3	5	-	ε	C
Dr3OR15.51	9	0	4	5	-	ε	C
Dr3OR15.53	8	0	4	5	-	ε	C
Dr3OR15.21	14	0	0	6	-	δ	F
Dr3OR15.22	11	0	0	6	-	δ	F
Dr3OR15.23	11	0	1	6	-	δ	F
Dr3OR15.24	13	0	0	6	-	δ	F
Dr3OR15.26	16	0	0	6	-	δ	F

Dr3OR15.27	14	0	0	6	-	δ	F
Dr3OR17.2	8	0	1	6	-	δ	E
Dr3OR21.3	11	0	0	6	-	δ	E
Dr3OR21.4	12	0	0	6	-	δ	E
Dr3OR21.5	14	0	0	6	-	δ	E
Dr3OR15.3	13	2	15	7	2	δ	E
Dr3OR15.4	15	1	16	7	2	δ	E
Dr3OR15.5	13	2	15	7	2	δ	E
Dr3OR15.6	18	1	13	7	2	δ	E
Dr3OR15.7	18	1	14	7	2	δ	E
Dr3OR15.8	10	3	9	7	2	δ	E
Dr3OR15.9	9	3	14	7	2	δ	E
Dr3OR15.10	8	3	12	7	2	δ	E
Dr3OR15.11	9	3	13	7	2	δ	E
Dr3OR15.17	16	1	7	7	2	δ	E
Dr3OR21.7	15	1	5	8	3	δ	E
Dr3OR21.8	15	1	5	8	3	δ	E
Dr3OR21.16	18	1	5	8	3	δ	E
Dr3OR15.1	18	1	2	8	-	δ	-
Dr3OR15.2	16	1	4	8	-	δ	E
Dr3OR15.12	15	3	2	8	-	δ	E
Dr3OR15.13	17	2	1	8	-	δ	E
Dr3OR15.14	16	2	3	8	-	δ	E
Dr3OR15.15	14	2	4	8	-	δ	E
Dr3OR15.16	15	4	2	8	-	δ	E
Dr3OR15.28	14	0	1	8	-	δ	F
Dr3OR15.29	14	0	1	8	-	δ	F
Dr3OR17.1	13	0	1	8	-	δ	E
Dr3OR10.16	9	0	5	9	5	ζ	D
Dr3OR10.18	10	2	6	9	6	ζ	D
Dr3OR10.19	11	0	6	9	6	ζ	D
Dr3OR10.20	10	0	6	9	6	ζ	D
Dr3OR10.24	10	0	5	9	6	ζ	D
Dr3OR10.21	8	0	4	9	-	ζ	D
Dr3OR10.23	9	1	4	9	-	ζ	D
Dr3OR8.5	5	1	5	10	4	η	H
Dr3OR21.19	5	1	9	10	4	η	H
Dr3OR21.21	5	1	7	10	4	η	-
Dr3OR21.22	5	1	8	10	4	η	H
Dr3OR21.24	6	1	10	10	4	η	H
Dr3OR21.25	7	1	8	10	4	η	H
Dr3OR21.26	5	1	8	10	4	η	H
Dr3OR21.28	5	1	5	10	4	η	H
Dr3OR8.4	5	1	4	10	-	η	H
Dr3OR8.6	5	1	4	10	-	η	H
Dr3OR7.3	4	0	2	-	-	η	H
Dr3OR7.4	3	0	3	-	-	η	H
Dr3OR8.2	1	0	1	-	-	η	H
Dr3OR15.18	4	1	1	-	-	η	H
Dr3OR15.20	4	1	1	-	-	η	H
Dr3OR2.1	2	0	0	-	-	θ	H
Dr3OR7.2	2	0	0	-	-	κ	-
