# Supplementary material for: Common peptides shed light on evolution of Olfactory Receptors
Source: BMC Evol Biol. 2009 May 5;9:91. doi: 10.1186/1471-2148-9-91 (PMC2681464; doi:10.1186/1471-2148-9-91)
Supplement: Additional file 3 — Frog ORs CP numbers and cluster assignment. Number of CPs from each ancestor occurring in each Frog OR and cluster assignment for each Frog OR. [file 1471-2148-9-91-S3.pdf]

Name	Number of CPs in A1	Number of novel CPs in A2	Number of frog novel CPs	Cluster number using CPs in A1	Cluster number using A2 novel CPs	Cluster number using frog novel CPs	Niimura group
Xt1OR5856.2	22	33	19	1	1	1	γ
Xt1OR9730.2	26	37	15	1	1	1	γ
Xt1OR24938.1	15	28	0	1	1	1	γ
Xt1OR32249.1	17	14	6	1	1	1	γ
Xt1OR55830.1	23	38	22	1	1	1	γ
Xt1OR5856.1	24	28	27	1	2	1	γ
Xt1OR5856.6	15	14	2	1	2	1	γ
Xt1OR7451.3	12	1	5	1	2	1	γ
Xt1OR27704.1	21	29	22	1	2	1	γ
Xt1OR28450.2	14	23	15	1	2	1	γ
Xt1OR34027.1	11	4	1	1	2	1	γ
Xt1OR40709.1	16	25	19	1	2	1	γ
Xt1OR41009.2	18	27	1	1	2	1	γ
Xt1OR41117.1	6	3	13	1	2	1	γ
Xt1OR27704.1	13	17	2	1	2	1	γ
Xt1OR7451.4	23	28	20	1	2	-	γ
Xt1OR7994.1	14	23	3	1	2	-	γ
Xt1OR3427.7	18	28	8	1	3	1	γ
Xt1OR7451.2	24	30	26	1	3	1	γ
Xt1OR8740.2	13	19	1	1	3	1	γ
Xt1OR8740.3	16	24	2	1	3	1	γ
Xt1OR10482.4	21	26	22	1	3	1	γ
Xt1OR14126.2	22	27	28	1	3	1	γ
Xt1OR3078.2	18	18	6	1	3	1	γ
Xt1OR3078.3	18	20	2	1	3	1	γ
Xt1OR7422.2	27	41	16	1	3	-	γ
Xt1OR3735.1	24	32	26	1	5	1	γ
Xt1OR5358.1	15	0	2	1	5	1	γ
Xt1OR5358.2	19	13	2	1	5	1	γ
Xt1OR5358.3	13	9	5	1	5	1	γ
Xt1OR7422.1	14	30	22	1	5	1	γ
Xt1OR8740.1	18	3	3	1	5	1	γ
Xt1OR9730.3	27	39	15	1	5	1	γ
Xt1OR9939.2	24	31	15	1	5	1	γ
Xt1OR9939.4	22	24	10	1	5	1	γ
Xt1OR10482.3	10	13	1	1	5	1	γ
Xt1OR14126.3	22	27	20	1	5	1	γ
Xt1OR17382.1	21	32	17	1	5	1	γ
Xt1OR17382.2	21	26	16	1	5	1	γ
Xt1OR20244.2	19	25	18	1	5	1	γ
Xt1OR20433.1	23	20	20	1	5	1	γ
Xt1OR22245.1	14	16	3	1	5	1	γ
Xt1OR23570.2	13	0	2	1	5	1	γ
Xt1OR41009.1	24	22	2	1	5	1	γ
Xt1OR51027.1	19	11	2	1	5	1	γ
Xt1OR52090.1	21	23	21	1	5	1	γ
Xt1OR56183.1	24	28	11	1	5	1	γ
Xt1OR56994.1	21	17	4	1	5	1	γ
Xt1OR64627.1	12	9	1	1	5	1	γ
Xt1OR65408.1	10	11	5	1	5	1	γ
Xt1OR23570.2	20	13	6	1	5	1	γ

Xt1OR25044.1	22	29	26	1	5	-	γ
Xt1OR9555.2	14	16	1	2	1	8	γ
Xt1OR9837.2	13	17	0	2	2	8	γ
Xt1OR35491.1	18	20	5	2	2	8	γ
Xt1OR58724.1	18	7	0	2	2	8	γ
Xt1OR68906.1	20	24	9	2	2	8	γ
Xt1OR9357.1	21	14	3	2	3	8	γ
Xt1OR9837.4	1	0	0	2	3	8	γ
Xt1OR20513.1	16	11	6	2	3	8	γ
Xt1OR22013.2	16	22	0	2	3	8	γ
Xt1OR22444.1	15	18	1	2	3	8	γ
Xt1OR25245.1	20	17	7	2	3	8	γ
Xt1OR26834.1	11	2	3	2	3	8	γ
Xt1OR28273.2	8	16	0	2	3	8	γ
Xt1OR36461.1	21	30	21	2	3	8	γ
Xt1OR36933.1	22	26	1	2	3	8	γ
Xt1OR37278.1	20	10	4	2	3	8	γ
Xt1OR41904.2	22	23	9	2	3	8	γ
Xt1OR42735.1	9	24	8	2	3	8	γ
Xt1OR17942.2	22	25	26	2	4	8	γ
Xt1OR17942.3	16	19	7	2	4	8	γ
Xt1OR3458.1	17	20	24	2	5	8	γ
Xt1OR6630.1	14	30	23	2	5	8	γ
Xt1OR8023.4	15	25	7	2	5	8	γ
Xt1OR8319.1	24	13	3	2	5	8	γ
Xt1OR8319.2	22	6	2	2	5	8	γ
Xt1OR11670.1	20	31	24	2	5	8	γ
Xt1OR11742.1	21	32	21	2	5	8	γ
Xt1OR11956.1	22	34	19	2	5	8	γ
Xt1OR11956.2	23	35	23	2	5	8	γ
Xt1OR12109.3	17	17	0	2	5	8	γ
Xt1OR12109.4	10	11	5	2	5	8	γ
Xt1OR14171.2	30	37	16	2	5	8	γ
Xt1OR14754.1	9	16	1	2	5	8	γ
Xt1OR14754.2	19	14	2	2	5	8	γ
Xt1OR14896.3	19	26	1	2	5	8	γ
Xt1OR16080.1	6	4	16	2	5	8	γ
Xt1OR16801.2	22	30	25	2	5	8	γ
Xt1OR17942.1	15	26	3	2	5	8	γ
Xt1OR19253.1	25	30	21	2	5	8	γ
Xt1OR19799.2	12	13	3	2	5	8	γ
Xt1OR22940.1	18	17	19	2	5	8	γ
Xt1OR28345.3	17	11	1	2	5	8	γ
Xt1OR29560.1	12	0	2	2	5	8	γ
Xt1OR29875.1	15	28	1	2	5	8	γ
Xt1OR29919.1	15	18	3	2	5	8	γ
Xt1OR35885.2	11	11	0	2	5	8	γ
Xt1OR36854.1	11	21	1	2	5	8	γ
Xt1OR37065.2	21	23	26	2	5	8	γ
Xt1OR53932.1	22	29	23	2	5	8	γ
Xt1OR57187.1	22	29	29	2	5	8	γ
Xt1OR3389.1	20	26	22	3	1	3	γ
Xt1OR9064.2	20	25	10	3	1	3	γ
Xt1OR22749.1	24	13	4	3	1	3	γ
Xt1OR18276.2	12	23	1	3	1	9	γ

Xt1OR22277.1	19	17	6	3	1	9	γ
Xt1OR25568.1	17	34	18	3	1	9	γ
Xt1OR18276.2	16	27	2	3	1	9	γ
Xt1OR22277.1	27	40	16	3	1	9	γ
Xt1OR3374.1	21	29	22	3	2	3	γ
Xt1OR3374.2	16	14	4	3	2	3	γ
Xt1OR3374.4	21	29	22	3	2	3	γ
Xt1OR21725.1	18	18	12	3	2	3	γ
Xt1OR27101.1	24	33	19	3	2	3	γ
Xt1OR27101.2	18	17	10	3	2	3	γ
Xt1OR6349.1	15	27	19	3	2	9	γ
Xt1OR9439.1	17	13	3	3	2	9	γ
Xt1OR11826.2	18	16	2	3	2	9	γ
Xt1OR12839.1	11	0	3	3	2	9	γ
Xt1OR13656.1	27	30	16	3	2	9	γ
Xt1OR17800.1	16	23	4	3	2	9	γ
Xt1OR27472.2	22	20	27	3	2	9	γ
Xt1OR17800.1	17	21	6	3	2	9	γ
Xt1OR30229.2	12	34	2	3	3	3	γ
Xt1OR43871.1	19	20	1	3	3	3	γ
Xt1OR62303.1	13	10	0	3	3	3	γ
Xt1OR14807.2	15	22	5	3	3	9	γ
Xt1OR17800.3	13	25	1	3	3	9	γ
Xt1OR12245.1	14	22	7	3	5	3	γ
Xt1OR15876.1	7	3	17	3	5	3	γ
Xt1OR16668.1	22	25	23	3	5	3	γ
Xt1OR23535.1	22	29	28	3	5	3	γ
Xt1OR23535.2	21	30	27	3	5	3	γ
Xt1OR39273.1	21	27	25	3	5	3	γ
Xt1OR3428.3	16	19	0	3	5	9	γ
Xt1OR9954.1	13	15	1	3	5	9	γ
Xt1OR14807.3	23	37	20	3	5	9	γ
Xt1OR15603.1	18	11	2	3	5	9	γ
Xt1OR15603.2	11	26	4	3	5	9	γ
Xt1OR15603.3	10	1	5	3	5	9	γ
Xt1OR22277.2	17	28	16	3	5	9	γ
Xt1OR22490.2	24	30	22	3	5	9	γ
Xt1OR29421.1	15	27	23	3	5	9	γ
Xt1OR29947.1	7	4	15	3	5	9	γ
Xt1OR30196.1	17	20	0	3	5	9	γ
Xt1OR31127.1	23	27	25	3	5	9	γ
Xt1OR34945.1	16	27	21	3	5	9	γ
Xt1OR35901.1	14	3	2	3	5	9	γ
Xt1OR39494.1	21	25	24	3	5	9	γ
Xt1OR49233.1	23	30	24	3	5	9	γ
Xt1OR52587.1	15	23	4	3	5	9	γ
Xt1OR15603.2	16	17	8	3	5	9	γ
Xt1OR15603.3	21	32	13	3	5	9	γ
Xt1OR3428.3	22	25	22	3	5	9	γ
Xt1OR16390.1	22	20	29	3	5	-	γ
Xt1OR34566.1	18	15	4	3	5	-	γ
Xt1OR3116.2	18	20	7	4	1	3	γ
Xt1OR3116.3	11	20	2	4	1	3	γ
Xt1OR9937.1	28	33	18	4	1	3	γ
Xt1OR26158.1	21	19	7	4	1	3	γ

Xt1OR17535.3	16	27	22	4	1	6	γ
Xt1OR22035.1	14	27	18	4	1	7	γ
Xt1OR42847.2	19	16	2	4	1	7	γ
Xt1OR5339.2	14	13	2	4	1	-	γ
Xt1OR9220.2	20	12	3	4	1	-	γ
Xt1OR14061.1	25	31	15	4	1	-	γ
Xt1OR20565.1	24	30	23	4	1	-	γ
Xt1OR22554.1	13	13	3	4	1	-	γ
Xt1OR25964.1	16	29	7	4	1	-	γ
Xt1OR29482.2	15	13	4	4	1	-	γ
Xt1OR45359.1	24	27	24	4	1	-	γ
Xt1OR25964.1	14	3	4	4	1	-	γ
Xt1OR4811.2	17	32	0	4	2	3	γ
Xt1OR19403.1	14	2	4	4	2	3	γ
Xt1OR31520.1	13	28	2	4	2	3	γ
Xt1OR62426.1	18	18	6	4	2	3	γ
Xt1OR21356.1	21	20	25	4	2	4	γ
Xt1OR33320.1	19	19	1	4	2	4	γ
Xt1OR18658.3	16	19	2	4	2	8	γ
Xt1OR55629.1	20	38	19	4	2	8	γ
Xt1OR51098.1	15	0	2	4	2	9	γ
Xt1OR3833.2	15	12	4	4	2	-	γ
Xt1OR5498.1	18	18	3	4	2	-	γ
Xt1OR9560.4	26	34	15	4	2	-	γ
Xt1OR10601.3	21	32	7	4	2	-	γ
Xt1OR13207.1	18	24	22	4	2	-	γ
Xt1OR15862.1	8	5	9	4	2	-	γ
Xt1OR19366.1	25	35	15	4	2	-	γ
Xt1OR21066.1	15	8	4	4	2	-	γ
Xt1OR25044.3	16	10	0	4	2	-	γ
Xt1OR26074.1	18	24	9	4	2	-	γ
Xt1OR26074.2	19	21	0	4	2	-	γ
Xt1OR26878.2	24	31	26	4	2	-	γ
Xt1OR27812.1	24	25	22	4	2	-	γ
Xt1OR45488.1	20	20	27	4	2	-	γ
Xt1OR50407.1	20	17	1	4	2	-	γ
Xt1OR29757.2	6	3	16	4	3	7	γ
Xt1OR16474.1	24	29	20	4	3	8	γ
Xt1OR18612.1	17	14	5	4	3	8	γ
Xt1OR9064.1	18	26	6	4	3	9	γ
Xt1OR47891.1	23	31	23	4	3	9	γ
Xt1OR5151.1	18	25	3	4	3	-	γ
Xt1OR5339.4	13	3	0	4	3	-	γ
Xt1OR11138.2	17	23	6	4	3	-	γ
Xt1OR12702.2	11	3	1	4	3	-	γ
Xt1OR17771.1	15	24	4	4	3	-	γ
Xt1OR19783.1	24	31	27	4	3	-	γ
Xt1OR23566.1	23	26	25	4	3	-	γ
Xt1OR25146.1	18	17	0	4	3	-	γ
Xt1OR30036.2	7	4	14	4	3	-	γ
Xt1OR30827.1	13	15	3	4	3	-	γ
Xt1OR33380.1	18	21	7	4	3	-	γ
Xt1OR37563.1	18	29	9	4	3	-	γ
Xt1OR45335.1	17	3	3	4	3	-	γ
Xt1OR49135.1	13	22	8	4	3	-	γ

Xt1OR49836.1	3	0	0	4	3	-	γ
Xt1OR50263.1	22	27	28	4	3	-	γ
Xt1OR61281.1	19	27	26	4	3	-	γ
Xt1OR45335.1	18	24	7	4	3	-	γ
Xt1OR61281.1	20	11	0	4	3	-	γ
Xt1OR47529.2	15	15	6	4	4	8	γ
Xt1OR3795.1	16	27	14	4	4	-	γ
Xt1OR3833.1	15	11	5	4	4	-	γ
Xt1OR5104.2	15	23	3	4	4	-	γ
Xt1OR33122.1	10	11	3	4	4	-	γ
Xt1OR41875.2	20	19	6	4	4	-	γ
Xt1OR47982.1	16	28	5	4	4	-	γ
Xt1OR41875.2	16	11	3	4	4	-	γ
Xt1OR47982.1	16	18	1	4	4	-	γ
Xt1OR3795.2	15	10	5	4	5	2	γ
Xt1OR13035.1	13	22	1	4	5	2	γ
Xt1OR25788.1	22	33	21	4	5	2	γ
Xt1OR4811.3	20	23	17	4	5	3	γ
Xt1OR4811.4	23	29	26	4	5	3	γ
Xt1OR15006.1	11	19	0	4	5	3	γ
Xt1OR15478.1	17	10	3	4	5	3	γ
Xt1OR21320.1	16	12	6	4	5	3	γ
Xt1OR22135.1	25	28	27	4	5	3	γ
Xt1OR22644.2	21	33	11	4	5	3	γ
Xt1OR25295.1	23	37	20	4	5	3	γ
Xt1OR31304.1	19	27	9	4	5	3	γ
Xt1OR34718.1	17	19	3	4	5	3	γ
Xt1OR36836.1	27	31	17	4	5	3	γ
Xt1OR43677.1	11	4	4	4	5	3	γ
Xt1OR44345.1	19	30	20	4	5	3	γ
Xt1OR20595.1	16	20	5	4	5	4	γ
Xt1OR20595.2	16	9	4	4	5	4	γ
Xt1OR42256.1	15	20	5	4	5	4	γ
Xt1OR29545.1	21	35	13	4	5	6	γ
Xt1OR12007.1	28	33	20	4	5	7	γ
Xt1OR13598.1	10	11	0	4	5	7	γ
Xt1OR32100.1	23	21	25	4	5	7	γ
Xt1OR36311.2	24	25	15	4	5	7	γ
Xt1OR41326.1	22	21	25	4	5	7	γ
Xt1OR50884.2	13	16	2	4	5	7	γ
Xt1OR60647.1	19	16	6	4	5	7	γ
Xt1OR14618.1	15	19	1	4	5	8	γ
Xt1OR14841.1	25	35	20	4	5	8	γ
Xt1OR20177.1	23	28	21	4	5	8	γ
Xt1OR22779.1	18	26	17	4	5	8	γ
Xt1OR33154.1	13	10	1	4	5	8	γ
Xt1OR42619.1	15	15	2	4	5	8	γ
Xt1OR10411.1	12	15	1	4	5	9	γ
Xt1OR43378.1	24	33	23	4	5	9	γ
Xt1OR3755.1	24	38	17	4	5	-	γ
Xt1OR4379.1	16	20	22	4	5	-	γ
Xt1OR4379.2	20	28	23	4	5	-	γ
Xt1OR4571.2	13	21	4	4	5	-	γ
Xt1OR5339.1	14	21	18	4	5	-	γ
Xt1OR5339.3	21	29	3	4	5	-	γ

Xt1OR5992.1	19	23	6	4	5	-	γ
Xt1OR6270.1	14	16	0	4	5	-	γ
Xt1OR6343.1	18	14	7	4	5	-	γ
Xt1OR6343.3	16	11	6	4	5	-	γ
Xt1OR6697.1	13	26	21	4	5	-	γ
Xt1OR7451.1	26	22	9	4	5	-	γ
Xt1OR7764.1	12	14	1	4	5	-	γ
Xt1OR8327.2	17	3	4	4	5	-	γ
Xt1OR9040.2	16	24	0	4	5	-	γ
Xt1OR10073.2	8	4	2	4	5	-	γ
Xt1OR10073.3	12	0	2	4	5	-	γ
Xt1OR10336.2	15	21	2	4	5	-	γ
Xt1OR10528.1	21	33	23	4	5	-	γ
Xt1OR10601.1	11	13	1	4	5	-	γ
Xt1OR10609.1	18	23	4	4	5	-	γ
Xt1OR11058.2	14	16	3	4	5	-	γ
Xt1OR11058.4	18	23	5	4	5	-	γ
Xt1OR12356.2	11	0	1	4	5	-	γ
Xt1OR12702.1	9	1	1	4	5	-	γ
Xt1OR12702.3	12	1	1	4	5	-	γ
Xt1OR13035.2	18	30	23	4	5	-	γ
Xt1OR13241.1	20	30	19	4	5	-	γ
Xt1OR14239.2	27	28	15	4	5	-	γ
Xt1OR14549.2	20	23	28	4	5	-	γ
Xt1OR15020.1	13	9	2	4	5	-	γ
Xt1OR15020.2	18	20	2	4	5	-	γ
Xt1OR16736.1	21	28	27	4	5	-	γ
Xt1OR17001.1	16	18	7	4	5	-	γ
Xt1OR18602.2	17	25	1	4	5	-	γ
Xt1OR18653.1	22	25	23	4	5	-	γ
Xt1OR19892.1	14	21	1	4	5	-	γ
Xt1OR19892.2	19	13	6	4	5	-	γ
Xt1OR20350.1	18	17	15	4	5	-	γ
Xt1OR22739.1	25	34	18	4	5	-	γ
Xt1OR22739.2	17	28	9	4	5	-	γ
Xt1OR24353.1	20	18	6	4	5	-	γ
Xt1OR24374.1	15	26	2	4	5	-	γ
Xt1OR24468.1	20	22	1	4	5	-	γ
Xt1OR24775.2	22	21	11	4	5	-	γ
Xt1OR25354.1	12	6	1	4	5	-	γ
Xt1OR25888.1	12	10	1	4	5	-	γ
Xt1OR26205.2	16	29	7	4	5	-	γ
Xt1OR26941.1	15	19	6	4	5	-	γ
Xt1OR26941.2	17	24	10	4	5	-	γ
Xt1OR27832.2	16	13	0	4	5	-	γ
Xt1OR28491.2	17	11	5	4	5	-	γ
Xt1OR28977.2	23	30	22	4	5	-	γ
Xt1OR29026.1	15	27	23	4	5	-	γ
Xt1OR30827.2	13	15	3	4	5	-	γ
Xt1OR32907.1	14	9	0	4	5	-	γ
Xt1OR34203.1	20	29	23	4	5	-	γ
Xt1OR34772.1	10	21	21	4	5	-	γ
Xt1OR37736.1	24	30	26	4	5	-	γ
Xt1OR37794.2	14	15	5	4	5	-	γ
Xt1OR39782.1	19	12	1	4	5	-	γ

Xt1OR42561.1	25	35	18	4	5	-	γ
Xt1OR42648.1	18	12	2	4	5	-	γ
Xt1OR42821.1	18	26	10	4	5	-	α
Xt1OR43447.1	18	25	2	4	5	-	γ
Xt1OR43748.1	17	17	3	4	5	-	γ
Xt1OR43764.1	19	24	9	4	5	-	γ
Xt1OR43932.1	20	17	23	4	5	-	γ
Xt1OR43932.2	9	0	2	4	5	-	γ
Xt1OR49957.1	23	26	21	4	5	-	γ
Xt1OR51597.1	17	31	25	4	5	-	γ
Xt1OR55135.1	9	1	14	4	5	-	γ
Xt1OR56385.1	15	18	7	4	5	-	γ
Xt1OR58146.1	18	27	7	4	5	-	γ
Xt1OR60944.1	13	11	2	4	5	-	γ
Xt1OR72086.1	18	28	4	4	5	-	γ
Xt1OR30827.2	12	21	2	4	5	-	γ
Xt1OR37794.2	15	16	0	4	5	-	γ
Xt1OR49957.1	9	14	1	4	5	-	γ
Xt1OR27832.2	20	19	28	4	5	-	γ
Xt1OR42821.1	18	14	1	4	5	-	α
Xt1OR43932.1	11	23	3	4	5	-	γ
Xt1OR55135.1	18	24	1	4	5	-	γ
Xt1OR42561.1	24	30	24	4	5	-	γ
Xt1OR72086.1	16	30	24	4	5	-	γ
Xt1OR6017.2	20	23	6	4	-	-	δ
Xt1OR6017.3	20	17	6	4	-	-	δ
Xt1OR9094.3	18	16	0	4	-	-	δ
Xt1OR9183.2	12	17	2	4	-	-	δ
Xt1OR9183.3	1	0	0	4	-	-	δ
Xt1OR9183.4	4	0	0	4	-	-	δ
Xt1OR14152.1	17	28	10	4	-	-	ε
Xt1OR18223.2	15	23	3	4	-	-	β
Xt1OR23947.1	24	25	19	4	-	-	β
Xt1OR26423.1	14	31	22	4	-	-	γ
Xt1OR34498.1	19	36	1	4	-	-	γ
Xt1OR34965.1	20	19	27	4	-	-	β
Xt1OR43348.1	12	6	1	4	-	-	α
Xt1OR34498.1	15	27	22	4	-	-	γ
Xt1OR6017.2	16	25	0	4	-	-	δ
Xt1OR2286.2	11	12	0	4	-	-	β
Xt1OR6017.3	16	15	6	4	-	-	δ
Xt1OR9094.3	26	30	7	4	-	-	δ
Xt1OR9183.2	17	17	1	4	-	-	δ
Xt1OR9183.3	24	31	17	4	-	-	δ
Xt1OR9183.4	21	30	25	4	-	-	δ
Xt1OR18223.2	22	28	28	4	-	-	β
Xt1OR23947.1	12	14	2	4	-	-	β
Xt1OR34965.1	19	29	0	4	-	-	β
Xt1OR34006.1	18	30	0	5	1	-	δ
Xt1OR11576.2	16	23	0	5	5	5	δ
Xt1OR3578.1	25	28	20	5	-	5	δ
Xt1OR11576.1	15	24	4	5	-	5	δ
Xt1OR11576.3	15	14	0	5	-	5	δ
Xt1OR11576.4	21	30	24	5	-	5	δ
Xt1OR23967.1	12	15	0	5	-	5	δ

Xt1OR24457.1	20	14	5	5	-	5	δ
Xt1OR24457.2	21	27	22	5	-	5	δ
Xt1OR30930.2	16	22	2	5	-	5	δ
Xt1OR40750.1	15	1	1	5	-	5	δ
Xt1OR11576.1	15	5	1	5	-	5	δ
Xt1OR4132.1	23	26	26	5	-	-	ε
Xt1OR4132.2	25	14	1	5	-	-	ε
Xt1OR9094.2	21	25	11	5	-	-	δ
Xt1OR21814.2	18	15	11	5	-	-	δ
Xt1OR27949.2	20	27	11	5	-	-	ε
Xt1OR30788.1	25	33	20	5	-	-	δ
Xt1OR35679.1	15	16	3	5	-	-	ε
Xt1OR39249.1	21	28	23	5	-	-	δ
Xt1OR53675.1	3	1	0	5	-	-	ε
Xt1OR9094.2	24	36	27	5	-	-	δ
Xt1OR21814.2	14	19	7	5	-	-	δ
Xt1OR65880.1	15	20	3	-	-	5	δ
Xt1OR65880.1	13	20	7	-	-	5	δ
Xt1OR5329.1	16	28	8	-	-	-	η
Xt1OR5329.2	15	23	6	-	-	-	η
Xt1OR5508.1	18	18	0	-	-	-	κ
Xt1OR36957.1	14	18	2	-	-	-	θ
Xt1OR40237.1	20	20	23	-	-	-	η
Xt1OR5329.1	28	35	17	-	-	-	η
Xt1OR5329.2	23	38	10	-	-	-	η
Xt1OR5508.1	3	3	16	-	-	-	κ
Xt1OR36957.1	22	30	25	-	-	-	θ
Xt1OR40237.1	14	12	4	-	-	-	η
