# Supplementary material for: Common peptides shed light on evolution of Olfactory Receptors
Source: BMC Evol Biol. 2009 May 5;9:91. doi: 10.1186/1471-2148-9-91 (PMC2681464; doi:10.1186/1471-2148-9-91)
Supplement: Additional file 4 — Chicken ORs CP numbers and cluster assignment. Number of CPs from each ancestor occurring in each Chicken OR and cluster assignment for each Chicken OR. [file 1471-2148-9-91-S4.pdf]

Legend

A	Number of A1 CPs
B	Number of A2 novel CPs
C	Number of A3 novel CPs
D	Number of A7 novel CPs
E	Number of chicken novel CPs
F	Cluster number A1 CPs
G	Cluster number A2 novel CPs
H	Cluster number A3 novel CPs
I	Niimura group

**Name**	**A**	**B**	**C**	**D**	**E**	**F**	**G**	**H**	**I**
Gg2OR1.2	17	10	18	0	2	1	1	-	α
Gg2OR1.3	15	11	17	0	2	1	1	-	α
Gg2OR1.7	11	8	10	0	1	1	1	-	α
Gg2OR1.8	7	10	9	0	1	1	1	-	α
Gg2OR1.1[illegible]	13	7	5	0	1	1	1	-	α
Gg2ORUn.[illegible]	10	9	11	0	2	1	1	-	α
Gg2OR1.9	13	5	4	0	0	1	2	-	α
Gg2OR1.1[illegible]	16	7	10	0	4	1	2	-	α
Gg2OR1.6	12	4	5	0	1	1	-	-	α
Gg2ORUn.[illegible]	19	15	6	4	8	2	2	1	γ
Gg2ORUn.[illegible]	24	13	5	2	9	2	2	1	γ
Gg2ORUn.[illegible]	24	15	5	2	12	2	2	1	γ
Gg2ORUn.[illegible]	25	16	4	2	14	2	2	1	γ
Gg2ORUn.[illegible]	23	20	7	0	12	2	2	1	γ
Gg2ORUn.[illegible]	24	17	4	2	13	2	2	1	γ
Gg2ORUn.[illegible]	22	14	4	3	6	2	2	1	γ
Gg2ORUn.[illegible]	24	13	5	2	8	2	2	1	γ
Gg2ORUn.[illegible]	19	14	5	5	11	2	2	1	γ
Gg2ORUn.[illegible]	21	15	4	1	12	2	2	1	γ
Gg2ORUn.[illegible]	21	17	3	3	8	2	2	1	γ
Gg2ORUn.[illegible]	24	17	4	3	13	2	2	1	γ
Gg2ORUn.[illegible]	22	16	3	0	9	2	2	1	γ
Gg2ORUn.[illegible]	20	17	4	2	15	2	2	1	γ
Gg2ORUn.[illegible]	23	15	5	2	11	2	2	1	γ
Gg2ORUn.[illegible]	23	13	4	4	9	2	2	1	γ
Gg2ORUn.[illegible]	23	11	6	3	8	2	2	1	γ
Gg2ORUn.[illegible]	20	17	5	2	11	2	2	1	γ
Gg2ORUn.[illegible]	21	11	4	0	14	2	2	1	γ
Gg2ORUn.[illegible]	22	12	4	3	10	2	2	1	γ
Gg2ORUn.[illegible]	23	11	6	3	8	2	2	1	γ
Gg2ORUn.[illegible]	22	16	4	3	9	2	2	1	γ
Gg2ORUn.[illegible]	23	18	4	3	15	2	2	1	γ
Gg2ORUn.[illegible]	23	17	5	3	12	2	2	1	γ
Gg2ORUn.[illegible]	22	14	5	3	8	2	2	1	γ
Gg2ORUn.[illegible]	21	10	5	3	9	2	2	1	γ
Gg2ORUn.[illegible]	22	16	5	2	10	2	2	1	γ
Gg2ORUn.[illegible]	21	16	5	2	10	2	2	1	γ
Gg2ORUn.[illegible]	23	19	4	3	13	2	2	1	γ
Gg2ORUn.[illegible]	24	16	8	3	11	2	2	1	γ
Gg2ORUn.[illegible]	20	21	4	0	10	2	2	1	γ

Gg2ORUn.[illegible]	23	11	5	2	7	2	2	1	γ
Gg2ORUn.[illegible]	22	18	4	2	11	2	2	1	γ
Gg2ORUn.[illegible]	19	14	7	2	7	2	2	1	γ
Gg2ORUn.[illegible]	23	16	3	2	12	2	2	1	γ
Gg2ORUn.[illegible]	23	13	4	2	14	2	2	1	γ
Gg2ORUn.[illegible]	22	20	8	0	7	2	2	2	γ
Gg2ORUn.[illegible]	24	15	4	3	7	2	2	2	γ
Gg2ORUn.[illegible]	23	18	3	1	7	2	2	2	γ
Gg2OR27.[illegible]	11	25	3	0	0	2	3	-	γ
Gg2ORUn.[illegible]	23	18	4	2	11	2	-	1	γ
Gg2OR1.1	12	18	2	0	0	3	3	-	γ
Gg2OR5.2	18	14	1	0	1	3	3	-	γ
Gg2OR5.3	16	22	7	0	2	3	3	-	γ
Gg2OR5.4	22	31	4	0	2	3	3	-	γ
Gg2OR5.5	25	36	11	0	0	3	3	-	γ
Gg2OR5.7	27	20	2	0	1	3	3	-	γ
Gg2OR5.8	18	26	6	0	1	3	3	-	γ
Gg2OR5.9	15	20	4	0	5	3	3	-	γ
Gg2OR5.1[illegible]	16	19	9	0	0	3	3	-	γ
Gg2OR5.1[illegible]	22	26	6	0	0	3	3	-	γ
Gg2OR5.1[illegible]	16	19	8	0	5	3	3	-	γ
Gg2OR5.1[illegible]	16	22	8	0	5	3	3	-	γ
Gg2OR5.1[illegible]	24	19	3	0	0	3	3	-	γ
Gg2OR5.1[illegible]	15	19	7	0	6	3	3	-	γ
Gg2OR5.1[illegible]	21	19	3	0	0	3	3	-	γ
Gg2OR10.[illegible]	13	18	1	0	1	3	3	-	γ
Gg2OR10.[illegible]	20	21	2	0	0	3	3	-	γ
Gg2OR10.[illegible]	18	22	0	0	2	3	3	-	γ
Gg2OR10.[illegible]	15	21	1	0	0	3	3	-	γ
Gg2OR17.[illegible]	19	27	0	0	1	3	3	-	γ
Gg2OR27.[illegible]	18	7	1	0	1	3	3	-	γ
Gg2ORUn.[illegible]	17	14	2	0	2	3	3	-	γ
Gg2ORUn.[illegible]	16	13	0	0	1	3	3	-	γ
Gg2ORUn.[illegible]	19	25	2	0	0	3	3	-	γ
Gg2ORUn.[illegible]	22	17	5	0	1	3	3	-	γ
Gg2ORUn.[illegible]	20	20	9	0	0	3	3	-	γ
Gg2OR10[illegible]	9	16	0	0	1	3	3	-	γ
Gg2OR10[illegible]	12	21	1	0	0	3	3	-	γ
