# Supplementary material for: Common peptides shed light on evolution of Olfactory Receptors
Source: BMC Evol Biol. 2009 May 5;9:91. doi: 10.1186/1471-2148-9-91 (PMC2681464; doi:10.1186/1471-2148-9-91)
Supplement: Additional file 5 — Zebrafish CPs locations and cluster assignment. Zebrafish CPs with sequence locations and cluster assignments. [file 1471-2148-9-91-S5.pdf]

ancestral (A1) CPs clusters

cluster #	CP	domain
1	AICNP	IL2
1	ICNPL	IL2
1	NPLRY	IL2
2	AICLPL	IL2
2	ERSLH	IL1
2	FFVFL	TM2
2	IIFSY	TM5
2	KALKT	TM6
2	LACND	EL2
2	LLIIA	EL1
2	LLLVG	EL2
2	LNPII	TM7
2	NPIIY	TM7
2	PLRYH	IL2
2	PMLNP	TM7
2	RLSFC	EL2
2	SLHSP	EL1
2	TCVSH	TM6
3	FLCNL	TM2
3	ICQPL	IL2
3	NNIYG	EL2
3	NPVIY	TM7
3	PMYIF	IL1
3	YCDNWS	EL2
4	DRLIAI	IL2
4	ILLGN	TM1
4	LLGNG	TM1
4	MAIDR	TM3
4	PLINP	TM7
4	RTKEI	TM7
4	TKEIR	C
5	CFVQMFF	TM3
5	GFPGL	N
5	ICYPL	IL2
5	ISFLG	EL1
5	KTFSTC	IL3
5	LAVMA	TM3
5	QMFFVH	TM3
6	DRYIA	IL2
6	KALQTC	IL3
6	LHKPM	IL1
6	RYIAI	IL2
6	TCGTHL	TM6
6	YIAIC	IL2
7	AICKP	IL2
7	DLLSE	TM3
7	DRFVA	IL2
7	ICKPL	IL2
7	ILIIC	EL2
7	LHQPM	IL1
7	MAFDR	TM3
7	MALDR	TM3
7	NPLVY	TM7

zebrafish novel CPs clusters

cluster #	CP	domain
1	CTYAVLAYSVTKL	IL3
1	IKYVFC	EL2
1	ITGFDHLQNQKLLG	N
1	LAVVDI	TM2
1	LTLMA	TM3
1	NPTVYC	TM7
1	NSSRKQMINTCLSH	IL3
1	VSYYSCI	EL1
2	CGNKLWKV	EL2
2	IIPLSLI	TM5
2	ILMLMA	TM3
2	INNAFGL	EL2
2	IRYALF	TM1
2	ISREAC	EL1
2	KLSCDNI	EL2
2	KVYCH	EL2
2	LSVFL	TM5
2	MCGNKL	EL2
2	MLFEN	N
2	PRFLA	TM3
2	SIVRP	TM3
2	TLSRV	TM6
2	VGLSVF	EL3
2	YILIS	TM2
3	SYIKL	TM5
4	GVIQE	TM6
4	IFTYA	EL2
4	LFCSRTTL	EL2
4	LHLIQLGL	IL3
4	QEVIYV	TM6
4	SAKKA	IL3
4	TLRKK	TM2
4	VTPCV	TM2
5	ACLAN	EL1
5	CDHGP	EL2
5	EGRLK	IL3
5	FNLALAD	TM2
5	GETNA	TM2
5	GLSGI	N
5	KITTWE	IL3
5	LALADI	TM2
5	MVSLI	EL2
5	NMFFV	TM3
5	PEYFF	N
5	QSFTLV	TM3
5	RLKAL	IL3
5	SQYISYN	EL1
5	YYYIFL	TM1
6	DRLVAIC	IL2
6	QSLTL	TM3

7	PMYIL	IL1
7	PVLNP	TM7
7	SLEFL	TM6
7	SYVKI	TM5
7	TCIPH	TM6
8	AICRPL	IL2
8	DRYVA	IL2
8	LHEPM	IL1
8	LNPLI	TM7
8	LTVMA	TM3
8	MAYDR	TM3
8	MSYDR	TM3
8	NNVFG	EL2
8	NPLIY	TM7
8	RYVAI	IL2
8	TCLPH	TM6
8	VMAYD	TM3
8	YVAIC	IL2
9	TCTSH	TM6
9	TVLGN	TM1
10	ICFPL	IL2
10	LYVNG	TM2
10	PLIYG	TM7
