# Supplementary material for: Common peptides shed light on evolution of Olfactory Receptors
Source: BMC Evol Biol. 2009 May 5;9:91. doi: 10.1186/1471-2148-9-91 (PMC2681464; doi:10.1186/1471-2148-9-91)
Supplement: Additional file 6 — Opossum ORs CP numbers and cluster assignment. Number of CPs from each ancestor occurring in each Opossum OR and cluster assignment for each Opossum OR. [file 1471-2148-9-91-S6.pdf]

Legend

A	Number of A1 CPs
B	Number of A2 novel CPs
C	Number of A3 novel CPs
D	Number of A4 novel CPs
E	Number of A5 novel CPs
F	Number of opossum novel CPs
G	Cluster number A4 novel CPs
H	Cluster number A5 novel CPs
I	Family

Name	A	B	C	D	E	F	G	H	I
opp_cand.172-2G	15	22	2	2	5	1	1	8	2
opp_cand.82-2F	21	17	2	2	2	1	1	-	2
opp_cand.83-2F	22	20	2	1	2	1	1	-	2
opp_cand_r2.97-2F	22	22	2	1	2	1	1	-	2
opp_cand.280-2BE	16	25	0	3	2	0	2	-	2
opp_cand_r2.143-2BE	19	15	2	4	0	0	2	-	2
opp_cand_r2.44-2C	22	28	4	4	3	0	2	-	2
opp_cand.121-2D	21	21	2	4	3	1	2	-	2
opp_cand.42-2D	21	23	1	2	4	2	2	-	2
opp_cand_r5.454-2D	22	21	2	1	2	2	2	-	2
opp_cand.118-5B	14	35	4	2	10	0	3	5	5
opp_cand_r2.162-5B	18	45	7	2	11	0	3	5	5
opp_cand_r2.50-5B	15	40	8	1	5	2	3	5	5
opp_cand_r2.8-5B	15	42	5	4	9	2	3	5	5
opp_cand_r3.10-5B	15	36	3	1	6	4	3	5	5
opp_cand_r3.11-5B	16	19	3	4	5	0	3	5	5
opp_cand_r3.14-5B	11	38	9	4	7	1	3	5	5
opp_cand_r3.15-5B	16	32	2	1	5	3	3	5	5
opp_cand_r3.16-5B	14	33	4	3	7	1	3	5	5
opp_cand_r3.24-5B	17	31	5	2	5	4	3	-	5
opp_cand.67-5AC	18	16	1	2	6	1	4	3	5
opp_cand_r3.71-4L	23	16	3	1	8	2	4	4	4
opp_cand_r3.72-4L	25	15	1	3	6	2	4	4	4
opp_cand_r5.24-4L	25	14	1	1	8	2	4	4	4
opp_cand_r5.25-4L	21	15	10	5	5	1	4	4	4
opp_cand.110-5B	13	37	6	2	10	1	4	5	5
opp_cand.15-4G	13	10	1	3	3	0	4	-	4
opp_cand_r5.13-4G	12	14	2	5	3	1	4	-	4
opp_cand.608-4H	18	16	3	2	2	0	4	-	4
opp_cand.22-4K	18	17	7	2	1	1	4	-	4
opp_cand.29-4K	22	17	7	4	2	0	4	-	4
opp_cand.30-4K	24	23	6	3	3	0	4	-	4
opp_cand.31-4K	20	19	3	2	3	0	4	-	4
opp_cand_r2.116-4K	21	23	3	2	2	0	4	-	4
opp_cand_r2.145-4K	20	14	4	3	3	0	4	-	4
opp_cand_r2.161-4K	19	16	4	2	4	0	4	-	4
opp_cand_r5.18-4K	17	17	8	2	3	0	4	-	4
opp_cand_r5.28-4K	16	13	5	3	2	1	4	-	4
opp_cand_r3.69-4L	19	15	5	3	4	0	4	-	4
opp_cand.469-4P	19	12	5	4	3	0	4	-	4
opp_cand.606-4Q	21	23	7	0	0	0	4	-	4
opp_cand_r5.26-4Q	22	20	9	3	0	1	4	-	4
opp_cand_r5.27-4Q	18	11	7	3	3	0	4	-	4
opp_cand.150-4S	23	26	4	4	1	0	4	-	4

opp_cand_r3.77-4S	22	22	2	4	0	1	4	-	4
opp_cand_r3.79-4S	28	27	7	2	0	0	4	-	4
opp_cand.364-5AQ	16	33	6	1	1	2	4	-	5
opp_cand_r3.212-5AX	18	26	5	3	0	0	4	-	5
opp_cand_r2.223-1AB	26	27	4	5	5	0	5	3	1
opp_cand.201-4F	18	13	5	3	5	0	5	4	4
opp_cand.21-4F	14	13	7	9	5	0	5	4	4
opp_cand_r2.84-4F	22	14	6	3	5	1	5	4	4
opp_cand_r3.121-4F	20	15	7	4	7	0	5	4	4
opp_cand_r3.122-4F	17	13	6	5	6	0	5	4	4
opp_cand_r3.123-4F	18	16	6	4	7	1	5	4	4
opp_cand_r5.115-4F	17	15	6	5	7	0	5	4	4
opp_cand_r5.12-4F	17	11	6	6	6	1	5	4	4
opp_cand_r2.155-5AK	19	34	1	3	6	0	5	6	5
opp_cand_r2.156-5AK	24	30	1	2	5	0	5	6	5
opp_cand_r2.248-5AK	23	27	2	2	5	1	5	6	5
opp_cand.1-1AB	29	37	3	5	4	0	5	-	1
opp_cand.2-1AB	24	34	5	6	3	0	5	-	1
opp_cand_r2.72-1AB	23	31	5	7	2	0	5	-	1
opp_cand_r3.177-1AE	13	28	2	7	3	0	5	-	1
opp_cand_r2.3-1AH	25	35	1	5	2	0	5	-	1
opp_cand_r2.270-1G	20	28	2	7	2	0	5	-	1
opp_cand.13-1I	26	28	5	5	1	1	5	-	1
opp_cand_r5.48-1M	20	28	3	6	2	0	5	-	1
opp_cand.192-1N	27	24	3	6	1	0	5	-	1
opp_cand_r2.61-2AI	12	31	4	4	1	0	5	-	2
opp_cand_r2.14-2AT	10	12	0	1	3	0	5	-	2
opp_cand_r5.168-2BB	21	20	1	2	0	0	5	-	2
opp_cand_r5.455-2D	21	21	2	4	3	2	5	-	2
opp_cand_r3.82-4A	25	22	7	8	3	0	5	-	4
opp_cand_r3.85-4A	24	23	6	8	3	0	5	-	4
opp_cand_r3.93-4A	22	21	5	5	2	0	5	-	4
opp_cand.437-4AB	17	12	1	6	3	4	5	-	4
opp_cand.483-4AB	18	15	1	7	2	3	5	-	4
opp_cand.488-4AB	17	18	5	7	2	3	5	-	4
opp_cand.467-4AC	24	16	3	7	1	0	5	-	4
opp_cand.468-4AC	25	13	4	8	2	0	5	-	4
opp_cand.479-4AC	25	15	4	7	2	0	5	-	4
opp_cand_r2.214-4C	26	23	5	15	1	0	5	-	4
opp_cand_r2.216-4C	24	27	6	14	1	0	5	-	4
opp_cand_r3.76-4C	26	26	8	10	0	0	5	-	4
opp_cand_r3.78-4C	26	21	5	5	2	0	5	-	4
opp_cand_r3.81-4C	26	15	12	8	2	0	5	-	4
opp_cand_r3.83-4C	26	22	3	8	1	0	5	-	4
opp_cand_r3.84-4C	26	20	8	9	1	0	5	-	4
opp_cand_r3.86-4C	27	24	5	5	1	0	5	-	4
opp_cand_r3.87-4C	28	17	5	6	2	0	5	-	4
opp_cand_r3.88-4C	22	16	5	10	1	0	5	-	4
opp_cand_r5.366-4C	24	22	5	6	1	0	5	-	4
opp_cand_r5.369-4C	24	22	4	8	3	0	5	-	4
opp_cand_r5.370-4C	25	30	7	13	0	0	5	-	4
opp_cand.122-4D	17	21	8	5	1	0	5	-	4
opp_cand.375-4D	21	17	8	2	3	0	5	-	4
opp_cand_r3.1-4D	16	15	5	2	0	0	5	-	4
opp_cand.571-4E	20	25	8	1	2	0	5	-	4
opp_cand.200-4F	19	15	4	3	4	1	5	-	4

opp_cand_r2.83-4F	18	17	6	2	3	1	5	-	4
opp_cand_r2.86-4F	11	13	7	6	3	0	5	-	4
opp_cand_r5.15-4G	11	7	3	1	2	1	5	-	4
opp_cand.70-5AC	14	29	4	2	2	1	5	-	5
opp_cand.300-5AK	22	27	1	3	3	0	5	-	5
opp_cand_r2.247-5AK	24	29	2	2	3	0	5	-	5
opp_cand.336-5AL	25	32	7	8	2	0	5	-	5
opp_cand.338-5AL	15	35	11	12	2	0	5	-	5
opp_cand.119-5AN	20	41	4	2	1	0	5	-	5
opp_cand.318-5AP	21	38	11	3	2	0	5	-	5
opp_cand.320-5AP	23	39	6	2	2	0	5	-	5
opp_cand.368-5AS	14	30	2	0	1	0	5	-	5
opp_cand_r3.211-5AX	16	22	1	2	0	1	5	-	5
opp_cand_r3.213-5AX	17	28	5	3	0	0	5	-	5
opp_cand_r2.148-2T	18	21	1	6	6	1	6	8	2
opp_cand_r2.154-2G	21	28	5	5	5	1	6	10	2
opp_cand_r2.62-2G	21	28	5	5	5	1	6	10	2
opp_cand_r2.58-2B	18	18	4	5	1	0	6	-	2
opp_cand_r5.456-2D	19	19	1	7	4	1	6	-	2
opp_cand.169-2G	14	23	4	6	1	1	6	-	2
opp_cand.170-2G	13	29	5	6	1	1	6	-	2
opp_cand.173-2G	13	24	4	6	2	1	6	-	2
opp_cand.290-2G	18	32	4	5	2	1	6	-	2
opp_cand.165-2H	9	15	0	5	2	0	6	-	2
opp_cand.167-2H	19	25	2	5	4	1	6	-	2
opp_cand.163-2N	21	19	1	5	4	1	6	-	2
opp_cand.161-2W	19	21	4	6	1	0	6	-	2
opp_cand_r2.43-2W	17	25	7	6	2	0	6	-	2
opp_cand_r3.199-2W	15	30	6	6	2	0	6	-	2
opp_cand.175-2B	14	21	5	7	2	0	7	-	2
opp_cand_r2.48-2B	15	30	1	7	2	0	7	-	2
opp_cand_r2.151-2T	19	24	1	5	3	0	7	-	2
opp_cand.277-2Y	13	21	3	5	4	0	7	-	2
opp_cand_r2.153-2G	19	31	3	3	5	0	8	10	2
opp_cand_r2.56-2H	17	21	0	2	6	1	8	10	2
opp_cand_r2.59-2H	14	27	0	2	6	0	8	10	2
opp_cand.158-2K	20	23	2	0	3	0	8	-	2
opp_cand_r3.282-2L	16	14	2	1	2	1	8	-	2
opp_cand_r5.88-2Q	19	19	2	0	1	0	8	-	2
opp_cand.79-2R	26	19	3	3	2	0	8	-	2
opp_cand_r2.267-2R	16	16	0	2	3	0	8	-	2
opp_cand_r2.146-2T	16	23	0	4	4	0	8	-	2
opp_cand_r3.202-2T	19	23	2	5	8	0	8	-	2
opp_cand_r5.153-2T	15	14	1	3	2	0	8	-	2
opp_cand_r5.154-2T	15	19	0	3	3	0	8	-	2
opp_cand_r5.155-2T	19	26	1	7	3	0	8	-	2
opp_cand.278-2V	17	12	1	3	2	0	8	-	2
opp_cand.279-2V	18	13	1	4	2	0	8	-	2
opp_cand.166-2Y	17	26	3	4	1	0	8	-	2
opp_cand_r3.232-3A	22	21	3	3	3	0	8	-	3
opp_cand_r2.81-4F	19	16	4	3	4	1	8	-	4
opp_cand_r2.15-2AT	9	13	0	1	3	0	9	-	2
opp_cand_r5.152-2AV	20	17	3	0	1	1	9	-	2
opp_cand_r5.169-2BB	14	16	3	1	1	0	9	-	2
opp_cand_r3.206-2D	19	19	1	2	3	2	9	-	2
opp_cand_r3.89-4C	24	21	6	6	0	0	10	5	4

opp_cand_r5.463-2D	18	18	1	3	2	1	10	-	2
opp_cand_r5.464-2D	21	18	2	3	2	2	10	-	2
opp_cand.81-2F	26	19	3	2	2	1	10	-	2
opp_cand.140-4B	20	23	4	3	0	0	10	-	4
opp_cand.142-4B	21	28	4	2	2	0	10	-	4
opp_cand_r3.90-4C	22	21	4	3	0	0	10	-	4
opp_cand_r3.91-4C	20	25	5	13	1	0	10	-	4
opp_cand_r3.92-4C	24	7	5	6	1	1	10	-	4
opp_cand_r3.95-4C	26	13	4	8	2	0	10	-	4
opp_cand_r3.97-4C	17	20	5	12	2	0	10	-	4
opp_cand.466-4P	18	15	4	4	5	0	11	3	4
opp_cand.63-5AC	11	16	1	2	6	1	11	3	5
opp_cand_r2.57-4G	17	13	4	4	5	1	11	4	4
opp_cand_r3.73-4L	26	13	2	3	8	2	11	4	4
opp_cand.106-5B	14	34	7	3	6	0	11	5	5
opp_cand.108-5B	11	33	6	3	6	0	11	5	5
opp_cand.111-5B	16	40	6	0	7	1	11	5	5
opp_cand.113-5B	13	30	3	1	6	2	11	5	5
opp_cand.117-5B	14	41	4	3	7	2	11	5	5
opp_cand_r3.28-5B	17	25	2	1	6	2	11	5	5
opp_cand.292-5AK	25	28	2	3	6	0	11	6	5
opp_cand.18-4G	16	15	5	3	3	0	11	-	4
opp_cand.27-4K	23	19	6	4	1	0	11	-	4
opp_cand.28-4K	15	13	9	3	2	0	11	-	4
opp_cand_r2.176-4M	16	12	4	1	3	0	11	-	4
opp_cand_r3.94-4S	25	19	3	4	0	0	11	-	4
opp_cand.489-4V	19	13	5	5	0	1	11	-	4
opp_cand.494-4V	16	15	5	7	0	0	11	-	4
opp_cand.153-4X	20	18	6	1	0	0	11	-	4
opp_cand_r2.40-4X	24	24	10	3	0	0	11	-	4
opp_cand.24-4Z	24	15	6	2	3	0	11	-	4
opp_cand_r2.262-5A	16	38	3	1	0	0	11	-	5
opp_cand_r3.2-5A	16	36	7	2	1	0	11	-	5
opp_cand_r3.21-5A	20	19	8	1	1	3	11	-	5
opp_cand_r3.3-5A	16	27	6	2	1	0	11	-	5
opp_cand_r3.8-5A	17	24	4	1	0	0	11	-	5
opp_cand.64-5AC	17	16	2	2	4	1	11	-	5
opp_cand.71-5AC	16	16	5	1	4	1	11	-	5
opp_cand.317-5AP	22	39	8	3	1	0	11	-	5
opp_cand.316-5AR	18	25	4	3	5	0	11	-	5
opp_cand_r2.36-5AT	22	34	7	4	0	0	11	-	5
opp_cand_r3.214-5AX	16	23	1	2	0	1	11	-	5
opp_cand.105-5B	17	36	7	0	4	1	11	-	5
opp_cand.476-4AB	20	17	3	4	6	3	12	8	4
opp_cand.174-2G	11	28	4	4	2	2	12	-	2
opp_cand_r3.70-4AA	24	16	5	3	1	2	12	-	4
opp_cand.470-4AB	15	15	5	4	3	4	12	-	4
opp_cand.484-4AB	15	16	5	4	2	4	12	-	4
opp_cand.45-6C	21	14	3	6	7	2	-	1	6
opp_cand.55-6C	26	21	0	9	9	4	-	1	6
opp_cand.56-6C	21	20	2	10	8	2	-	1	6
opp_cand_r2.203-6C	26	21	0	9	9	4	-	1	6
opp_cand_r2.208-6C	25	18	2	8	8	5	-	1	6
opp_cand_r2.210-6C	25	18	2	6	9	4	-	1	6
opp_cand_r2.269-6C	23	20	4	9	9	0	-	1	6
opp_cand_r3.143-6C	23	24	2	13	9	0	-	1	6

opp_cand_r3.144-6C	24	28	2	15	10	0	-	1	6
opp_cand_r3.145-6C	19	27	5	9	7	0	-	1	6
opp_cand_r3.146-6C	24	27	3	9	9	0	-	1	6
opp_cand_r3.147-6C	23	21	1	10	6	1	-	1	6
opp_cand_r3.148-6C	18	19	3	8	16	0	-	1	6
opp_cand_r3.149-6C	23	22	4	11	7	0	-	1	6
opp_cand_r3.150-6C	15	21	3	4	16	0	-	1	6
opp_cand_r3.151-6C	26	19	1	5	7	0	-	1	6
opp_cand_r3.154-6C	15	19	2	7	14	0	-	1	6
opp_cand_r3.155-6C	17	17	3	8	14	0	-	1	6
opp_cand_r3.156-6C	16	9	3	3	11	1	-	1	6
opp_cand_r3.157-6C	19	19	3	11	5	0	-	1	6
opp_cand_r3.158-6C	17	20	3	7	15	0	-	1	6
opp_cand_r3.159-6C	19	20	1	4	10	0	-	1	6
opp_cand_r3.161-6C	17	16	1	7	12	0	-	1	6
opp_cand_r3.174-6C	7	10	0	3	9	0	-	1	6
opp_cand_r5.201-6C	16	16	2	4	14	1	-	1	6
opp_cand_r5.202-6C	16	13	2	5	11	1	-	1	6
opp_cand_r5.209-6C	17	21	1	2	11	0	-	1	6
opp_cand_r5.221-6C	26	15	2	9	8	0	-	1	6
opp_cand_r5.224-6C	24	21	3	12	10	0	-	1	6
opp_cand_r5.225-6C	23	19	1	9	7	1	-	1	6
opp_cand_r5.206-6C	17	16	0	2	8	0	-	1	6
opp_cand_r5.207-6C	19	18	0	4	12	0	-	1	6
opp_cand_r5.208-6C	18	19	2	4	12	1	-	1	6
opp_cand_r5.210-6C	17	15	1	4	7	0	-	1	6
opp_cand_r5.214-6C	17	17	2	2	6	0	-	1	6
opp_cand_r5.226-6C	23	21	1	10	6	1	-	1	6
opp_cand.588-11G	17	22	4	0	5	1	-	1	11
opp_cand_r5.377-13C	26	35	1	4	5	0	-	1	13
opp_cand.231-52B	11	8	6	5	6	0	-	1	52
opp_cand_r2.108-52B	12	7	5	3	5	0	-	1	52
opp_cand_r3.39-10D	20	29	1	0	6	0	-	2	10
opp_cand_r3.42-10D	18	17	0	1	5	0	-	2	10
opp_cand_r3.43-10D	23	19	1	3	5	0	-	2	10
opp_cand_r3.48-10D	21	19	0	3	6	1	-	2	10
opp_cand_r3.49-10D	24	16	2	1	6	0	-	2	10
opp_cand_r3.55-10N	19	20	2	1	7	1	-	2	10
opp_cand_r3.56-10N	19	21	2	1	7	1	-	2	10
opp_cand.425-8A	18	34	9	2	12	0	-	3	8
opp_cand.427-8A	18	33	9	3	10	0	-	3	8
opp_cand_r3.241-8A	18	35	7	4	7	0	-	3	8
opp_cand.406-8B	13	28	8	3	17	0	-	3	8
opp_cand.417-8B	14	31	5	1	12	1	-	3	8
opp_cand.418-8B	13	30	5	2	15	0	-	3	8
opp_cand.419-8B	14	29	6	2	17	0	-	3	8
opp_cand.421-8B	13	30	7	2	14	1	-	3	8
opp_cand.423-8B	18	29	7	4	14	0	-	3	8
opp_cand.424-8B	18	31	10	4	12	0	-	3	8
opp_cand.426-8B	18	31	11	4	12	0	-	3	8
opp_cand_r5.326-8B	10	21	5	0	10	0	-	3	8
opp_cand.376-8D	15	28	4	5	7	0	-	3	8
opp_cand.412-8D	20	35	7	5	8	1	-	3	8
opp_cand_r3.239-8D	18	25	6	4	8	1	-	3	8
opp_cand_r3.240-8D	23	39	8	3	6	0	-	3	8
opp_cand_r3.242-8D	22	40	7	4	7	0	-	3	8

opp_cand_r3.52-8D	17	34	2	3	5	1	-	3	8
opp_cand_r3.58-8D	15	37	2	2	5	1	-	3	8
opp_cand_r3.60-8D	17	37	5	3	5	1	-	3	8
opp_cand_r5.162-8D	14	34	5	3	6	1	-	3	8
opp_cand.396-8F	16	31	6	6	13	3	-	3	8
opp_cand.399-8F	18	42	7	6	11	0	-	3	8
opp_cand.403-8F	21	24	6	5	15	1	-	3	8
opp_cand.405-8F	19	32	6	5	6	2	-	3	8
opp_cand.407-8F	21	28	5	7	9	0	-	3	8
opp_cand.409-8F	20	27	7	5	11	1	-	3	8
opp_cand_r2.187-8F	16	28	1	2	12	1	-	3	8
opp_cand.397-8G	15	31	4	3	14	1	-	3	8
opp_cand.402-8G	15	21	6	3	13	1	-	3	8
opp_cand.408-8G	22	27	2	4	11	1	-	3	8
opp_cand_r2.190-8G	19	22	10	3	15	1	-	3	8
opp_cand_r3.243-8G	16	23	3	3	14	0	-	3	8
opp_cand_r3.244-8G	13	24	6	2	15	0	-	3	8
opp_cand.347-8K	21	29	6	6	5	0	-	3	8
opp_cand_r3.138-8U	19	25	9	4	6	0	-	3	8
opp_cand.345-8V	17	29	1	9	5	3	-	3	8
opp_cand_r2.46-8V	19	29	2	3	5	1	-	3	8
opp_cand.133-13A	20	21	0	1	6	0	-	3	13
opp_cand.210-10J	18	20	2	1	6	0	-	4	10
opp_cand.212-10J	20	21	1	1	5	0	-	4	10
opp_cand.618-10R	10	27	4	1	6	2	-	4	10
opp_cand.618-10R	10	27	4	1	6	2	-	4	10
opp_cand_r5.329-10R	11	23	6	2	6	2	-	4	10
opp_cand_r5.260-56A	21	2	4	10	6	0	-	4	56
opp_cand_r5.397-56A	17	2	2	7	6	0	-	4	56
opp_cand_r5.402-56A	14	2	4	8	6	0	-	4	56
opp_cand_r5.404-56A	16	1	1	7	8	0	-	4	56
opp_cand_r5.405-56A	14	3	3	6	6	0	-	4	56
opp_cand_r3.4-5B	16	25	5	2	9	2	-	5	5
opp_cand_r3.6-5B	18	30	1	0	7	4	-	5	5
opp_cand_r3.9-5B	17	34	3	1	8	4	-	5	5
opp_cand_r5.103-5B	16	31	4	2	6	3	-	5	5
opp_cand_r5.104-5B	15	30	4	1	6	4	-	5	5
opp_cand_r5.110-5B	14	32	3	2	8	1	-	5	5
opp_cand_r5.98-5B	16	34	2	1	8	2	-	5	5
opp_cand_r5.51-13H	23	23	2	1	5	1	-	5	13
opp_cand.61-1E	21	29	4	2	6	0	-	6	1
opp_cand_r3.140-8U	12	24	6	8	5	0	-	6	8
opp_cand.454-8W	22	27	6	1	6	1	-	6	8
opp_cand.455-8W	20	24	5	2	6	0	-	6	8
opp_cand.246-51A	15	5	10	5	6	0	-	7	51
opp_cand.247-51A	16	5	7	4	5	0	-	7	51
opp_cand_r3.353-51A	14	9	7	3	6	0	-	7	51
opp_cand_r3.355-51A	13	7	8	2	7	0	-	7	51
opp_cand_r3.359-51A	11	10	7	2	5	1	-	7	51
opp_cand_r3.360-51A	8	7	2	3	5	0	-	7	51
opp_cand_r3.378-51A	13	8	8	4	6	0	-	7	51
opp_cand_r3.379-51A	12	10	7	3	5	0	-	7	51
opp_cand_r2.134-51AC	10	6	6	4	5	0	-	7	51
opp_cand.261-51AD	16	5	8	9	6	0	-	7	51
opp_cand.263-51AD	19	8	8	6	6	0	-	7	51
opp_cand.264-51AD	19	7	8	4	5	0	-	7	51

opp_cand_r5.257-51AI	10	4	5	3	8	0	-	7	51
opp_cand_r2.123-51C	13	13	5	5	7	0	-	7	51
opp_cand_r2.137-51C	11	13	5	5	9	1	-	7	51
opp_cand_r3.249-51C	12	5	2	1	8	0	-	7	51
opp_cand_r3.280-51C	15	13	6	4	8	0	-	7	51
opp_cand_r2.126-51F	13	14	3	0	7	0	-	7	51
opp_cand_r3.281-51F	12	9	6	4	9	0	-	7	51
opp_cand_r5.61-51F	13	8	7	2	5	0	-	7	51
opp_cand_r5.62-51F	13	11	6	4	7	0	-	7	51
opp_cand_r3.351-51G	12	7	10	4	8	0	-	7	51
opp_cand_r3.309-51H	10	7	15	7	5	0	-	7	51
opp_cand_r3.331-51I	15	6	9	4	5	0	-	7	51
opp_cand.262-51L	15	10	8	7	8	0	-	7	51
opp_cand_r3.352-51P	10	6	8	8	7	0	-	7	51
opp_cand.255-51Q	9	6	5	5	7	0	-	7	51
opp_cand_r3.318-52AG	12	11	7	8	3	0	-	7	52
opp_cand_r3.328-52AG	10	8	4	8	5	0	-	7	52
opp_cand.351-8K	20	21	5	7	6	1	-	8	8
opp_cand_r5.375-13C	24	34	4	4	5	0	-	8	13
opp_cand.346-8J	16	27	6	5	5	0	-	9	8
opp_cand_r5.250-52B	14	10	5	2	6	0	-	9	52
opp_cand_r2.129-52E	13	10	17	3	6	0	-	9	52
opp_cand_r2.130-52E	15	8	16	2	6	1	-	9	52
opp_cand_r3.301-52E	23	11	14	3	6	1	-	9	52
opp_cand_r3.320-52E	19	10	15	6	10	0	-	9	52
opp_cand_r3.321-52E	18	9	14	6	7	0	-	9	52
opp_cand_r5.273-52E	23	13	16	5	7	0	-	9	52
opp_cand_r5.274-52E	21	10	14	6	8	0	-	9	52
opp_cand_r5.408-52E	20	9	14	4	5	0	-	9	52
opp_cand_r5.411-52E	20	8	15	5	5	0	-	9	52
opp_cand_r5.413-52E	21	9	17	4	5	1	-	9	52
opp_cand_r5.415-52E	20	10	14	6	6	0	-	9	52
opp_cand_r3.303-52J	14	7	14	8	5	0	-	9	52
opp_cand.232-52B	12	9	6	3	5	0	-	11	52
opp_cand.242-52H	12	10	10	4	8	0	-	11	52
opp_cand.243-52H	14	10	8	7	7	0	-	11	52
opp_cand.222-52N	14	4	10	8	7	0	-	11	52
opp_cand.224-52N	14	5	9	3	7	0	-	11	52
opp_cand_r2.105-52N	9	6	5	6	6	0	-	11	52
opp_cand_r2.106-52N	9	6	10	6	7	0	-	11	52
opp_cand_r2.107-52N	11	8	9	4	6	0	-	11	52
opp_cand_r2.71-1J	22	27	5	3	5	0	-	12	1
opp_cand_r5.175-1J	27	33	3	2	6	0	-	12	1
opp_cand_r5.341-10AG	22	20	3	8	5	2	-	12	10
opp_cand_r5.303-10AG	25	19	4	4	5	3	-	12	10
opp_cand_r5.348-10G	18	26	6	6	5	4	-	12	10
opp_cand_r5.364-10G	23	27	2	8	5	5	-	12	10
opp_cand_r3.44-10G	14	16	1	2	6	0	-	12	10
opp_cand_r3.45-10G	15	16	1	3	5	1	-	12	10
opp_cand_r3.46-10G	12	20	2	3	7	1	-	12	10
opp_cand_r3.47-10G	9	18	2	2	5	1	-	12	10
opp_cand.435-13T	25	16	0	2	5	3	-	12	13
opp_cand_r2.201-13T	18	27	3	4	5	4	-	12	13
opp_cand_r3.175-1AB	30	29	3	4	2	0	-	-	1
opp_cand_r3.176-1AB	20	29	5	4	2	0	-	-	1
opp_cand_r5.10-1AB	31	29	3	4	4	0	-	-	1

opp_cand_r5.9-1AB	22	27	3	3	3	0	-	-	1
opp_cand.187-1AH	25	33	3	3	2	0	-	-	1
opp_cand_r5.127-1AJ	20	27	5	1	2	0	-	-	1
opp_cand_r3.197-1AK	16	14	2	1	2	0	-	-	1
opp_cand_r5.132-1AN	15	19	1	1	1	0	-	-	1
opp_cand.196-1B	9	15	0	1	2	0	-	-	1
opp_cand_r2.75-1B	9	5	2	0	2	0	-	-	1
opp_cand_r2.74-1D	24	22	5	4	0	0	-	-	1
opp_cand_r2.233-1E	18	36	5	2	3	1	-	-	1
opp_cand.198-1F	24	34	3	4	4	1	-	-	1
opp_cand.283-1F	18	30	1	1	3	0	-	-	1
opp_cand.185-1K	20	24	1	3	1	0	-	-	1
opp_cand.188-1L	21	22	5	4	1	0	-	-	1
opp_cand_r2.73-1M	20	28	2	4	4	0	-	-	1
opp_cand.124-1N	19	22	0	2	0	0	-	-	1
opp_cand.193-1N	20	30	5	3	1	0	-	-	1
opp_cand_r3.192-1N	17	24	3	0	0	0	-	-	1
opp_cand_r5.129-1N	20	33	4	1	1	0	-	-	1
opp_cand_r3.233-1P	15	15	0	3	0	0	-	-	1
opp_cand.191-1Q	18	21	0	2	2	0	-	-	1
opp_cand_r2.1-1S	13	25	1	3	3	0	-	-	1
opp_cand_r2.2-1S	10	20	1	3	3	0	-	-	1
opp_cand.85-2A	28	23	4	2	1	0	-	-	2
opp_cand.86-2A	24	22	3	2	1	0	-	-	2
opp_cand.87-2A	27	21	2	1	1	0	-	-	2
opp_cand.91-2A	29	23	1	1	2	0	-	-	2
opp_cand.92-2A	28	23	3	1	2	0	-	-	2
opp_cand.93-2A	26	22	2	0	2	0	-	-	2
opp_cand_r2.273-2A	22	19	2	2	2	0	-	-	2
opp_cand.39-2AF	16	36	1	2	1	0	-	-	2
opp_cand_r3.235-2AG	12	12	4	2	1	0	-	-	2
opp_cand_r2.49-2AI	8	16	1	1	3	0	-	-	2
opp_cand_r5.99-5B	16	23	5	4	4	2	-	-	5
opp_cand.95-5BA	22	18	3	0	2	1	-	-	5
opp_cand_r3.12-5BB	15	18	9	2	1	4	-	-	5
opp_cand_r3.17-5BB	16	21	7	1	2	3	-	-	5
opp_cand_r3.19-5BB	16	20	8	2	2	3	-	-	5
opp_cand_r3.22-5BB	16	20	8	2	2	4	-	-	5
opp_cand_r3.5-5BB	18	17	8	3	2	4	-	-	5
opp_cand_r3.7-5BB	16	19	8	2	2	4	-	-	5
opp_cand.358-5BE	12	24	1	0	0	0	-	-	5
opp_cand_r5.167-5BF	10	14	1	1	3	1	-	-	5
opp_cand_r5.387-5BF	16	18	2	1	1	0	-	-	5
opp_cand_r5.73-5BF	20	20	1	1	1	1	-	-	5
opp_cand_r5.76-5BF	16	17	2	1	1	2	-	-	5
opp_cand.186-5C	16	25	6	5	3	0	-	-	5
opp_cand.460-5D	20	39	7	2	0	3	-	-	5
opp_cand.462-5D	23	30	6	1	3	3	-	-	5
opp_cand.464-5D	17	22	6	0	1	2	-	-	5
opp_cand_r3.110-5D	16	15	5	1	1	2	-	-	5
opp_cand_r3.112-5D	21	18	7	1	2	2	-	-	5
opp_cand_r3.114-5D	24	27	6	2	2	0	-	-	5
opp_cand_r3.115-5D	17	25	7	2	3	0	-	-	5
opp_cand_r3.116-5D	16	26	4	1	1	1	-	-	5
opp_cand_r3.117-5D	14	28	8	2	1	1	-	-	5
opp_cand_r5.365-5D	19	22	5	0	2	2	-	-	5

opp_cand.428-5E	15	34	5	3	0	0	-	-	5
opp_cand.370-5F	20	37	5	0	0	0	-	-	5
opp_cand_r2.168-5F	26	35	4	0	1	0	-	-	5
opp_cand.294-5G	27	35	6	1	2	0	-	-	5
opp_cand.297-5G	26	31	6	2	1	4	-	-	5
opp_cand.302-5G	25	35	7	1	3	3	-	-	5
opp_cand_r2.158-5G	24	24	3	0	0	4	-	-	5
opp_cand_r2.159-5G	24	25	3	1	2	2	-	-	5
opp_cand.452-5I	22	34	4	2	1	0	-	-	5
opp_cand.359-5J	22	35	8	2	2	0	-	-	5
opp_cand.360-5J	23	25	5	0	2	0	-	-	5
opp_cand.361-5J	25	30	6	0	2	1	-	-	5
opp_cand.362-5J	21	28	5	0	1	1	-	-	5
opp_cand.363-5J	22	27	3	0	2	0	-	-	5
opp_cand.72-5K	17	21	3	0	4	0	-	-	5
opp_cand.73-5K	17	17	5	2	1	0	-	-	5
opp_cand.74-5K	21	20	3	0	4	0	-	-	5
opp_cand.459-5L	16	32	6	4	1	0	-	-	5
opp_cand_r2.204-5L	19	34	8	4	1	0	-	-	5
opp_cand.322-5M	22	16	1	6	0	0	-	-	5
opp_cand.325-5M	28	34	7	0	0	0	-	-	5
opp_cand.328-5M	23	33	6	6	1	0	-	-	5
opp_cand.329-5M	19	20	7	7	4	0	-	-	5
opp_cand.330-5M	19	21	5	6	1	0	-	-	5
opp_cand.331-5M	18	21	6	6	3	0	-	-	5
opp_cand.332-5M	20	26	8	4	0	1	-	-	5
opp_cand.341-5M	13	27	5	6	4	0	-	-	5
opp_cand_r2.163-5M	20	27	6	2	3	1	-	-	5
opp_cand_r2.165-5M	23	26	2	3	2	0	-	-	5
opp_cand_r2.169-5M	14	25	8	3	1	0	-	-	5
opp_cand_r5.300-5M	17	25	5	7	2	0	-	-	5
opp_cand.36-5CA	17	33	7	1	2	0	-	-	5
opp_cand.379-5P	19	29	3	3	1	1	-	-	5
opp_cand.384-5P	20	28	3	6	3	0	-	-	5
opp_cand_r3.221-5P	21	27	3	1	0	0	-	-	5
opp_cand_r3.222-5P	22	36	3	3	1	2	-	-	5
opp_cand_r3.230-5P	16	32	3	1	3	0	-	-	5
opp_cand_r3.231-5P	14	29	2	2	3	0	-	-	5
opp_cand_r5.312-5P	26	34	3	4	1	1	-	-	5
opp_cand_r5.313-5P	20	29	3	2	0	1	-	-	5
opp_cand_r2.173-5T	16	35	10	5	1	0	-	-	5
opp_cand_r5.191-5X	15	13	2	2	2	3	-	-	5
opp_cand_r5.182-5X	15	18	0	1	4	2	-	-	5
opp_cand_r5.199-5X	10	16	0	2	1	4	-	-	5
opp_cand_r5.190-5X	12	15	1	3	2	3	-	-	5
opp_cand_r5.195-5X	13	15	1	3	3	2	-	-	5
opp_cand_r3.20-5X	12	22	1	2	0	2	-	-	5
opp_cand_r3.237-5X	8	18	0	0	2	1	-	-	5
opp_cand_r3.25-5X	16	14	0	1	4	1	-	-	5
opp_cand_r3.274-5X	14	15	1	2	2	2	-	-	5
opp_cand_r2.258-6A	24	26	1	0	1	0	-	-	6
opp_cand.136-6AE	23	25	1	1	2	0	-	-	6
opp_cand_r3.226-6AF	16	21	5	1	6	0	-	-	6
opp_cand.129-6B	16	34	1	1	2	0	-	-	6
opp_cand.84-6B	18	26	2	1	1	0	-	-	6
opp_cand_r2.17-6B	14	35	2	0	1	0	-	-	6

opp_cand_r2.18-6B	19	24	2	0	1	0	-	-	6
opp_cand_r3.247-6B	12	28	2	1	2	0	-	-	6
opp_cand_r5.423-6B	13	24	2	0	1	0	-	-	6
opp_cand.44-6C	22	19	2	6	2	1	-	-	6
opp_cand_r3.152-6C	29	22	3	12	2	0	-	-	6
opp_cand_r5.173-6C	21	17	1	11	2	0	-	-	6
opp_cand.131-6D	16	9	0	3	2	0	-	-	6
opp_cand_r2.20-6D	10	10	1	2	1	1	-	-	6
opp_cand_r2.22-6E	23	32	1	2	2	0	-	-	6
opp_cand_r3.66-6E	19	28	1	2	1	0	-	-	6
opp_cand_r2.272-6F	21	25	4	0	0	0	-	-	6
opp_cand.570-6J	24	24	4	0	1	0	-	-	6
opp_cand.285-6K	19	14	5	1	3	0	-	-	6
opp_cand.286-6K	17	19	1	1	3	0	-	-	6
opp_cand.288-6K	19	16	1	0	3	0	-	-	6
opp_cand.289-6K	25	15	0	0	2	0	-	-	6
opp_cand_r2.152-6K	20	19	3	0	1	0	-	-	6
opp_cand.377-6M	20	28	0	1	4	1	-	-	6
opp_cand_r5.7-6N	25	23	0	0	3	0	-	-	6
opp_cand_r2.271-6P	14	26	2	0	1	0	-	-	6
opp_cand.96-6Q	19	34	1	2	0	0	-	-	6
opp_cand_r3.201-6R	17	27	1	1	0	0	-	-	6
opp_cand_r3.67-6S	13	17	6	0	1	0	-	-	6
opp_cand_r3.53-6T	17	17	4	1	1	0	-	-	6
opp_cand_r3.57-6T	14	19	2	0	0	0	-	-	6
opp_cand_r3.61-6T	14	13	1	0	0	0	-	-	6
opp_cand_r3.62-6T	9	16	1	0	0	0	-	-	6
opp_cand.57-6U	16	21	2	2	3	0	-	-	6
opp_cand.77-6V	17	19	2	0	0	0	-	-	6
opp_cand.78-6W	16	22	0	0	0	0	-	-	6
opp_cand.378-6X	17	34	3	2	2	0	-	-	6
opp_cand_r5.292-6Y	17	30	1	1	1	0	-	-	6
opp_cand_r5.77-6AH	14	14	3	1	1	0	-	-	6
opp_cand_r5.78-6AH	12	16	2	0	1	0	-	-	6
opp_cand.495-7C	24	22	2	2	1	0	-	-	7
opp_cand.544-7C	15	23	3	6	2	0	-	-	7
opp_cand.553-7C	16	35	4	2	4	1	-	-	7
opp_cand_r2.261-7C	19	30	5	2	1	1	-	-	7
opp_cand.12-7D	19	33	5	3	1	1	-	-	7
opp_cand.512-7D	19	25	4	3	1	0	-	-	7
opp_cand.504-7P	25	28	5	3	1	1	-	-	7
opp_cand_r3.59-8A	21	42	2	1	7	0	-	-	8
opp_cand.137-8AA	14	16	0	1	0	0	-	-	8
opp_cand.374-8D	15	38	2	2	4	0	-	-	8
opp_cand.353-8J	17	28	9	3	2	0	-	-	8
opp_cand_r3.135-8K	18	29	6	11	7	1	-	-	8
opp_cand_r3.136-8K	17	20	4	5	1	1	-	-	8
opp_cand.333-8U	22	35	7	7	4	0	-	-	8
opp_cand.335-8U	21	32	7	6	3	0	-	-	8
opp_cand.339-8U	25	30	7	10	3	0	-	-	8
opp_cand_r2.222-8U	23	26	9	7	4	1	-	-	8
opp_cand_r3.131-8U	21	27	7	6	3	2	-	-	8
opp_cand_r3.133-8U	22	24	8	9	4	0	-	-	8
opp_cand_r3.134-8U	19	24	11	8	4	0	-	-	8
opp_cand_r3.137-8U	22	25	7	6	5	2	-	-	8
opp_cand_r3.139-8U	22	27	7	2	4	3	-	-	8

opp_cand_r5.298-8U	22	24	6	5	2	2	-	-	8
opp_cand_r5.299-8U	20	26	7	7	4	2	-	-	8
opp_cand.342-8V	19	27	6	2	4	0	-	-	8
opp_cand.343-8V	20	24	4	6	2	1	-	-	8
opp_cand_r3.128-8V	16	23	3	6	3	1	-	-	8
opp_cand_r3.129-8V	20	25	2	5	2	1	-	-	8
opp_cand_r3.130-8V	19	27	6	6	6	3	-	-	8
opp_cand_r3.132-8V	20	25	5	6	3	1	-	-	8
opp_cand.453-8W	18	25	5	1	3	0	-	-	8
opp_cand.75-9A	12	15	1	0	0	1	-	-	9
opp_cand_r2.250-9A	16	12	2	0	2	0	-	-	9
opp_cand.303-9G	9	27	1	0	4	0	-	-	9
opp_cand.307-9G	17	26	4	1	4	0	-	-	9
opp_cand.308-9G	13	31	5	0	5	0	-	-	9
opp_cand.310-9G	15	30	4	1	5	0	-	-	9
opp_cand.311-9G	14	22	8	0	1	0	-	-	9
opp_cand.314-9G	14	19	9	0	2	0	-	-	9
opp_cand.101-9I	18	32	6	1	2	0	-	-	9
opp_cand.97-9I	20	21	6	2	3	0	-	-	9
opp_cand.98-9I	18	21	4	0	1	0	-	-	9
opp_cand.216-9K	13	28	9	1	2	1	-	-	9
opp_cand.217-9K	14	24	1	1	2	1	-	-	9
opp_cand.218-9K	17	21	1	1	1	1	-	-	9
opp_cand_r3.100-9M	21	26	6	1	0	0	-	-	9
opp_cand_r2.94-9R	15	26	3	2	3	0	-	-	9
opp_cand_r3.227-9Z	13	15	1	2	2	0	-	-	9
opp_cand.215-10A	23	22	3	0	1	0	-	-	10
opp_cand.40-10A	15	28	2	3	3	1	-	-	10
opp_cand.41-10A	21	29	4	1	0	0	-	-	10
opp_cand_r2.35-10A	16	22	3	1	0	0	-	-	10
opp_cand_r2.92-10A	18	29	4	2	2	0	-	-	10
opp_cand_r3.142-10AA	13	15	2	0	2	1	-	-	10
opp_cand.80-10AC	10	9	6	0	0	0	-	-	10
opp_cand.430-10AG	25	28	4	7	4	1	-	-	10
opp_cand.431-10AG	27	22	5	8	3	0	-	-	10
opp_cand.436-10AG	23	18	2	0	4	1	-	-	10
opp_cand.458-10AG	28	19	3	5	3	2	-	-	10
opp_cand_r2.91-10AG	23	23	6	3	4	1	-	-	10
opp_cand_r3.104-10AG	25	28	1	1	3	3	-	-	10
opp_cand_r3.106-10AG	19	25	3	7	2	5	-	-	10
opp_cand_r3.107-10AG	18	18	4	5	0	7	-	-	10
opp_cand_r5.174-10AH	16	11	2	0	1	0	-	-	10
opp_cand_r2.78-10AK	19	23	2	0	0	1	-	-	10
opp_cand_r3.218-10AK	20	22	2	0	1	1	-	-	10
opp_cand_r3.219-10AK	20	23	2	0	0	1	-	-	10
opp_cand.168-10AL	20	16	3	1	1	0	-	-	10
opp_cand_r2.16-10AM	22	19	1	2	0	1	-	-	10
opp_cand.179-10C	19	17	1	0	0	0	-	-	10
opp_cand_r3.51-10D	19	18	0	1	4	0	-	-	10
opp_cand_r5.338-10G	23	20	1	3	5	2	-	-	10
opp_cand_r5.358-10G	21	22	5	5	2	4	-	-	10
opp_cand_r5.336-10G	23	16	1	1	3	3	-	-	10
opp_cand_r5.337-10G	24	27	2	1	4	3	-	-	10
opp_cand_r5.357-10G	21	25	4	4	3	3	-	-	10
opp_cand.573-10G	20	29	2	2	3	0	-	-	10
opp_cand.574-10G	16	15	0	2	3	0	-	-	10

opp_cand.575-10G	18	16	1	2	3	0	-	-	10
opp_cand_r2.12-10G	14	28	2	1	2	0	-	-	10
opp_cand_r2.219-10G	17	17	1	3	4	1	-	-	10
opp_cand_r3.41-10G	16	19	1	4	3	1	-	-	10
opp_cand.4-10H	21	20	1	4	2	0	-	-	10
opp_cand.5-10H	19	16	0	6	2	0	-	-	10
opp_cand_r2.160-10H	20	16	1	1	2	1	-	-	10
opp_cand.211-10J	18	21	3	0	2	1	-	-	10
opp_cand.213-10J	18	22	3	0	3	0	-	-	10
opp_cand_r2.85-10J	20	14	3	1	4	0	-	-	10
opp_cand.611-10K	17	20	3	1	1	0	-	-	10
opp_cand.613-10K	19	25	1	0	2	1	-	-	10
opp_cand_r2.224-10K	21	24	1	1	2	0	-	-	10
opp_cand_r3.50-10N	18	14	3	2	4	1	-	-	10
opp_cand_r3.54-10N	19	19	2	2	4	1	-	-	10
opp_cand.51-10P	14	23	1	4	1	2	-	-	10
opp_cand.52-10P	20	27	1	4	2	1	-	-	10
opp_cand.53-10P	18	21	1	4	3	1	-	-	10
opp_cand.54-10P	19	19	1	5	3	1	-	-	10
opp_cand_r2.205-10P	18	26	1	3	2	1	-	-	10
opp_cand_r2.96-10P	18	27	2	4	1	2	-	-	10
opp_cand.102-10Q	20	27	4	2	2	0	-	-	10
opp_cand.103-10Q	19	24	1	1	2	0	-	-	10
opp_cand_r2.150-10Q	21	30	2	1	1	0	-	-	10
opp_cand_r2.4-10Q	19	23	2	1	1	0	-	-	10
opp_cand_r3.23-10Q	18	19	1	1	2	0	-	-	10
opp_cand_r5.97-10Q	18	16	2	3	2	1	-	-	10
opp_cand.615-10R	10	25	3	2	0	1	-	-	10
opp_cand.616-10R	11	27	3	0	4	2	-	-	10
opp_cand.617-10R	9	22	7	1	3	1	-	-	10
opp_cand_r2.234-10R	12	26	3	1	2	0	-	-	10
opp_cand_r5.276-10R	9	21	2	2	1	1	-	-	10
opp_cand_r5.291-10R	10	18	3	2	2	2	-	-	10
opp_cand_r3.40-10S	15	17	3	3	4	0	-	-	10
opp_cand.612-10T	15	24	2	0	4	0	-	-	10
opp_cand_r3.153-10U	20	18	2	4	2	0	-	-	10
opp_cand_r5.220-10U	18	17	3	4	2	0	-	-	10
opp_cand.120-10V	20	15	1	2	3	0	-	-	10
opp_cand_r5.158-10X	9	27	1	1	2	0	-	-	10
opp_cand_r5.293-10X	8	25	1	1	2	0	-	-	10
opp_cand_r3.13-10Y	20	13	3	2	1	0	-	-	10
opp_cand_r5.279-10Z	10	21	2	1	4	0	-	-	10
opp_cand.178-11A	19	21	1	1	0	0	-	-	11
opp_cand.580-11G	19	28	3	2	4	1	-	-	11
opp_cand.581-11G	20	27	4	1	4	2	-	-	11
opp_cand.582-11G	20	19	2	1	2	0	-	-	11
opp_cand.584-11G	15	28	4	2	3	0	-	-	11
opp_cand.586-11G	16	30	3	3	4	0	-	-	11
opp_cand.591-11G	15	22	5	1	4	1	-	-	11
opp_cand.594-11G	14	26	4	2	4	0	-	-	11
opp_cand.576-11H	19	26	2	1	3	0	-	-	11
opp_cand.577-11H	20	23	1	0	1	0	-	-	11
opp_cand.578-11H	22	21	4	0	8	0	-	-	11
opp_cand_r2.228-11H	18	18	2	0	1	1	-	-	11
opp_cand.592-11J	14	20	2	0	4	0	-	-	11
opp_cand.596-11J	15	24	3	1	5	0	-	-	11

opp_cand.600-11J	17	25	0	0	4	0	-	-	11
opp_cand.603-11K	16	16	4	3	2	0	-	-	11
opp_cand_r3.200-11L	9	25	2	1	2	1	-	-	11
opp_cand_r3.195-11W	14	20	0	2	2	0	-	-	11
opp_cand.177-12D	16	16	3	0	1	1	-	-	12
opp_cand_r5.172-12D	19	17	3	0	1	0	-	-	12
opp_cand.429-12E	22	28	4	2	4	3	-	-	12
opp_cand.439-12E	24	24	5	9	3	3	-	-	12
opp_cand.457-12E	26	23	5	6	2	2	-	-	12
opp_cand_r2.93-12E	22	25	4	9	4	4	-	-	12
opp_cand_r3.103-12E	23	25	1	6	3	2	-	-	12
opp_cand_r3.105-12E	24	19	3	4	2	2	-	-	12
opp_cand_r3.108-12E	20	19	4	10	3	2	-	-	12
opp_cand_r3.111-12E	21	24	1	3	2	2	-	-	12
opp_cand_r3.113-12E	19	20	4	10	3	4	-	-	12
opp_cand.189-12K	24	22	3	1	0	0	-	-	12
opp_cand.190-12K	23	23	3	1	0	0	-	-	12
opp_cand.197-12K	18	27	3	1	1	0	-	-	12
opp_cand.132-13A	19	15	0	1	4	1	-	-	13
opp_cand_r2.24-13A	19	20	0	1	0	2	-	-	13
opp_cand_r3.184-13A	20	16	1	4	3	3	-	-	13
opp_cand_r3.185-13A	18	16	1	4	3	3	-	-	13
opp_cand_r5.140-13A	19	21	0	1	1	2	-	-	13
opp_cand_r5.379-13AE	11	19	1	0	2	0	-	-	13
opp_cand_r2.76-13AF	17	25	2	1	3	0	-	-	13
opp_cand_r3.225-13AF	18	24	1	1	2	1	-	-	13
opp_cand_r3.190-13AG	23	26	1	0	1	1	-	-	13
opp_cand_r3.191-13AG	19	18	0	0	1	2	-	-	13
opp_cand.387-13C	22	36	4	1	2	1	-	-	13
opp_cand_r2.183-13C	19	35	2	1	2	1	-	-	13
opp_cand_r3.170-13C	26	34	1	4	4	0	-	-	13
opp_cand_r5.376-13C	24	35	0	2	4	2	-	-	13
opp_cand_r5.378-13C	27	37	0	4	6	0	-	-	13
opp_cand.386-13D	23	29	0	3	3	0	-	-	13
opp_cand.389-13D	21	29	1	1	0	0	-	-	13
opp_cand.393-13D	22	30	2	2	2	0	-	-	13
opp_cand.394-13D	26	32	1	4	1	1	-	-	13
opp_cand.395-13D	23	34	1	4	1	1	-	-	13
opp_cand_r2.180-13D	21	31	3	3	1	0	-	-	13
opp_cand_r2.23-13D	20	30	1	4	2	1	-	-	13
opp_cand_r3.171-13D	25	39	2	3	3	0	-	-	13
opp_cand_r3.179-13D	22	33	1	4	1	0	-	-	13
opp_cand_r5.240-13D	22	29	3	4	2	0	-	-	13
opp_cand_r5.317-13D	24	26	0	2	3	1	-	-	13
opp_cand_r5.319-13D	22	26	2	2	3	1	-	-	13
opp_cand_r5.428-13D	23	29	3	4	2	0	-	-	13
opp_cand_r2.184-13E	27	29	4	2	2	2	-	-	13
opp_cand_r2.186-13E	25	20	4	2	3	2	-	-	13
opp_cand_r3.172-13E	25	23	2	2	3	2	-	-	13
opp_cand_r5.374-13E	28	28	3	2	2	2	-	-	13
opp_cand_r5.156-13G	13	19	2	1	2	1	-	-	13
opp_cand.37-13H	17	19	2	2	2	0	-	-	13
opp_cand_r5.52-13H	17	28	0	0	1	0	-	-	13
opp_cand_r5.373-13J	19	34	2	1	3	1	-	-	13
opp_cand.38-13K	18	32	0	2	2	0	-	-	13
opp_cand_r2.178-13K	16	36	1	1	1	0	-	-	13

opp_cand_r5.328-13L	19	35	1	3	2	2	-	-	13
opp_cand_r5.333-13AI	20	21	4	1	1	0	-	-	13
opp_cand.434-13T	23	25	2	5	1	3	-	-	13
opp_cand.440-13T	21	18	5	4	2	10	-	-	13
opp_cand.441-13T	23	21	2	4	2	8	-	-	13
opp_cand.445-13T	20	15	5	1	1	6	-	-	13
opp_cand.446-13T	20	18	3	4	1	6	-	-	13
opp_cand.447-13T	21	23	3	4	3	7	-	-	13
opp_cand.451-13T	24	18	4	5	2	9	-	-	13
opp_cand_r5.359-13T	21	25	4	3	1	7	-	-	13
opp_cand.134-13V	18	16	2	2	3	2	-	-	13
opp_cand_r2.28-13V	16	17	1	3	1	2	-	-	13
opp_cand_r2.29-13V	14	19	1	3	3	2	-	-	13
opp_cand_r3.183-13V	18	20	5	4	0	2	-	-	13
opp_cand_r5.141-13V	19	21	0	1	2	1	-	-	13
opp_cand_r5.142-13V	19	21	4	4	1	1	-	-	13
opp_cand_r2.34-13X	19	28	3	3	2	0	-	-	13
opp_cand_r2.237-13Z	20	32	1	3	3	2	-	-	13
opp_cand_r2.238-13Z	15	34	0	1	3	1	-	-	13
opp_cand_r2.239-13Z	15	32	2	1	4	1	-	-	13
opp_cand_r2.114-51A	18	4	5	3	4	0	-	-	51
opp_cand_r2.139-51A	12	3	7	5	4	0	-	-	51
opp_cand_r2.67-51A	12	3	6	5	3	0	-	-	51
opp_cand_r3.354-51A	12	7	6	2	0	0	-	-	51
opp_cand_r3.356-51A	10	7	2	3	2	0	-	-	51
opp_cand_r3.283-51AA	12	6	5	4	3	0	-	-	51
opp_cand_r3.302-51AA	12	7	5	3	2	0	-	-	51
opp_cand_r3.357-51AA	11	10	4	5	3	0	-	-	51
opp_cand.271-51AB	5	9	6	5	2	1	-	-	51
opp_cand_r2.136-51AC	12	9	7	5	4	0	-	-	51
opp_cand_r3.340-51AC	11	8	6	6	4	0	-	-	51
opp_cand.258-51AE	17	4	10	5	4	0	-	-	51
opp_cand.274-51AF	13	5	10	6	4	0	-	-	51
opp_cand_r3.299-51AG	8	7	7	5	4	0	-	-	51
opp_cand_r3.300-51AG	7	7	8	4	4	0	-	-	51
opp_cand.272-51AH	5	7	1	1	2	0	-	-	51
opp_cand.273-51AH	4	7	5	5	1	1	-	-	51
opp_cand_r5.259-51AI	11	4	4	2	4	0	-	-	51
opp_cand.270-51AJ	16	6	5	3	2	0	-	-	51
opp_cand.275-51AJ	14	7	4	5	3	0	-	-	51
opp_cand.252-51B	14	3	8	6	3	0	-	-	51
opp_cand.253-51B	18	3	7	4	4	0	-	-	51
opp_cand.254-51B	17	1	7	5	2	0	-	-	51
opp_cand_r2.117-51B	13	3	7	6	4	0	-	-	51
opp_cand_r3.380-51C	12	9	3	3	4	0	-	-	51
opp_cand_r5.60-51C	15	10	3	1	4	0	-	-	51
opp_cand_r5.64-51C	19	6	4	4	4	0	-	-	51
opp_cand_r3.349-51E	14	3	2	5	1	0	-	-	51
opp_cand_r5.46-51E	19	9	5	6	2	0	-	-	51
opp_cand_r2.131-51F	13	11	4	1	4	0	-	-	51
opp_cand_r2.141-51G	12	6	8	6	4	0	-	-	51
opp_cand_r2.140-51H	12	6	7	8	4	1	-	-	51
opp_cand.249-51I	18	7	6	7	3	0	-	-	51
opp_cand_r5.253-51J	9	9	6	6	2	0	-	-	51
opp_cand_r2.120-51K	14	9	11	3	4	0	-	-	51
opp_cand_r5.254-51K	7	6	10	10	4	0	-	-	51

opp_cand_r2.119-51Q	8	12	6	5	4	0	-	-	51
opp_cand_r3.298-51R	15	6	2	2	2	0	-	-	51
opp_cand_r3.381-51S	10	3	3	1	2	0	-	-	51
opp_cand_r5.68-51T	13	6	6	2	1	0	-	-	51
opp_cand.256-51V	17	4	9	3	3	0	-	-	51
opp_cand.257-51X	20	3	10	3	3	0	-	-	51
opp_cand_r2.121-51X	16	4	11	8	4	0	-	-	51
opp_cand_r3.377-51N	7	3	1	2	1	0	-	-	51
opp_cand_r3.329-52A	14	7	10	5	4	0	-	-	52
opp_cand_r3.365-52A	14	9	7	7	4	0	-	-	52
opp_cand_r3.367-52A	15	8	4	9	3	0	-	-	52
opp_cand_r5.262-52A	15	8	7	8	3	0	-	-	52
opp_cand_r3.324-52AG	12	11	7	8	4	0	-	-	52
opp_cand_r3.326-52AG	11	10	4	7	5	0	-	-	52
opp_cand.227-52AH	9	7	5	1	2	0	-	-	52
opp_cand.229-52AH	11	11	4	1	2	0	-	-	52
opp_cand_r3.364-52AI	19	3	3	4	0	0	-	-	52
opp_cand.233-52B	11	3	5	5	4	0	-	-	52
opp_cand.234-52B	15	18	4	5	1	0	-	-	52
opp_cand_r2.124-52B	16	14	5	6	4	0	-	-	52
opp_cand_r2.70-52B	14	10	7	5	2	1	-	-	52
opp_cand_r5.246-52B	12	12	2	5	3	0	-	-	52
opp_cand_r5.252-52B	19	9	12	5	2	1	-	-	52
opp_cand_r5.393-52B	20	10	11	7	4	0	-	-	52
opp_cand_r3.271-52D	17	14	13	2	3	0	-	-	52
opp_cand_r3.290-52D	16	15	12	3	2	0	-	-	52
opp_cand_r3.325-52D	16	15	9	7	0	0	-	-	52
opp_cand_r3.294-52E	19	11	20	3	3	1	-	-	52
opp_cand_r3.316-52E	22	9	17	4	4	0	-	-	52
opp_cand_r3.317-52E	21	9	15	8	4	1	-	-	52
opp_cand_r5.416-52E	21	10	14	6	3	0	-	-	52
opp_cand_r3.293-52I	16	7	1	3	2	0	-	-	52
opp_cand_r5.31-52I	17	8	2	3	2	0	-	-	52
opp_cand_r5.39-52I	18	6	1	1	2	0	-	-	52
opp_cand_r5.40-52I	17	8	1	1	2	0	-	-	52
opp_cand_r5.41-52I	16	9	0	2	2	0	-	-	52
opp_cand_r5.42-52I	17	5	0	1	1	0	-	-	52
opp_cand_r3.322-52J	16	8	9	3	4	0	-	-	52
opp_cand_r3.327-52J	14	7	12	2	4	1	-	-	52
opp_cand_r5.275-52J	16	8	13	5	4	0	-	-	52
opp_cand_r5.35-52K	9	15	8	3	2	0	-	-	52
opp_cand_r5.36-52K	12	12	7	6	1	0	-	-	52
opp_cand_r2.132-52L	15	7	9	5	1	0	-	-	52
opp_cand_r5.34-52M	8	6	7	5	2	0	-	-	52
opp_cand.226-52P	14	10	8	7	1	1	-	-	52
opp_cand_r5.37-52P	13	8	4	5	3	0	-	-	52
opp_cand_r3.304-52R	12	8	10	6	2	0	-	-	52
opp_cand_r2.125-52S	17	14	5	6	2	0	-	-	52
opp_cand_r3.342-52S	16	5	10	7	2	0	-	-	52
opp_cand_r3.361-52S	11	6	10	5	1	1	-	-	52
opp_cand_r3.362-52S	12	7	8	7	1	0	-	-	52
opp_cand_r3.363-52S	12	8	8	4	1	1	-	-	52
opp_cand_r5.266-52S	14	9	10	6	0	0	-	-	52
opp_cand_r5.270-52S	14	4	7	8	4	0	-	-	52
opp_cand_r5.272-52S	11	9	8	4	2	1	-	-	52
opp_cand_r5.245-52U	10	6	2	3	2	0	-	-	52

opp_cand.245-52V	13	6	9	1	4	0	-	-	52
opp_cand_r5.396-52X	17	8	5	4	1	0	-	-	52
opp_cand.240-52Z	18	7	8	2	3	0	-	-	52
opp_cand.241-52Z	17	7	5	2	3	0	-	-	52
opp_cand.259-52Z	16	8	10	4	1	2	-	-	52
opp_cand.260-52Z	16	10	10	4	1	2	-	-	52
opp_cand_r2.111-52Z	14	7	10	2	3	0	-	-	52
opp_cand_r5.395-52AM	15	6	2	6	0	0	-	-	52
opp_cand_r2.90-55B	14	4	0	2	0	0	-	-	55
opp_cand_r3.332-55B	15	4	0	2	1	1	-	-	55
opp_cand_r3.333-55B	13	2	0	2	2	0	-	-	55
opp_cand_r5.181-55B	14	2	0	2	2	0	-	-	55
opp_cand_r5.261-56A	18	3	4	8	2	0	-	-	56
opp_cand_r5.403-56A	14	2	4	7	4	0	-	-	56
opp_cand.220-56B	20	7	2	3	1	0	-	-	56
opp_cand_r2.102-56B	16	2	3	6	1	0	-	-	56
opp_cand_r2.103-56B	16	2	3	6	1	0	-	-	56
opp_cand_r2.104-56B	12	4	2	6	1	0	-	-	56
