# Supplementary material for: Common peptides shed light on evolution of Olfactory Receptors
Source: BMC Evol Biol. 2009 May 5;9:91. doi: 10.1186/1471-2148-9-91 (PMC2681464; doi:10.1186/1471-2148-9-91)
Supplement: Additional file 7 — Dog ORs CP numbers and cluster assignment. Number of CPs from each ancestor occurring in each Dog OR and cluster assignment for each Dog OR. [file 1471-2148-9-91-S7.pdf]

Legend

A	Number of A1 CPs
B	Number of A2 novel CPs
C	Number of A3 novel CPs
D	Number of A5 novel CPs
E	Number of A6 novel CPs
F	Number of A7 novel CPs
G	Number of dog novel CPs
H	Cluster number A4 novel CPs
I	Cluster number A5 novel CPs
J	Cluster number A6 novel CPs
K	Family

Name	A	B	C	D	E	F	G	H	I	J	K
cOR7C37	23	32	5	9	10	8	0	1	6	1	7
cOR7C47	20	30	7	9	10	6	0	1	6	1	7
cOR7C55	21	27	3	9	5	6	0	1	6	1	7
cOR6C39	23	19	2	8	7	3	0	1	7	-	6
cOR6C44	7	9	1	5	10	1	1	1	7	-	6
cOR6C47	22	22	2	14	7	2	4	1	7	-	6
cOR8B18	21	32	6	1	5	1	0	1	7	-	8
cOR6C4	22	18	5	8	5	0	0	1	7	-	6
cOR6C45P	23	23	5	11	6	0	0	1	7	-	6
cOR6C55P	15	20	3	8	15	1	1	1	7	-	6
cOR6C7	17	26	1	7	6	0	1	1	7	-	6
cOR6C81	23	24	4	12	7	3	0	1	7	-	6
cOR6C50	22	26	3	8	8	0	1	1	7	-	6
cOR6C63	22	24	5	15	5	0	0	1	7	-	6
cOR6C48P	20	19	0	12	5	2	5	1	7	-	6
cOR6C91P	19	20	1	11	5	2	5	1	7	-	6
cOR6C92P	18	19	1	11	6	2	5	1	7	-	6
cOR6C10P	18	19	1	11	6	2	5	1	7	-	6
cOR6C9	21	12	6	7	12	1	0	1	7	-	6
cOR52N9	13	6	8	6	6	0	0	1	12	-	52
cOR6C84P	20	21	5	8	4	2	0	1	-	-	6
cOR6C23P	19	19	4	10	4	1	1	1	-	-	6
cOR6C40P	27	22	4	9	3	1	1	1	-	-	6
cOR6C28P	18	12	1	6	3	0	1	1	-	-	6
cOR6C97P	25	23	4	9	3	1	1	1	-	-	6
cOR6C54P	23	21	5	5	3	1	1	1	-	-	6
cOR5H11	11	19	7	2	7	2	1	2	1	-	5
cOR11G1	17	29	5	2	5	3	1	2	1	-	11
cOR56A15	19	2	3	9	7	0	0	2	4	-	56
cOR8V4	18	19	7	8	6	0	0	2	5	-	8
cOR8V2	17	19	8	9	6	0	0	2	5	-	8
cOR1E11	21	31	7	4	7	3	0	2	5	-	1
cOR8V11	16	21	9	4	7	0	0	2	5	-	8
cOR7C17	20	30	5	9	9	5	1	2	6	1	7
cOR7C23	17	31	4	6	12	7	0	2	6	1	7
cOR7C27	20	27	2	7	10	7	0	2	6	1	7
cOR7C32	21	29	5	9	7	8	1	2	6	1	7
cOR7C30	22	24	5	7	12	10	0	2	6	1	7

cOR7C18	22	28	5	7	7	8	1	2	6	1	7
cOR7C36	17	24	5	9	12	6	0	2	6	1	7
cOR7C41P	21	31	5	6	6	7	0	2	6	1	7
cOR7C10P	23	22	6	5	6	7	1	2	6	1	7
cOR7A26	14	24	3	8	7	2	0	2	6	-	7
cOR7C48	17	30	5	9	7	3	1	2	6	-	7
cOR7C8P	21	30	3	8	5	1	0	2	6	-	7
cOR	17	24	4	10	5	2	1	2	6	-	0
cOR8G8	18	34	6	5	10	0	0	2	7	-	8
cOR6C35	17	17	3	3	11	1	0	2	7	-	6
cOR2T23	17	17	1	3	6	0	0	2	7	-	2
cOR6C11	22	24	4	8	7	2	0	2	7	-	6
cOR52H11	14	12	9	5	7	2	1	2	8	-	52
cOR5B27	15	30	5	3	8	2	0	2	9	-	5
cOR6Z1	15	20	4	0	5	0	0	2	10	-	6
cOR9S3P	16	32	10	2	9	5	3	2	11	3	9
cOR10D4	18	21	1	2	6	0	0	2	11	-	10
cOR13D8	24	36	1	2	5	1	0	2	11	-	13
cOR52E16	17	9	14	5	6	2	0	2	12	-	52
cOR51C4	14	11	6	4	10	0	0	2	12	-	51
cOR52E10	19	8	15	6	9	1	0	2	12	-	52
cOR52N6	10	6	8	8	6	0	0	2	12	-	52
cOR7C25	19	26	3	9	3	6	0	2	-	1	7
cOR7D8	24	35	3	6	0	0	0	2	-	-	7
cOR1AB3	31	29	2	6	3	0	0	2	-	-	1
cOR7H3	27	26	3	7	4	1	0	2	-	-	7
cOR7G7	27	23	3	8	2	4	0	2	-	-	7
cOR7C15	24	26	1	8	4	2	0	2	-	-	7
cOR1L6	17	30	5	6	0	0	0	2	-	-	1
cOR7D7	20	32	4	6	0	1	0	2	-	-	7
cOR7G5	22	19	1	6	1	3	0	2	-	-	7
cOR1L10P	18	28	5	7	0	0	0	2	-	-	1
cOR1AB2P	32	29	2	6	3	0	0	2	-	-	1
cOR7H6P	20	29	4	7	4	2	0	2	-	-	7
cOR51X4	15	5	12	5	4	0	1	3	-	-	51
cOR51V8	18	6	8	5	6	0	0	3	-	-	51
cOR51X5	17	4	11	5	4	0	0	3	-	-	51
cOR51X6	17	4	11	5	4	0	0	3	-	-	51
cOR51V6	16	5	8	5	6	0	0	3	-	-	51
cOR51X3P	12	6	10	5	4	0	0	3	-	-	51
cOR56A25	18	2	5	11	5	0	0	4	2	-	56
cOR56A20	14	3	4	7	6	0	1	4	2	-	56
cOR56A13	14	2	3	7	5	0	1	4	2	-	56
cOR56A21P	14	2	4	8	6	0	1	4	2	-	56
cOR56A11	16	2	5	10	6	0	0	4	4	-	56
cOR56A12P	16	2	2	8	5	0	1	4	4	-	56
cOR56A16	13	2	3	9	6	0	1	4	4	-	56
cOR56A17	15	2	2	9	6	0	1	4	4	-	56
cOR2K2	23	23	3	1	5	0	0	4	6	-	2
cOR7C7	21	34	3	6	6	2	0	4	6	-	7
cOR52N10	8	8	6	7	6	0	0	4	7	-	52
cOR6C88	21	13	6	7	12	1	0	4	7	-	6

cOR8F2	17	36	7	3	15	1	1	4	7	-	8
cOR5B34	18	34	5	2	10	1	0	4	9	-	5
cOR51A21	9	9	5	2	6	0	0	4	12	-	51
cOR52E8	16	9	15	6	9	2	0	4	12	-	52
cOR10H18	16	12	0	5	4	3	2	4	-	-	10
cOR56B12	14	6	2	6	2	1	1	4	-	-	56
cOR10H6	16	24	0	5	1	1	0	4	-	-	10
cOR56B10	14	5	3	6	1	2	2	4	-	-	56
cOR56B5	13	4	3	5	1	2	2	4	-	-	56
cOR56B13	11	4	1	5	1	0	1	4	-	-	56
cOR10H21	16	26	0	5	1	1	0	4	-	-	10
cOR6C13	20	17	0	6	6	1	2	5	7	-	6
cOR5P6	20	19	2	9	4	1	0	5	-	-	5
cOR12E1	23	23	5	5	2	0	0	5	-	-	12
cOR5H16	13	22	6	4	8	1	0	6	1	-	5
cOR8V6	17	23	10	6	7	0	0	6	5	-	8
cOR13C27	25	33	1	3	6	0	0	6	5	-	13
cOR13C16	27	32	2	3	5	0	0	6	5	-	13
cOR7C34	20	32	3	8	5	6	1	6	6	1	7
cOR5K7	17	16	1	1	5	0	0	6	7	-	5
cOR52H9	18	9	11	5	6	1	0	6	8	-	52
cOR9S16	16	28	6	1	10	3	3	6	11	-	9
cOR52N12	9	4	6	7	7	0	0	6	12	-	52
cOR52E25	22	9	13	4	6	0	0	6	12	-	52
cOR8U10	24	38	7	7	2	0	0	6	-	-	8
cOR2W9	15	30	3	5	2	0	0	6	-	-	2
cOR2W11	17	25	5	6	3	0	0	6	-	-	2
cOR4C33P	24	17	8	11	5	5	3	7	1	-	4
cOR4C20P	23	17	9	10	5	4	1	7	1	-	4
cOR4C61P	24	16	6	10	5	5	3	7	1	-	4
cOR4C44P	24	17	6	10	5	5	3	7	1	-	4
cOR56A18	13	3	2	8	5	0	1	7	4	-	56
cOR5W6	16	27	9	5	7	1	1	7	5	-	5
cOR8C6	19	28	4	4	10	2	0	7	7	-	8
cOR6C93	22	15	2	5	11	2	1	7	7	-	6
cOR4A34	23	18	4	8	2	5	1	7	-	2	4
cOR4A53	22	13	6	8	2	5	1	7	-	2	4
cOR13N1	29	34	1	5	2	3	1	7	-	-	13
cOR2D7	24	29	1	6	4	3	1	7	-	-	2
cOR4C27	24	15	4	9	2	2	1	7	-	-	4
cOR4A29	25	22	6	9	2	3	1	7	-	-	4
cOR4C29	25	16	6	11	3	0	0	7	-	-	4
cOR2D5	26	25	2	6	2	2	1	7	-	-	2
cOR4C31	24	14	9	11	2	3	1	7	-	-	4
cOR4C28P	28	10	7	10	2	2	1	7	-	-	4
cOR4C30P	27	17	7	10	2	1	1	7	-	-	4
cOR4C32P	24	16	8	10	3	3	1	7	-	-	4
cOR4C64P	22	16	4	9	4	4	1	7	-	-	4
cOR4C11	24	14	5	12	3	4	2	7	-	-	4
cOR4C42P	25	16	9	8	2	3	0	7	-	-	4
cOR4C37	23	17	7	9	5	4	2	8	1	-	4
cOR2T37	21	21	2	6	5	1	1	8	5	-	2

cOR8B1	19	34	6	1	15	0	0	8	7	-	8
cOR8B3	15	30	5	1	17	0	0	8	7	-	8
cOR10J22	17	20	4	0	5	1	0	8	7	-	10
cOR5AC3	14	23	4	1	6	2	0	8	7	-	5
cOR8B8	21	32	8	2	12	1	0	8	7	-	8
cOR52B6	21	10	7	2	5	1	0	8	8	-	52
cOR52H4	11	9	7	7	6	0	2	8	8	-	52
cOR5B35	18	32	2	1	10	2	0	8	9	-	5
cOR52E24	15	8	15	6	6	0	0	8	12	-	52
cOR2T36	13	22	0	8	4	1	1	8	-	-	2
cOR2T18	17	27	0	6	5	0	0	8	-	-	2
cOR2T13	18	21	3	6	1	3	2	8	-	-	2
cOR2T16	18	12	2	6	0	2	0	8	-	-	2
cOR4Z2	20	22	7	6	1	1	0	8	-	-	4
cOR4Z1P	19	17	3	5	0	3	0	8	-	-	4
cOR56A8	16	2	3	9	7	0	1	9	4	-	56
cOR56A4	19	2	3	8	6	0	0	9	4	-	56
cOR7C35	23	29	2	7	9	7	0	9	6	1	7
cOR7C16	24	22	5	6	13	8	0	9	6	1	7
cOR7C6	20	29	6	9	10	7	0	9	6	1	7
cOR7C21	22	33	5	11	9	9	0	9	6	1	7
cOR7C24	23	24	6	8	8	10	0	9	6	1	7
cOR7C38	18	31	4	5	9	4	0	9	6	-	7
cOR71	18	31	5	10	5	2	1	9	6	-	71
cOR5K5	17	16	1	1	5	0	0	9	7	-	5
cOR52H3	16	12	9	7	11	1	1	9	8	-	52
cOR52H2	13	13	8	5	7	2	1	9	8	-	52
cOR13C19	25	35	0	4	6	1	0	9	8	-	13
cOR52B2P	14	10	8	7	6	0	0	9	8	-	52
cOR52H12P	15	12	8	7	11	1	1	9	8	-	52
cOR52H1	14	12	9	7	11	1	1	9	8	-	52
cOR5B31	18	31	7	2	7	2	0	9	9	-	5
cOR9S20	19	23	6	2	10	3	3	9	11	-	9
cOR10D9	24	17	2	1	7	1	0	9	11	-	10
cOR6K8	21	15	0	1	5	0	0	9	11	-	6
cOR9S5	17	28	7	2	12	3	4	9	11	-	9
cOR51I5	15	9	6	7	5	1	0	9	12	-	51
cOR51B7	16	4	8	5	6	0	0	9	12	-	51
cOR51Q1	10	12	5	5	6	0	0	9	12	-	51
cOR52E15	21	10	15	7	6	0	0	9	12	-	52
cOR51Q3	9	10	5	7	8	0	0	9	12	-	51
cOR52N7	11	4	6	5	5	0	0	9	12	-	52
cOR52N2	10	6	8	7	7	0	0	9	12	-	52
cOR52AB4	15	8	12	7	6	0	0	9	12	-	52
cOR51L5	15	7	6	7	7	0	0	9	12	-	51
cOR51H3	14	7	9	7	5	0	0	9	12	-	51
cOR52J7	12	11	12	5	5	0	0	9	12	-	52
cOR51G1	13	9	11	6	6	1	0	9	12	-	51
cOR52E20	18	10	10	6	6	0	0	9	12	-	52
cOR51AF1	10	6	8	5	5	1	0	9	12	-	51
cOR51P4	10	6	7	7	7	0	0	9	12	-	51
cOR51AF2	10	6	8	5	5	1	0	9	12	-	51

cOR52E2	18	10	13	5	6	0	0	9	12	-	52
cOR51L2P	15	5	8	9	5	0	0	9	12	-	51
cOR51L3	15	7	6	5	7	0	0	9	12	-	51
cOR52E21	16	9	13	5	2	0	0	9	-	-	52
cOR51I2	15	9	4	7	4	1	0	9	-	-	51
cOR52L4	12	7	9	5	2	0	0	9	-	-	52
cOR51A24	16	6	4	7	0	0	0	9	-	-	51
cOR52A15	12	4	9	9	2	1	0	9	-	-	52
cOR52N8	10	7	9	8	4	0	0	9	-	-	52
cOR52A14	12	7	9	8	2	1	1	9	-	-	52
cOR52P4	14	10	4	5	0	0	0	9	-	-	52
cOR52B10	18	10	5	8	1	0	0	9	-	-	52
cOR52AE1	7	9	5	8	1	2	0	9	-	-	52
cOR51K2	14	6	7	8	2	0	0	9	-	-	51
cOR13C17P	20	33	0	5	1	1	0	9	-	-	13
cOR51Q2P	9	8	4	5	3	2	0	9	-	-	51
cOR52A13P	14	7	3	8	1	1	0	9	-	-	52
cOR51A22	20	5	9	6	2	0	0	9	-	-	51
cOR52AF1	14	8	6	5	2	0	0	9	-	-	52
cOR51AA2	12	8	6	5	4	0	0	9	-	-	51
cOR52E19	16	12	14	5	3	0	0	9	-	-	52
cOR52A18	15	6	6	10	2	1	2	9	-	-	52
cOR52A19	10	7	6	6	2	1	2	9	-	-	52
cOR51H5	8	7	11	7	4	0	0	9	-	-	51
cOR52S6	15	6	10	8	3	1	0	9	-	-	52
cOR52S2	11	7	7	5	3	0	1	9	-	-	52
cOR52AF2	14	8	6	5	2	0	0	9	-	-	52
cOR52A20	15	8	9	11	2	1	2	9	-	-	52
cOR52AB2	15	8	6	7	3	0	0	9	-	-	52
cOR52X3	16	4	8	7	3	1	0	9	-	-	52
cOR52AB3	14	6	9	8	1	0	0	9	-	-	52
cOR52J5	13	11	13	7	4	0	0	9	-	-	52
cOR52A21	15	6	6	10	2	1	2	9	-	-	52
cOR52AG1P	9	8	5	8	1	2	0	9	-	-	52
cOR52S3P	13	8	10	8	0	0	0	9	-	-	52
cOR11H8	23	23	6	0	6	1	0	10	1	-	11
cOR2T14	19	22	2	7	5	1	1	10	5	-	2
cOR6C38	17	23	4	6	16	1	1	10	7	-	6
cOR6C56P	21	18	3	8	13	1	1	10	7	-	6
cOR6C24P	18	16	2	7	14	1	1	10	7	-	6
cOR13P1	16	19	3	9	13	2	0	10	7	-	13
cOR6C85	17	21	4	6	14	1	1	10	7	-	6
cOR6C46P	21	19	3	8	15	1	1	10	7	-	6
cOR6C82	20	18	4	6	4	0	0	10	-	-	6
cOR5M16	27	31	2	5	3	1	0	10	-	-	5
cOR7C20	21	34	4	5	11	9	0	11	6	1	7
cOR7C22	23	26	5	9	8	9	1	11	6	1	7
cOR5AR1	18	27	9	3	6	0	0	11	7	-	5
cOR8D6	18	33	3	2	6	0	1	11	7	-	8
cOR8B21	18	32	4	2	14	0	0	11	7	-	8
cOR6C37	21	23	3	9	15	2	1	11	7	-	6
cOR6C62	17	10	0	5	8	2	1	11	7	-	6

cOR6C96P	19	20	1	11	5	2	5	11	7	-	6
cOR52H5	12	15	8	5	8	2	2	11	8	-	52
cOR6Z3	15	19	7	0	6	1	0	11	10	-	6
cOR10AI1	21	32	1	1	5	0	0	11	11	-	10
cOR4C52	20	15	7	7	0	1	0	11	-	-	4
cOR4C53	19	14	6	8	2	1	0	11	-	-	4
cOR4P11	25	15	5	8	0	2	0	11	-	-	4
cOR4C54	25	22	7	11	0	1	0	11	-	-	4
cOR4F34	18	15	4	7	4	0	0	11	-	-	4
cOR4A51	30	23	8	7	3	2	0	11	-	-	4
cOR4C58	27	25	4	12	0	1	0	11	-	-	4
cOR4C41	24	22	8	12	3	0	0	11	-	-	4
cOR4Y4	21	20	6	5	0	1	1	11	-	-	4
cOR4A26	31	23	8	6	3	2	0	11	-	-	4
cOR4C1	28	16	4	7	2	0	0	11	-	-	4
cOR4P7	23	20	4	5	0	1	0	11	-	-	4
cOR4C59	17	30	4	7	0	0	0	11	-	-	4
cOR4C24	23	18	8	11	3	0	0	11	-	-	4
cOR4A4	25	24	3	6	1	4	1	11	-	-	4
cOR4S5	23	19	4	5	0	0	0	11	-	-	4
cOR4V3P	19	17	3	5	0	0	2	11	-	-	4
cOR4C60P	28	17	7	14	1	1	0	11	-	-	4
cOR4C63P	28	19	6	15	1	1	0	11	-	-	4
cOR4X5P	18	25	2	5	0	0	0	11	-	-	4
cOR4C43P	28	23	7	12	0	0	0	11	-	-	4
cOR4C39	26	20	6	14	0	0	0	11	-	-	4
cOR4A27P	15	12	2	6	3	1	2	11	-	-	4
cOR4A28	21	20	5	7	1	2	1	11	-	-	4
cOR4A52	24	23	6	7	2	2	1	11	-	-	4
cOR4C55	26	22	6	14	0	0	0	11	-	-	4
cOR4C57	22	11	6	7	1	1	0	11	-	-	4
cOR4X7	24	13	4	8	1	2	0	11	-	-	4
cOR4X3	16	8	5	7	1	1	0	11	-	-	4
cOR8V8P	20	20	8	5	7	0	0	12	5	-	8
cOR7C39	19	33	3	8	7	4	1	12	6	-	7
cOR8F4	17	40	6	4	12	0	0	12	7	-	8
cOR6C17	21	12	2	3	5	0	0	12	7	-	6
cOR10T4	15	24	5	1	5	1	0	12	7	-	10
cOR9S6	15	26	9	1	10	5	3	12	11	3	9
cOR52E4	19	9	14	9	7	0	0	12	12	-	52
cOR5W8	14	30	7	5	3	1	0	12	-	-	5
cOR8K7	21	27	7	6	2	0	0	12	-	-	8
cOR8K1	22	27	2	6	4	1	0	12	-	-	8
cOR11G5	15	26	5	3	5	0	0	-	1	-	11
cOR11H9	17	27	3	3	6	1	0	-	1	-	11
cOR5H10	13	19	6	3	8	1	0	-	1	-	5
cOR8W1	16	28	8	4	6	1	0	-	5	-	8
cOR2T21	18	27	0	4	7	1	0	-	5	-	2
cOR2T38	19	24	1	7	8	0	0	-	5	-	2
cOR5W9P	15	26	7	4	6	2	1	-	5	-	5
cOR2T19P	21	16	2	4	5	1	1	-	5	-	2
cOR5W7	9	21	6	4	6	2	1	-	5	-	5

cOR7C28	19	27	5	6	10	5	0	-	6	1	7
cOR2AD2	15	34	0	4	5	0	0	-	6	-	2
cOR8D7	15	31	1	2	12	0	0	-	7	-	8
cOR13A2	20	21	0	2	5	0	0	-	7	-	13
cOR5K8	17	16	1	1	5	0	0	-	7	-	5
cOR6C27	22	19	3	3	5	1	0	-	7	-	6
cOR8C7	16	20	0	3	12	1	0	-	7	-	8
cOR8B20	17	33	5	2	15	0	0	-	7	-	8
cOR10J19	11	19	2	1	5	0	0	-	7	-	10
cOR10H10	16	15	0	5	6	4	2	-	7	-	10
cOR6C42	19	16	3	5	5	1	0	-	7	-	6
cOR8D4	15	30	4	3	8	0	0	-	7	-	8
cOR10T3	14	23	1	0	7	0	0	-	7	-	10
cOR8D2	16	31	7	6	9	1	0	-	7	-	8
cOR8A1	16	35	5	4	9	0	0	-	7	-	8
cOR10H7	16	12	0	4	6	3	1	-	7	-	10
cOR8B22P	12	28	3	1	14	0	0	-	7	-	8
cOR8B23P	20	26	5	2	10	2	0	-	7	-	8
cOR8C5P	15	30	1	1	11	1	0	-	7	-	8
cOR6C22P	16	15	3	2	9	1	1	-	7	-	6
cOR8B15P	18	35	4	2	16	0	0	-	7	-	8
cOR6C41P	12	12	1	4	6	1	1	-	7	-	6
cOR8B14	19	30	5	3	18	1	0	-	7	-	8
cOR8B19P	17	33	4	2	14	0	0	-	7	-	8
cOR6C94P	14	18	3	3	8	0	0	-	7	-	6
cOR8B16P	19	36	5	2	16	1	0	-	7	-	8
cOR6C89	20	14	2	3	5	0	0	-	7	-	6
cOR6C90	14	11	3	3	8	2	1	-	7	-	6
cOR6C87P	17	14	3	3	10	1	0	-	7	-	6
cOR8F3	12	33	6	3	15	1	1	-	7	-	8
cOR13C22	26	31	0	3	9	2	0	-	8	-	13
cOR13C25	24	26	2	1	7	1	0	-	8	-	13
cOR13C14	26	34	0	4	6	2	0	-	8	-	13
cOR52H7	17	8	10	4	5	0	1	-	8	-	52
cOR13C28P	25	36	1	4	5	2	0	-	8	-	13
cOR5B24	14	33	3	2	8	2	0	-	9	-	5
cOR5B25	11	33	4	4	9	1	0	-	9	-	5
cOR5R2	18	42	3	1	5	0	0	-	9	-	5
cOR5B26	17	27	7	2	13	1	1	-	9	-	5
cOR5B33P	14	33	3	2	7	1	0	-	9	-	5
cOR5B30P	16	29	5	3	9	2	0	-	9	-	5
cOR6Z2	14	14	6	0	6	2	0	-	10	-	6
cOR10J21	19	23	4	1	6	0	0	-	10	-	10
cOR6AF1	15	16	7	0	6	1	0	-	10	-	6
cOR10G13	13	22	0	3	6	6	0	-	11	2	10
cOR10G14P	14	22	0	4	5	6	0	-	11	2	10
cOR10G15P	10	21	0	3	6	5	0	-	11	2	10
cOR10G12P	15	23	0	4	6	7	0	-	11	2	10
cOR9S7	20	23	9	3	9	5	3	-	11	3	9
cOR9S25P	16	27	11	2	11	5	3	-	11	3	9
cOR9S21	16	24	11	3	11	5	3	-	11	3	9
cOR9S10	16	29	9	3	10	5	4	-	11	3	9

cOR10D1	24	11	2	1	7	1	0	-	11	-	10
cOR9R5	18	26	3	2	5	0	0	-	11	-	9
cOR10D5	19	21	0	1	5	0	0	-	11	-	10
cOR9R3P	19	31	4	0	5	0	0	-	11	-	9
cOR9S11P	19	25	7	2	10	4	4	-	11	-	9
cOR9S12P	15	30	6	2	9	3	4	-	11	-	9
cOR9S26P	16	28	8	3	10	4	4	-	11	-	9
cOR9S19P	17	27	5	1	10	4	2	-	11	-	9
cOR9R6	19	26	3	1	6	0	0	-	11	-	9
cOR13D5	21	35	1	2	5	1	0	-	11	-	13
cOR51F6	16	9	6	3	5	0	0	-	12	-	51
cOR51F7	15	9	5	3	9	0	0	-	12	-	51
cOR52M1	10	6	7	4	5	0	0	-	12	-	52
cOR51C5	13	15	4	2	7	0	0	-	12	-	51
cOR52E18P	19	6	14	4	5	1	0	-	12	-	52
cOR51A23	9	5	6	4	5	0	0	-	12	-	51
cOR51H4	12	4	8	4	6	0	0	-	12	-	51
cOR52E23	18	8	17	3	6	0	0	-	12	-	52
cOR51F2	17	13	6	2	5	0	0	-	12	-	51
cOR51A26	11	11	8	4	5	0	0	-	12	-	51
cOR52Z6	19	7	9	2	5	0	1	-	12	-	52
cOR52Z5	19	7	9	2	5	0	1	-	12	-	52
cOR51V4	11	11	8	4	5	0	0	-	12	-	51
cOR51A14P	10	7	7	4	6	0	0	-	12	-	51
cOR52E26P	14	10	9	2	7	0	0	-	12	-	52
cOR52J6	11	12	12	4	5	0	0	-	12	-	52
cOR51V2P	10	10	6	3	6	0	0	-	12	-	51
cOR52E11P	14	10	9	2	7	0	0	-	12	-	52
cOR7E152	22	32	4	2	4	5	0	-	-	1	7
cOR7G8	24	27	3	6	0	5	0	-	-	1	7
cOR7G6	27	27	4	4	1	5	0	-	-	1	7
cOR7G12	21	25	6	5	3	5	0	-	-	1	7
cOR7G9	21	23	0	8	2	5	0	-	-	1	7
cOR7G14	23	25	1	5	0	5	0	-	-	1	7
cOR4A55	22	24	7	6	1	5	1	-	-	2	4
cOR10H16	17	16	0	4	3	2	1	-	-	-	10
cOR2D11	23	26	1	3	3	2	0	-	-	-	2
cOR9G10	14	33	5	0	3	0	0	-	-	-	9
cOR13J2	21	27	3	4	2	1	0	-	-	-	13
cOR2L20	15	13	4	1	3	2	0	-	-	-	2
cOR2L21	12	17	4	2	1	2	0	-	-	-	2
cOR4N7	17	16	6	3	0	0	0	-	-	-	4
cOR52U4	13	7	3	3	2	0	0	-	-	-	52
cOR52K6	17	13	8	4	3	0	0	-	-	-	52
cOR10A16	12	20	2	3	2	4	0	-	-	-	10
cOR7E153	25	27	2	4	2	4	0	-	-	-	7
cOR6S2	20	23	2	0	2	0	0	-	-	-	6
cOR6B9	17	26	2	0	0	0	0	-	-	-	6
cOR10C2	23	19	4	0	1	0	0	-	-	-	10
cOR5M19	20	26	2	2	0	0	0	-	-	-	5
cOR5AN6	22	30	5	0	1	0	0	-	-	-	5
cOR9K7	19	25	2	0	0	0	1	-	-	-	9

cOR8S18	15	27	2	1	0	1	1	-	-	-	8
cOR10AA2	14	12	2	2	1	0	0	-	-	-	10
cOR4Q7	22	25	7	2	0	0	1	-	-	-	4
cOR5AN7	17	33	11	2	2	0	0	-	-	-	5
cOR9K8	21	30	8	0	1	0	0	-	-	-	9
cOR6N3	19	20	0	1	1	1	0	-	-	-	6
cOR2Y2	11	31	2	3	1	0	0	-	-	-	2
cOR4V4	20	15	4	1	3	1	1	-	-	-	4
cOR5T8	9	30	11	2	3	1	0	-	-	-	5
cOR5AQ2	13	30	2	1	1	0	0	-	-	-	5
cOR4Q5	19	17	9	2	0	0	1	-	-	-	4
cOR13M4	25	23	2	1	2	1	0	-	-	-	13
cOR6K10	17	14	0	0	3	3	0	-	-	-	6
cOR7R1	20	24	5	2	1	0	0	-	-	-	7
cOR12E5	25	25	5	2	4	0	1	-	-	-	12
cOR9Q4	13	26	1	1	0	0	0	-	-	-	9
cOR5AK8	19	24	6	1	3	1	0	-	-	-	5
cOR5L4	26	27	9	3	3	0	0	-	-	-	5
cOR13C12	25	34	2	4	4	2	0	-	-	-	13
cOR8S14	22	20	4	2	0	4	1	-	-	-	8
cOR10D10	20	20	0	1	4	0	0	-	-	-	10
cOR2D12	24	17	2	4	1	2	1	-	-	-	2
cOR5A2	14	41	5	1	0	0	0	-	-	-	5
cOR6C83	20	18	3	4	4	1	0	-	-	-	6
cOR2A43	28	27	2	1	2	1	0	-	-	-	2
cOR9Q3	16	25	0	0	0	0	0	-	-	-	9
cOR1AE1	21	17	2	2	3	1	0	-	-	-	1
cOR2Q1	16	20	3	0	0	0	0	-	-	-	2
cOR1K2	17	26	2	3	0	1	0	-	-	-	1
cOR1X2	24	23	3	2	0	0	0	-	-	-	1
cOR4K15	17	17	7	3	2	1	0	-	-	-	4
cOR13C13	27	37	2	1	4	1	1	-	-	-	13
cOR6M5	18	29	1	0	3	0	0	-	-	-	6
cOR4K27	18	13	7	1	1	0	0	-	-	-	4
cOR2M9	16	10	0	1	0	2	0	-	-	-	2
cOR4B3	23	22	6	1	1	0	0	-	-	-	4
cOR13G1	12	20	1	0	2	1	0	-	-	-	13
cOR11L2	16	30	1	0	1	0	0	-	-	-	11
cOR5A5	18	35	6	1	1	1	0	-	-	-	5
cOR6B10	14	27	2	0	3	0	0	-	-	-	6
cOR5D20	17	22	4	1	4	1	0	-	-	-	5
cOR52M5	13	5	9	4	3	0	0	-	-	-	52
cOR1M1	21	25	1	3	2	0	0	-	-	-	1
cOR11H7	19	23	3	1	3	1	0	-	-	-	11
cOR6D6	10	10	1	2	0	0	1	-	-	-	6
cOR8J5	18	28	6	4	3	1	0	-	-	-	8
cOR10A5	17	27	6	2	3	0	1	-	-	-	10
cOR11I3	19	30	1	0	0	0	0	-	-	-	11
cOR5D21	21	36	7	1	3	0	0	-	-	-	5
cOR6W1	18	20	0	0	1	0	0	-	-	-	6
cOR52W2	13	6	7	2	4	0	0	-	-	-	52
cOR6P1	14	28	1	0	1	0	0	-	-	-	6

cOR2B7	16	23	4	2	1	0	0	-	-	-	2
cOR1D11	15	8	2	4	0	0	0	-	-	-	1
cOR2AV2	19	18	1	0	0	0	0	-	-	-	2
cOR5D14	20	26	9	3	1	0	1	-	-	-	5
cOR13P3	17	26	3	2	2	2	0	-	-	-	13
cOR5M12	24	23	1	4	0	0	0	-	-	-	5
cOR4D2	13	17	6	7	1	0	0	-	-	-	4
cOR11G7	17	29	5	2	2	2	1	-	-	-	11
cOR6K2	20	15	0	0	1	0	0	-	-	-	6
cOR2W12	16	24	4	7	1	0	0	-	-	-	2
cOR7H5	25	38	1	14	4	2	1	-	-	-	7
cOR1AD1	14	28	4	1	4	0	0	-	-	-	1
cOR6M6	18	29	0	0	4	0	0	-	-	-	6
cOR8S12	14	23	0	2	0	3	0	-	-	-	8
cOR2C1	14	19	6	4	4	0	0	-	-	-	2
cOR4L1	18	18	8	3	5	0	0	-	-	-	4
cOR6AA1	12	28	1	1	2	0	0	-	-	-	6
cOR52P1	16	10	8	7	1	0	0	-	-	-	52
cOR13E3	20	19	3	4	1	1	1	-	-	-	13
cOR10R4	13	26	4	1	3	0	0	-	-	-	10
cOR52V2	15	5	7	2	0	0	0	-	-	-	52
cOR5AK7	20	26	3	2	3	2	0	-	-	-	5
cOR2AT7	16	17	1	0	2	0	0	-	-	-	2
cOR10Z2	12	21	3	0	4	0	0	-	-	-	10
cOR5M8	23	30	7	7	0	0	0	-	-	-	5
cOR2AG7	18	16	0	3	3	6	0	-	-	-	2
cOR7G10	26	32	2	11	2	4	1	-	-	-	7
cOR5G8	21	32	6	3	1	0	1	-	-	-	5
cOR10A17	19	30	5	3	3	1	0	-	-	-	10
cOR5C1	16	26	6	5	3	0	0	-	-	-	5
cOR6D5	15	11	2	1	1	0	0	-	-	-	6
cOR51A25	11	9	4	3	4	0	0	-	-	-	51
cOR2A32	23	18	4	4	4	1	0	-	-	-	2
cOR1I2	20	34	2	3	1	1	0	-	-	-	1
cOR2T22	19	18	4	8	0	4	1	-	-	-	2
cOR2A33	28	24	3	2	3	2	0	-	-	-	2
cOR9G7	12	26	6	0	5	0	0	-	-	-	9
cOR4D15	15	18	7	7	1	0	0	-	-	-	4
cOR2AG1	18	18	1	4	1	6	0	-	-	-	2
cOR13D7	24	26	2	5	1	0	1	-	-	-	13
cOR8S10	22	20	4	3	1	4	1	-	-	-	8
cOR13P4	14	21	0	3	2	1	0	-	-	-	13
cOR2AX1	17	18	1	2	1	0	0	-	-	-	2
cOR10J20	13	25	7	0	3	0	0	-	-	-	10
cOR4K22	19	16	4	5	2	0	0	-	-	-	4
cOR2A35	28	23	2	2	1	1	0	-	-	-	2
cOR2L17	11	14	3	3	1	0	0	-	-	-	2
cOR5G3	20	29	7	3	1	0	1	-	-	-	5
cOR4F24	18	9	5	4	7	2	0	-	-	-	4
cOR2F3	22	24	2	1	2	1	0	-	-	-	2
cOR2W14	17	29	5	6	3	0	0	-	-	-	2
cOR10V4	16	17	3	0	3	0	0	-	-	-	10

cOR52U3	14	7	3	3	1	0	0	-	-	-	52
cOR6Q2	19	35	1	1	0	1	0	-	-	-	6
cOR51I1	12	7	11	3	4	0	0	-	-	-	51
cOR4G7	15	10	3	3	3	0	0	-	-	-	4
cOR2Z3	10	16	3	1	1	0	0	-	-	-	2
cOR4X6	23	28	6	6	0	1	0	-	-	-	4
cOR3A10	18	24	1	2	4	2	0	-	-	-	3
cOR11H11	23	25	1	0	2	1	0	-	-	-	11
cOR2A39	20	26	3	1	2	1	0	-	-	-	2
cOR51J3	9	10	11	3	3	0	0	-	-	-	51
cOR51M1	13	1	4	4	1	2	0	-	-	-	51
cOR5W4	20	39	7	1	4	0	0	-	-	-	5
cOR51B8	15	3	8	6	3	0	0	-	-	-	51
cOR4A54	22	18	5	9	2	1	0	-	-	-	4
cOR5L6	26	29	9	3	2	0	0	-	-	-	5
cOR4B4	26	23	4	1	1	0	0	-	-	-	4
cOR51E2	11	5	3	6	2	0	0	-	-	-	51
cOR2C6	16	20	5	3	1	0	0	-	-	-	2
cOR6C33	20	21	0	6	3	1	0	-	-	-	6
cOR2B2	22	35	1	6	4	1	0	-	-	-	2
cOR51A19	10	5	6	1	2	0	0	-	-	-	51
cOR9I2	21	29	5	3	4	0	0	-	-	-	9
cOR52U5	14	7	3	3	1	0	0	-	-	-	52
cOR2AX2	16	14	0	2	2	1	0	-	-	-	2
cOR10A14	23	23	2	2	1	0	0	-	-	-	10
cOR2G5	22	24	6	4	4	0	0	-	-	-	2
cOR51D2	12	5	8	5	1	1	0	-	-	-	51
cOR4C56	26	23	7	8	2	2	0	-	-	-	4
cOR10H9	17	22	0	5	3	1	0	-	-	-	10
cOR5BH3	18	18	4	2	1	0	0	-	-	-	5
cOR10K2	19	20	2	0	3	0	0	-	-	-	10
cOR4C36	25	17	4	10	3	3	1	-	-	-	4
cOR7G13	21	20	2	2	1	2	0	-	-	-	7
cOR5I2	21	30	3	3	3	1	0	-	-	-	5
cOR5D19	19	23	7	3	2	0	0	-	-	-	5
cOR5I1	20	29	3	4	3	1	0	-	-	-	5
cOR10A3	18	21	2	3	4	2	0	-	-	-	10
cOR4D5	16	17	7	2	3	0	0	-	-	-	4
cOR11G3	17	29	5	2	4	2	1	-	-	-	11
cOR10A11	17	26	6	2	3	0	1	-	-	-	10
cOR6D7	12	15	2	1	2	0	0	-	-	-	6
cOR5P4	17	28	5	2	0	1	0	-	-	-	5
cOR8J7	20	23	5	2	3	1	0	-	-	-	8
cOR5BU2	18	13	3	2	2	1	0	-	-	-	5
cOR52D3	16	9	12	5	1	1	0	-	-	-	52
cOR2AZ1	16	14	2	1	1	0	0	-	-	-	2
cOR13D1	25	32	2	3	2	1	0	-	-	-	13
cOR4F25	14	14	8	5	4	0	0	-	-	-	4
cOR5J3	25	38	8	0	1	0	0	-	-	-	5
cOR6M7	18	28	0	0	1	0	0	-	-	-	6
cOR2B9	18	17	2	4	1	0	0	-	-	-	2
cOR4E3	21	30	6	3	1	0	0	-	-	-	4

cOR10A13	17	27	6	2	3	0	1	-	-	-	10
cOR10N1	19	18	4	2	3	0	0	-	-	-	10
cOR5AN2	18	33	5	0	1	1	0	-	-	-	5
cOR2AI2	13	29	1	4	2	0	0	-	-	-	2
cOR2W15	20	19	4	7	1	0	0	-	-	-	2
cOR4P5	26	14	8	5	1	2	0	-	-	-	4
cOR10H20	17	11	0	5	4	3	2	-	-	-	10
cOR4E1	21	25	6	0	1	0	0	-	-	-	4
cOR51K1	12	9	5	5	4	0	0	-	-	-	51
cOR10J23	16	21	4	0	4	0	1	-	-	-	10
cOR4D11	17	15	6	5	0	2	0	-	-	-	4
cOR2T25	18	24	1	6	2	0	0	-	-	-	2
cOR1R4	12	16	1	3	1	0	0	-	-	-	1
cOR13C26	21	30	3	1	3	1	0	-	-	-	13
cOR6A2	21	33	4	0	1	0	0	-	-	-	6
cOR9G8	13	23	5	0	5	0	0	-	-	-	9
cOR4P9	20	16	5	3	2	1	0	-	-	-	4
cOR1AF1	18	18	5	0	0	0	0	-	-	-	1
cOR10AG2	24	28	2	3	0	0	0	-	-	-	10
cOR9K5	20	22	3	1	0	0	0	-	-	-	9
cOR8U6	17	42	8	8	3	0	0	-	-	-	8
cOR7C19	18	32	2	4	4	0	0	-	-	-	7
cOR4C35	26	27	9	12	0	0	0	-	-	-	4
cOR8S13	17	26	1	1	0	5	1	-	-	-	8
cOR11G9	15	28	5	3	4	0	1	-	-	-	11
cOR7D4	20	38	4	5	0	2	0	-	-	-	7
cOR56B6	11	4	1	5	1	0	1	-	-	-	56
cOR5G9	22	29	10	2	0	0	1	-	-	-	5
cOR4G9	13	13	3	4	4	0	0	-	-	-	4
cOR10Q5	18	23	3	2	3	0	0	-	-	-	10
cOR5H13	12	16	5	2	3	2	1	-	-	-	5
cOR5M20	18	27	3	6	2	0	0	-	-	-	5
cOR8U7	18	29	6	6	4	1	0	-	-	-	8
cOR2A36	19	18	3	5	3	0	0	-	-	-	2
cOR8J6	12	22	6	4	2	1	0	-	-	-	8
cOR3A11	17	26	1	2	2	5	0	-	-	-	3
cOR11M3	17	17	2	0	0	0	0	-	-	-	11
cOR4Z3	19	14	7	4	2	0	0	-	-	-	4
cOR10AH1	15	9	4	0	1	0	0	-	-	-	10
cOR8T4	22	25	2	2	1	3	1	-	-	-	8
cOR5M23	20	27	2	2	0	0	0	-	-	-	5
cOR5M13	33	31	1	2	0	0	0	-	-	-	5
cOR5AN4	17	32	11	2	2	0	0	-	-	-	5
cOR5BA2	19	19	4	0	0	0	0	-	-	-	5
cOR5AL1	28	25	3	5	0	0	0	-	-	-	5
cOR51B10	16	2	4	5	3	0	0	-	-	-	51
cOR5AL3	24	27	2	1	1	0	0	-	-	-	5
cOR5M18	15	30	7	6	1	0	0	-	-	-	5
cOR10V6	18	8	2	3	2	1	0	-	-	-	10
cOR2T20	17	21	3	7	1	3	2	-	-	-	2
cOR9G4	19	23	11	1	1	0	0	-	-	-	9
cOR5F3	19	39	6	1	0	0	0	-	-	-	5

cOR1E12	23	31	4	4	7	3	0	-	-	-	1
cOR5AP4	27	41	9	2	1	0	0	-	-	-	5
cOR9K6	22	27	9	1	1	0	0	-	-	-	9
cOR4A39	28	16	5	9	1	2	0	-	-	-	4
cOR10S3	19	21	2	1	4	2	0	-	-	-	10
cOR2Z6	16	17	1	3	0	1	0	-	-	-	2
cOR7E154	26	28	3	5	2	2	0	-	-	-	7
cOR10Q3	22	10	4	1	4	0	0	-	-	-	10
cOR12E4	23	35	3	3	4	0	0	-	-	-	12
cOR8S15	21	25	1	1	1	3	1	-	-	-	8
cOR5T7	13	26	9	1	3	1	0	-	-	-	5
cOR3A12	14	25	0	1	2	5	0	-	-	-	3
cOR56B2	12	4	3	6	1	2	2	-	-	-	56
cOR51B9	15	4	4	6	1	0	0	-	-	-	51
cOR12E8	23	29	6	3	3	0	1	-	-	-	12
cOR6N4	22	19	0	0	3	0	0	-	-	-	6
cOR3A9	18	28	1	2	3	5	0	-	-	-	3
cOR8S17	21	30	2	1	0	3	0	-	-	-	8
cOR2L16	14	18	3	1	1	2	0	-	-	-	2
cOR52B9	14	7	5	5	4	0	0	-	-	-	52
cOR6K5	20	10	1	1	3	0	0	-	-	-	6
cOR6C36	22	20	5	8	3	2	0	-	-	-	6
cOR2T24	19	20	3	8	0	3	2	-	-	-	2
cOR2T15	20	22	3	7	0	3	2	-	-	-	2
cOR13F4	20	26	3	3	4	0	0	-	-	-	13
cOR9A8	12	10	2	1	0	0	0	-	-	-	9
cOR10X2	6	20	2	1	3	0	0	-	-	-	10
cOR1P1	17	22	3	5	3	1	0	-	-	-	1
cOR4S7	20	23	5	4	3	0	0	-	-	-	4
cOR9I5	21	29	4	1	2	0	0	-	-	-	9
cOR2W16	20	25	5	6	0	0	0	-	-	-	2
cOR10AB2	16	16	4	3	1	0	0	-	-	-	10
cOR4G10	16	16	5	6	3	0	0	-	-	-	4
cOR5J1	18	36	8	3	1	0	0	-	-	-	5
cOR4P6	22	16	4	6	1	0	0	-	-	-	4
cOR52K5	18	12	8	4	1	0	0	-	-	-	52
cOR52A6	11	6	8	6	3	0	1	-	-	-	52
cOR4C18	21	12	7	10	4	5	2	-	-	-	4
cOR13D4	25	38	2	3	3	1	0	-	-	-	13
cOR1M2	26	27	3	5	3	0	0	-	-	-	1
cOR8S5	19	26	1	0	0	1	1	-	-	-	8
cOR6V2	10	20	4	0	0	0	0	-	-	-	6
cOR8U2	19	30	6	6	4	0	0	-	-	-	8
cOR9A7	10	14	3	0	0	1	0	-	-	-	9
cOR8H4	23	33	1	0	1	1	0	-	-	-	8
cOR4Y1	20	20	6	2	1	0	1	-	-	-	4
cOR5T5	12	32	10	4	3	1	1	-	-	-	5
cOR52AC1	10	7	3	2	2	0	0	-	-	-	52
cOR10J14	14	21	5	0	4	1	1	-	-	-	10
cOR11S2	12	27	1	0	2	0	0	-	-	-	11
cOR52D4	18	11	13	4	1	1	0	-	-	-	52
cOR11G11	20	29	2	2	4	0	0	-	-	-	11

cOR5J4	21	36	6	1	1	0	0	-	-	-	5
cOR7C51	26	24	4	3	1	1	0	-	-	-	7
cOR6B5	18	23	2	0	0	0	0	-	-	-	6
cOR8S7	20	30	2	3	1	3	0	-	-	-	8
cOR10K3	18	23	2	0	2	0	0	-	-	-	10
cOR9K10	20	27	8	1	1	0	0	-	-	-	9
cOR51R2	14	4	4	2	1	0	0	-	-	-	51
cOR52B7	13	10	7	3	4	0	0	-	-	-	52
cOR10D8	20	17	0	2	2	0	0	-	-	-	10
cOR7D5	19	24	5	3	1	2	0	-	-	-	7
cOR6K7	22	12	1	1	1	0	0	-	-	-	6
cOR4N6	17	17	6	3	1	0	0	-	-	-	4
cOR13L2	17	28	2	1	2	0	0	-	-	-	13
cOR52X2	9	10	8	3	2	0	0	-	-	-	52
cOR5P5	18	27	1	3	4	2	0	-	-	-	5
cOR4D14	16	18	6	2	1	0	0	-	-	-	4
cOR13F5	21	28	2	2	3	1	0	-	-	-	13
cOR1A3	22	18	1	4	2	0	0	-	-	-	1
cOR5AP3	21	31	5	3	3	0	0	-	-	-	5
cOR5H9	13	19	6	3	3	3	1	-	-	-	5
cOR52I2	16	6	1	1	2	0	0	-	-	-	52
cOR13Q4	22	23	2	2	1	0	0	-	-	-	13
cOR10AD2	11	16	0	2	1	0	0	-	-	-	10
cOR5A3	14	30	5	0	2	0	0	-	-	-	5
cOR11S1	16	23	0	0	3	0	0	-	-	-	11
cOR52Z2	19	7	9	3	3	0	1	-	-	-	52
cOR2D4	23	22	2	2	2	1	0	-	-	-	2
cOR1F15	24	31	1	4	1	0	0	-	-	-	1
cOR5BG2	19	29	5	2	1	0	0	-	-	-	5
cOR4S4	23	17	3	3	0	0	0	-	-	-	4
cOR13P2	17	15	1	2	0	0	1	-	-	-	13
cOR2AT6	18	18	1	1	3	1	0	-	-	-	2
cOR5E2	14	34	4	3	2	0	0	-	-	-	5
cOR1L8	26	24	3	1	1	0	0	-	-	-	1
cOR11K4	17	18	5	2	4	0	0	-	-	-	11
cOR8S8	24	19	4	2	0	6	1	-	-	-	8
cOR12E2	16	32	6	1	2	1	1	-	-	-	12
cOR6B6	15	32	4	0	3	1	0	-	-	-	6
cOR2L15	18	16	0	1	3	2	0	-	-	-	2
cOR13R1	26	25	4	2	2	0	0	-	-	-	13
cOR10G11	15	27	0	1	2	0	0	-	-	-	10
cOR4B1	23	22	4	0	1	0	0	-	-	-	4
cOR1D8	10	18	2	4	0	0	0	-	-	-	1
cOR13N4	22	20	1	4	2	2	1	-	-	-	13
cOR11J3	16	27	3	0	4	0	0	-	-	-	11
cOR2AV1	18	14	0	1	1	0	0	-	-	-	2
cOR13M2	21	25	3	1	0	0	0	-	-	-	13
cOR13R2	25	30	4	2	2	0	0	-	-	-	13
cOR13C9	21	27	4	1	3	1	0	-	-	-	13
cOR5BC3	14	33	3	4	4	2	0	-	-	-	5
cOR4A33	15	14	2	4	4	1	1	-	-	-	4
cOR5H12	11	20	7	3	2	2	1	-	-	-	5

cOR52D1	17	14	14	4	1	1	0	-	-	-	52
cOR8S9	16	19	0	1	0	5	1	-	-	-	8
cOR10Q1	18	23	3	2	3	0	0	-	-	-	10
cOR4K20	17	16	6	4	1	1	1	-	-	-	4
cOR5G1	24	27	8	3	1	1	1	-	-	-	5
cOR5T6	13	24	8	1	4	1	1	-	-	-	5
cOR10H15P	18	12	0	4	4	3	2	-	-	-	10
cOR4V2P	20	19	7	3	1	0	3	-	-	-	4
cOR4F30P	13	7	3	3	5	2	0	-	-	-	4
cOR10A15P	13	20	3	3	4	3	0	-	-	-	10
cOR10AC2	11	11	2	0	0	1	0	-	-	-	10
cOR10AB3P	16	16	4	3	1	1	0	-	-	-	10
cOR4P12P	16	10	4	3	1	0	0	-	-	-	4
cOR5L7P	26	29	9	3	2	0	0	-	-	-	5
cOR10V5	19	17	3	2	2	0	0	-	-	-	10
cOR11K3P	13	17	4	0	4	1	0	-	-	-	11
cOR4K18P	18	13	7	1	1	0	0	-	-	-	4
cOR8J4P	18	29	6	4	3	1	0	-	-	-	8
cOR11H10	14	14	1	0	4	1	0	-	-	-	11
cOR2AG8P	18	16	1	4	0	6	0	-	-	-	2
cOR2D2P	23	26	2	4	3	1	0	-	-	-	2
cOR12G1	16	24	4	1	0	0	1	-	-	-	12
cOR4F23P	14	9	6	3	5	1	0	-	-	-	4
cOR4Y2P	20	21	7	4	1	0	3	-	-	-	4
cOR10A10P	17	25	6	2	3	0	1	-	-	-	10
cOR8T2P	19	18	1	1	1	2	2	-	-	-	8
cOR4N5	19	17	6	2	1	1	0	-	-	-	4
cOR2M11	19	13	2	3	1	2	0	-	-	-	2
cOR2A34P	24	11	0	1	2	0	0	-	-	-	2
cOR6Y3	17	25	2	2	1	0	0	-	-	-	6
cOR4K21P	18	21	3	1	4	0	0	-	-	-	4
cOR2A7P	24	20	0	1	1	2	0	-	-	-	2
cOR8S11P	22	19	4	2	0	5	1	-	-	-	8
cOR13P5	20	27	1	2	1	0	0	-	-	-	13
cOR55B3P	12	3	0	2	2	0	0	-	-	-	55
cOR11J4P	14	19	1	1	3	0	1	-	-	-	11
cOR55B4P	12	3	0	1	0	0	0	-	-	-	55
cOR6M4P	18	28	1	0	3	0	0	-	-	-	6
cOR1D10P	20	23	3	4	0	0	0	-	-	-	1
cOR2AV3P	15	11	0	1	1	1	1	-	-	-	2
cOR2F4P	22	21	2	0	2	1	0	-	-	-	2
cOR5M24P	33	31	1	4	0	0	0	-	-	-	5
cOR4F26P	9	11	4	4	3	0	0	-	-	-	4
cOR2L19P	19	18	1	0	0	3	0	-	-	-	2
cOR4Z4P	15	18	6	4	0	2	0	-	-	-	4
cOR6B8	14	26	2	0	3	0	0	-	-	-	6
cOR8I3	15	25	4	3	1	0	0	-	-	-	8
cOR5T9P	12	32	8	4	3	1	1	-	-	-	5
cOR51V9	13	5	8	4	4	1	1	-	-	-	51
cOR4G8P	11	13	4	2	2	0	0	-	-	-	4
cOR1J6P	18	20	1	3	4	0	0	-	-	-	1
cOR52L3P	12	7	8	4	2	0	0	-	-	-	52

cOR8S21P	19	19	2	0	0	1	2	-	-	-	8
cOR10J7P	15	19	5	0	2	0	0	-	-	-	10
cOR4F22P	13	7	3	4	3	2	0	-	-	-	4
cOR13M1P	22	24	2	1	2	1	0	-	-	-	13
cOR1F14P	21	32	3	1	0	0	0	-	-	-	1
cOR5K6P	17	15	1	1	4	0	0	-	-	-	5
cOR51I3P	17	1	4	1	4	0	0	-	-	-	51
cOR9G1P	13	22	5	0	4	0	0	-	-	-	9
cOR2AK3	18	16	3	0	1	0	0	-	-	-	2
cOR10AD3	11	17	0	4	1	0	0	-	-	-	10
cOR2Z2	19	19	2	1	2	0	0	-	-	-	2
cOR4L4	16	18	3	2	3	0	0	-	-	-	4
cOR4C26P	18	24	4	8	0	0	0	-	-	-	4
cOR10AD1	12	17	0	4	1	0	0	-	-	-	10
cOR13L1P	6	22	1	1	2	0	0	-	-	-	13
cOR5AK6P	19	27	6	1	3	1	0	-	-	-	5
cOR2D10P	19	18	1	4	3	1	0	-	-	-	2
cOR11G12	13	33	2	0	2	1	0	-	-	-	11
cOR52E22	16	12	5	3	2	0	0	-	-	-	52
cOR52AD1	15	5	1	2	1	0	1	-	-	-	52
cOR5A6	11	32	4	0	0	0	0	-	-	-	5
cOR52J8	12	8	11	4	2	0	0	-	-	-	52
cOR51T2	15	6	6	1	2	0	0	-	-	-	51
cOR4S8	28	19	4	3	0	0	0	-	-	-	4
cOR4Y3	15	15	7	3	1	0	3	-	-	-	4
cOR51G2	14	9	8	3	4	0	0	-	-	-	51
cOR52R3	11	11	5	3	1	0	0	-	-	-	52
cOR4Q4	21	26	7	2	0	0	1	-	-	-	4
cOR4H14	19	16	4	2	1	1	0	-	-	-	4
cOR4K6	18	22	3	1	1	0	0	-	-	-	4
cOR51A18	9	5	5	3	4	0	0	-	-	-	51
cOR4K28	17	16	3	2	2	0	0	-	-	-	4
cOR2V4	19	16	3	4	1	1	0	-	-	-	2
cOR4M3	16	17	6	1	2	0	0	-	-	-	4
cOR51A16	10	9	4	2	4	0	0	-	-	-	51
cOR6AE1	15	23	1	0	1	0	0	-	-	-	6
cOR51V7P	13	5	7	4	4	1	1	-	-	-	51
cOR2A37	22	16	4	2	2	0	0	-	-	-	2
cOR12J1	20	21	0	0	1	0	1	-	-	-	12
cOR4Q9P	23	24	6	2	0	0	1	-	-	-	4
cOR5AN3P	14	30	10	2	3	0	0	-	-	-	5
cOR6F2	19	11	0	0	0	0	0	-	-	-	6
cOR6B7	11	16	2	0	3	0	0	-	-	-	6
cOR10R5	11	22	4	1	3	0	0	-	-	-	10
cOR51A17	8	9	4	3	4	0	0	-	-	-	51
cOR13Q3	16	17	2	0	1	0	0	-	-	-	13
cOR5E1P	12	29	4	3	1	0	0	-	-	-	5
cOR51AA1	12	6	5	4	4	0	0	-	-	-	51
