# Supplementary material for: Common peptides shed light on evolution of Olfactory Receptors
Source: BMC Evol Biol. 2009 May 5;9:91. doi: 10.1186/1471-2148-9-91 (PMC2681464; doi:10.1186/1471-2148-9-91)
Supplement: Additional file 8 — Mouse ORs CP numbers and cluster assignment. Number of CPs from each ancestor occurring in each Mouse OR and cluster assignment for each Mouse OR. [file 1471-2148-9-91-S8.pdf]

Legend

A	Number of A1 CPs
B	Number of A2 novel CPs
C	Number of A3 novel CPs
D	Number of A4 novel CPs
E	Number of A5 novel CPs
F	Number of A6 novel CPs
G	Number of mouse novel CPs
H	Cluster number A4 novel CPs
I	Cluster number A5 novel CPs
J	Cluster number mouse novel CPs
K	Family

Name	A	B	C	D	E	F	G	H	I	J	K
MOR245-1	17	11	7	5	5	0	0	1	10	-	4
MOR245-22	16	10	5	5	5	0	0	1	10	-	4
MOR245-18	9	13	5	6	3	0	0	1	-	-	4
MOR245-20	17	10	6	5	4	0	1	1	-	-	4
MOR245-11	10	12	8	6	3	0	0	1	-	-	4
MOR240-3	16	18	7	6	1	0	0	1	-	-	4
MOR240-1	12	15	9	6	3	1	0	1	-	-	4
MOR239-7	20	17	5	5	0	4	0	1	-	-	4
MOR239-2	19	16	5	5	1	4	0	1	-	-	4
MOR239-1	21	17	7	6	0	4	0	1	-	-	4
MOR239-4	15	20	5	5	1	2	0	1	-	-	4
MOR112-2	22	22	2	5	6	0	0	2	8	-	6
MOR114-14	24	23	2	10	16	0	0	2	8	-	6
MOR111-13	23	26	3	8	6	0	1	2	8	-	6
MOR111-4	22	21	3	8	5	0	0	2	8	-	6
MOR111-10	22	21	2	9	11	0	0	2	8	-	6
MOR114-11	21	19	2	8	12	1	1	2	8	-	6
MOR114-12	20	18	5	7	13	1	0	2	8	-	6
MOR110-9	19	18	2	9	7	1	2	2	8	-	6
MOR110-6	21	22	2	12	8	1	1	2	8	-	6
MOR108-1	25	22	3	7	5	0	0	2	8	-	6
MOR117-1	23	13	1	5	5	0	0	2	8	-	6
MOR109-1	21	26	3	8	8	1	0	2	8	-	6
MOR111-12	20	20	2	10	8	1	0	2	8	-	6
MOR114-7	20	16	3	6	9	0	0	2	8	-	6
MOR114-8	22	15	2	5	8	1	0	2	8	-	6
MOR111-3	17	27	5	7	8	1	0	2	8	-	6
MOR110-1	23	19	2	12	9	1	2	2	8	-	6
MOR110-2	20	19	3	9	7	1	2	2	8	-	6
MOR110-7	21	15	4	5	9	1	1	2	8	-	6
MOR110-10	21	20	1	11	5	0	1	2	8	-	6
MOR114-3	16	13	5	5	9	0	0	2	8	-	6
MOR113-3	19	19	1	7	5	1	1	2	8	-	6
MOR114-1	21	19	5	7	15	1	0	2	8	-	6
MOR114-9	20	16	3	6	8	0	0	2	8	-	6
MOR111-2	19	23	2	6	10	1	1	2	8	-	6
MOR111-5	25	20	5	9	6	0	0	2	8	-	6
MOR114-5	22	14	3	6	9	2	0	2	8	-	6
MOR113-8	19	20	1	7	6	1	1	2	8	-	6
MOR110-4	21	21	2	12	2	1	2	2	-	-	6
MOR112-1	26	21	3	12	4	1	0	2	-	-	6
MOR115-1	21	23	1	11	4	0	0	2	-	-	6

MOR116-1	23	18	0	9	3	0	0	2	-	-	6
MOR108-4	22	19	4	9	4	1	0	2	-	-	6
MOR110-3	19	20	3	13	4	1	2	2	-	-	6
MOR111-1	25	22	6	8	2	0	0	2	-	-	6
MOR115-4	23	23	1	11	4	0	0	2	-	-	6
MOR113-4	18	21	2	6	3	1	0	2	-	-	6
MOR108-3	22	21	4	9	2	1	0	2	-	-	6
MOR171-8	18	33	7	5	6	0	1	3	12	-	8
MOR171-47	17	31	7	5	7	1	1	3	12	-	8
MOR200-1	23	25	5	5	3	0	0	3	-	-	5
MOR277-1	19	21	1	6	2	1	0	3	-	-	2
MOR199-1	16	27	3	5	3	1	0	3	-	-	5
MOR199-2	15	25	3	5	3	1	0	3	-	-	5
MOR278-1	18	15	2	6	0	2	0	3	-	-	2
MOR278-2	21	17	3	9	0	0	0	3	-	-	2
MOR32-12	14	6	15	5	5	0	0	4	1	-	52
MOR31-4	11	10	5	5	5	0	0	4	1	-	52
MOR31-12	12	11	8	6	8	0	0	4	1	-	52
MOR31-8	17	6	9	7	7	0	0	4	1	-	52
MOR32-11	18	12	11	6	5	0	0	4	1	-	52
MOR32-10	20	11	13	7	9	0	0	4	1	-	52
MOR32-5	22	7	14	5	5	0	0	4	1	-	52
MOR34-12	6	9	6	6	5	0	0	4	3	-	52
MOR34-3	11	4	9	6	5	1	0	4	3	-	52
MOR34-5	9	9	9	8	6	0	0	4	3	-	52
MOR13-3	15	9	5	8	5	1	0	4	5	-	51
MOR32-4	21	8	16	5	5	0	0	4	5	-	52
MOR5-1	10	10	5	5	6	0	0	4	5	-	51
MOR7-1	13	10	10	5	5	0	0	4	5	-	51
MOR17-2	14	6	10	6	5	0	0	4	5	-	51
MOR8-5	10	10	6	5	7	0	0	4	5	-	51
MOR6-1	11	6	7	8	7	0	0	4	5	-	51
MOR5-2	9	9	5	5	6	0	1	4	5	-	51
MOR10-1	14	7	9	7	5	0	0	4	5	-	51
MOR17-1	14	7	9	9	6	0	0	4	5	-	51
MOR10-2	9	8	13	6	5	0	0	4	6	-	51
MOR24-4	11	8	8	5	1	0	0	4	-	-	52
MOR18-2	11	6	4	7	1	0	0	4	-	-	51
MOR31-2	15	9	10	7	3	0	0	4	-	-	52
MOR18-1	19	8	4	6	2	0	0	4	-	-	51
MOR31-6	12	10	9	8	4	0	0	4	-	-	52
MOR33-1	18	10	10	5	1	2	0	4	-	-	52
MOR27-1	19	8	10	5	0	0	0	4	-	-	52
MOR22-1	14	9	10	8	4	0	0	4	-	-	52
MOR30-2	11	9	11	6	1	0	0	4	-	-	52
MOR23-2	13	8	11	5	2	0	0	4	-	-	52
MOR22-2	13	7	6	8	2	0	0	4	-	-	52
MOR1-3	10	4	6	5	2	0	0	4	-	-	51
MOR1-1	10	4	8	6	0	0	0	4	-	-	51
MOR34-4	10	9	6	5	4	1	0	4	-	-	52
MOR32-13	9	12	11	7	2	0	0	4	-	-	52
MOR23-5	12	8	10	5	3	0	0	4	-	-	52
MOR26-3	10	8	8	7	2	1	0	4	-	-	52
MOR24-3	10	4	10	7	4	0	0	4	-	-	52
MOR31-5	16	7	9	6	4	0	1	4	-	-	52
MOR23-6	12	8	10	5	3	0	0	4	-	-	52

MOR37-2	14	7	8	6	1	0	0	4	-	-	52
MOR24-2	13	9	8	8	1	0	0	4	-	-	52
MOR22-4	13	9	9	8	4	0	0	4	-	-	52
MOR31-13	11	9	4	6	1	0	0	4	-	-	52
MOR13-5	14	6	4	8	4	0	0	4	-	-	51
MOR32-8	22	7	18	5	4	0	1	4	-	-	52
MOR9-2	7	8	8	5	4	1	0	4	-	-	51
MOR32-1	16	10	14	6	3	0	0	4	-	-	52
MOR3-1	13	3	4	5	2	2	0	4	-	-	51
MOR13-6	16	4	4	6	4	0	0	4	-	-	51
MOR26-1	12	9	7	6	2	1	0	4	-	-	52
MOR24-5	11	5	10	8	3	0	0	4	-	-	52
MOR34-11	8	3	5	5	4	1	0	4	-	-	52
MOR256-64	18	26	8	5	0	0	0	5	-	-	2
MOR256-15	14	32	3	5	3	0	0	5	-	-	2
MOR256-8	19	18	4	7	0	0	0	5	-	-	2
MOR256-12	21	27	5	6	0	0	0	5	-	-	2
MOR256-13	18	26	4	5	0	0	0	5	-	-	2
MOR256-47	21	26	8	5	0	0	0	5	-	-	2
MOR256-51	17	21	3	6	0	0	0	5	-	-	2
MOR256-61	15	27	2	8	1	0	1	5	-	-	2
MOR256-33	12	28	4	6	0	0	1	5	-	-	2
MOR256-19	11	28	4	6	0	0	1	5	-	-	2
MOR256-9	15	23	5	5	0	0	1	5	-	-	2
MOR256-31	15	24	3	8	1	0	1	5	-	-	2
MOR256-11	20	27	2	5	3	0	0	5	-	-	2
MOR204-8	21	33	2	6	6	3	5	6	2	2	5
MOR204-7	18	21	3	5	5	3	6	6	2	2	5
MOR204-16	22	31	4	5	4	2	5	6	-	2	5
MOR204-35	15	31	2	7	3	3	5	6	-	2	5
MOR204-10	22	27	3	5	3	2	7	6	-	2	5
MOR204-12	16	27	3	5	3	1	5	6	-	2	5
MOR204-9	19	27	2	6	3	1	6	6	-	2	5
MOR204-13	17	27	3	7	3	2	3	6	-	-	5
MOR204-38	14	27	2	5	0	1	2	6	-	-	5
MOR204-11	23	30	3	6	2	1	0	6	-	-	5
MOR204-33	18	29	2	7	0	1	2	6	-	-	5
MOR204-6	16	34	3	7	2	0	0	6	-	-	5
MOR204-29	18	25	3	6	3	1	3	6	-	-	5
MOR204-19	13	25	4	6	2	1	2	6	-	-	5
MOR204-32	17	32	4	5	2	0	0	6	-	-	5
MOR40-1	16	2	3	9	5	0	1	7	-	-	56
MOR40-15	13	8	1	6	2	0	0	7	-	-	56
MOR40-13	13	7	1	6	2	1	0	7	-	-	56
MOR38-2	13	4	3	5	1	0	0	7	-	-	52
MOR40-17	16	2	3	9	4	0	1	7	-	-	56
MOR40-14	13	2	4	9	6	0	0	7	-	-	56
MOR40-2	13	3	4	9	6	0	0	7	-	-	56
MOR40-4	13	4	2	5	1	0	0	7	-	-	56
MOR185-4	19	30	4	6	6	0	0	8	2	-	8
MOR185-6	18	33	5	6	5	1	0	8	2	-	8
MOR185-11	13	29	6	5	5	0	1	8	12	-	8
MOR196-2	17	34	5	6	1	1	0	8	-	-	5
MOR185-3	11	34	6	7	3	1	0	8	-	-	8
MOR185-2	18	32	7	5	3	0	0	8	-	-	8
MOR274-1	16	23	1	6	6	0	0	8	-	-	2

MOR185-12	19	22	6	6	1	0	1	8	-	-	8
MOR171-52	25	34	6	9	2	0	0	8	-	-	8
MOR185-5	22	23	5	6	1	0	1	8	-	-	8
MOR256-59	17	22	0	5	3	0	1	8	-	-	2
MOR187-4	16	28	4	7	1	0	1	8	-	-	8
MOR256-3	13	29	3	6	3	0	0	8	-	-	2
MOR275-1	18	21	1	7	3	1	0	8	-	-	2
MOR276-1	19	19	2	6	1	2	0	8	-	-	2
MOR256-60	22	25	2	7	4	1	0	8	-	-	2
MOR256-35	22	27	2	7	3	1	0	8	-	-	2
MOR256-7	18	22	0	5	3	0	1	8	-	-	2
MOR256-55	13	27	5	6	6	0	0	9	1	-	2
MOR256-25	9	27	6	6	6	0	0	9	1	-	2
MOR268-2	17	25	1	5	2	2	3	9	-	-	10
MOR264-17	24	21	3	6	1	0	1	9	-	-	10
MOR256-2	14	20	2	7	2	0	0	9	-	-	2
MOR264-22	20	29	1	5	0	0	1	9	-	-	10
MOR256-30	21	12	4	5	0	0	0	9	-	-	2
MOR265-1	17	22	0	5	3	0	0	9	-	-	10
MOR256-56	11	25	2	5	3	0	2	9	-	-	2
MOR256-41	9	33	7	6	5	0	0	9	-	-	2
MOR256-27	13	24	5	5	4	0	1	9	-	-	2
MOR188-3	17	22	5	5	5	0	0	10	4	-	8
MOR171-16	22	35	10	6	11	0	1	10	12	-	8
MOR171-7	20	29	7	5	13	0	1	10	12	-	8
MOR171-13	21	36	8	5	10	1	0	10	12	-	8
MOR171-17	23	32	6	5	6	0	0	10	12	-	8
MOR171-14	23	33	9	6	11	2	0	10	12	-	8
MOR171-48	22	30	9	5	16	0	0	10	12	-	8
MOR189-1	18	28	5	5	5	0	0	10	12	-	8
MOR171-10	19	27	7	5	3	0	1	10	-	-	8
MOR171-11	23	26	7	5	3	0	1	10	-	-	8
MOR188-8	15	24	2	8	2	0	0	10	-	-	8
MOR189-3	18	28	3	7	4	0	0	10	-	-	8
MOR193-1	17	16	3	5	2	0	0	10	-	-	8
MOR188-2	20	24	1	6	4	1	0	10	-	-	8
MOR187-1	19	23	9	5	3	0	1	10	-	-	8
MOR188-9	14	25	1	6	2	0	0	10	-	-	8
MOR189-2	15	29	5	6	4	0	1	10	-	-	8
MOR191-1	14	27	5	5	2	0	0	10	-	-	8
MOR187-5	19	23	9	5	3	0	1	10	-	-	8
MOR228-3	23	24	5	5	0	0	0	11	-	-	4
MOR230-2	25	14	6	7	4	3	0	11	-	-	4
MOR225-6	27	20	6	5	0	1	1	11	-	-	4
MOR232-1	22	22	7	10	2	0	0	11	-	-	4
MOR238-1	19	16	3	5	1	0	0	11	-	-	4
MOR232-7	22	26	6	11	3	0	1	11	-	-	4
MOR232-6	24	25	6	11	2	0	1	11	-	-	4
MOR230-1	24	13	6	6	3	2	0	11	-	-	4
MOR231-13	23	22	5	9	0	4	1	11	-	-	4
MOR232-3	27	23	8	11	0	0	0	11	-	-	4
MOR225-13	13	14	3	6	1	1	0	11	-	-	4
MOR230-6	25	15	7	11	2	1	0	11	-	-	4
MOR231-2	22	18	5	10	3	2	0	11	-	-	4
MOR234-3	24	17	5	9	1	0	0	11	-	-	4
MOR230-3	24	13	10	10	3	4	0	11	-	-	4

MOR230-5	21	15	13	10	1	2	0	11	-	-	4
MOR174-10	16	28	3	5	4	0	0	11	-	-	5
MOR233-18	23	17	7	5	0	0	1	11	-	-	4
MOR234-1	24	19	4	9	2	0	0	11	-	-	4
MOR235-2	27	16	6	8	1	0	0	11	-	-	4
MOR231-3	27	24	8	8	2	2	0	11	-	-	4
MOR232-5	23	25	5	8	1	0	0	11	-	-	4
MOR232-2	27	26	5	10	0	0	0	11	-	-	4
MOR233-11	20	24	7	6	3	2	2	11	-	-	4
MOR233-22	20	18	8	8	0	2	2	11	-	-	4
MOR231-12	27	17	7	8	1	1	0	11	-	-	4
MOR231-10	25	15	5	8	0	3	1	11	-	-	4
MOR228-2	25	26	5	5	0	0	0	11	-	-	4
MOR232-4	24	21	7	8	2	1	0	11	-	-	4
MOR225-2	22	5	4	7	1	3	0	11	-	-	4
MOR231-8	26	13	5	9	1	4	1	11	-	-	4
MOR230-7	28	20	9	10	1	2	0	11	-	-	4
MOR233-9	19	22	4	5	2	1	1	11	-	-	4
MOR233-12	18	17	6	6	1	1	2	11	-	-	4
MOR231-9	24	14	5	6	1	2	2	11	-	-	4
MOR237-2	19	17	5	8	0	0	0	11	-	-	4
MOR235-1	21	15	7	6	1	1	0	11	-	-	4
MOR231-6	24	15	4	10	0	5	0	11	-	-	4
MOR231-1	25	17	4	6	2	2	0	11	-	-	4
MOR231-18	23	16	6	6	1	1	2	11	-	-	4
MOR231-14	27	19	3	8	0	2	0	11	-	-	4
MOR230-8	25	15	9	9	1	2	0	11	-	-	4
MOR232-9	24	22	7	7	1	1	0	11	-	-	4
MOR225-4	19	13	3	5	1	2	0	11	-	-	4
MOR231-11	29	21	7	8	1	3	2	11	-	-	4
MOR233-7	24	26	11	6	1	0	0	11	-	-	4
MOR231-4	22	13	5	5	0	2	1	11	-	-	4
MOR233-4	22	24	7	6	2	3	1	11	-	-	4
MOR237-1	21	21	4	8	0	1	1	11	-	-	4
MOR230-11	21	15	7	10	2	1	0	11	-	-	4
MOR264-25	23	34	5	6	5	0	2	12	9	-	13
MOR136-7	25	28	1	5	6	2	5	12	11	3	1
MOR136-13	26	29	0	7	7	0	0	12	11	-	1
MOR135-29	19	31	7	6	8	4	2	12	11	-	1
MOR135-11	23	34	6	6	8	3	2	12	11	-	1
MOR135-12	19	31	7	6	8	4	2	12	11	-	1
MOR139-4	13	27	4	6	6	2	0	12	12	-	7
MOR144-1	18	32	2	7	2	1	0	12	-	-	7
MOR131-1	25	36	2	6	1	0	1	12	-	-	1
MOR138-1	18	31	3	5	3	0	0	12	-	-	1
MOR138-7	18	30	4	5	2	0	0	12	-	-	1
MOR141-1	23	35	1	7	2	3	1	12	-	-	7
MOR153-1	17	20	3	5	1	2	1	12	-	-	7
MOR139-6	20	26	5	7	2	2	0	12	-	-	7
MOR139-1	21	38	4	8	6	2	0	12	-	-	7
MOR132-1	25	27	3	5	1	0	0	12	-	-	1
MOR152-3	13	22	1	7	3	3	0	12	-	-	7
MOR128-4	19	32	3	6	1	3	0	12	-	-	1
MOR139-3	17	35	4	5	4	2	0	12	-	-	7
MOR140-1	23	24	5	8	5	4	1	12	-	-	7
MOR134-1	22	18	1	7	1	0	0	12	-	-	1

MOR139-2	15	28	4	6	5	3	0	12	-	-	7
MOR130-1	25	31	2	6	3	0	0	12	-	-	1
MOR149-2	18	15	3	5	0	1	1	12	-	-	7
MOR32-3	13	13	9	4	6	1	0	-	1	-	52
MOR32-9	14	6	15	4	5	0	0	-	1	-	52
MOR31-7	15	12	7	4	8	1	0	-	1	-	52
MOR31-11	18	8	8	4	5	1	0	-	1	-	52
MOR262-14	26	36	0	4	6	3	0	-	1	-	13
MOR262-5	26	36	0	4	6	2	0	-	1	-	13
MOR202-16	11	27	5	2	8	2	1	-	2	-	5
MOR202-37	18	33	6	4	8	3	3	-	2	-	5
MOR202-44	15	31	7	1	8	3	1	-	2	-	5
MOR202-18	14	35	7	4	8	2	2	-	2	-	5
MOR202-2	11	28	5	1	5	1	0	-	2	-	5
MOR202-33	16	31	4	2	6	0	0	-	2	-	5
MOR202-11	13	31	8	1	9	1	2	-	2	-	5
MOR202-10	16	28	7	4	8	3	2	-	2	-	5
MOR202-39	16	29	5	2	7	2	1	-	2	-	5
MOR202-34	15	30	3	2	7	0	0	-	2	-	5
MOR202-14	10	30	4	2	9	3	3	-	2	-	5
MOR202-8	17	23	4	2	7	1	0	-	2	-	5
MOR202-46	11	27	5	2	8	2	1	-	2	-	5
MOR202-17	10	32	4	1	9	2	2	-	2	-	5
MOR204-23	19	26	2	4	5	1	4	-	2	-	5
MOR202-1	20	20	5	2	7	0	1	-	2	-	5
MOR202-40	20	20	5	2	7	0	1	-	2	-	5
MOR202-35	19	33	7	1	6	0	1	-	2	-	5
MOR202-20	12	25	5	0	5	1	1	-	2	-	5
MOR213-5	17	22	6	1	6	0	0	-	2	-	9
MOR202-42	14	28	6	3	9	2	3	-	2	-	5
MOR202-45	18	33	6	4	8	3	3	-	2	-	5
MOR202-3	9	23	4	2	6	0	0	-	2	-	5
MOR202-13	15	23	3	1	7	3	2	-	2	-	5
MOR202-4	18	42	3	2	9	0	0	-	2	-	5
MOR204-17	19	26	3	4	5	3	3	-	2	-	5
MOR202-28	13	26	3	1	6	1	0	-	2	-	5
MOR213-2	15	19	9	1	5	0	0	-	2	-	9
MOR202-12	16	27	8	1	9	2	1	-	2	-	5
MOR34-7	11	6	6	4	5	0	1	-	3	-	52
MOR34-6	12	10	9	4	5	1	0	-	3	-	52
MOR176-1	15	43	5	3	6	0	0	-	4	-	5
MOR223-1	15	22	1	4	6	4	0	-	4	-	10
MOR223-3	17	22	1	4	6	5	0	-	4	-	10
MOR224-6	24	12	1	2	8	0	0	-	4	-	10
MOR224-3	25	12	2	2	7	1	0	-	4	-	10
MOR224-13	17	12	0	2	5	0	0	-	4	-	10
MOR224-8	24	12	1	2	6	0	0	-	4	-	10
MOR224-2	22	19	0	3	5	0	0	-	4	-	10
MOR177-8	16	28	8	3	5	2	1	-	4	-	8
MOR186-2	15	23	7	4	5	0	0	-	4	-	8
MOR190-1	20	29	5	3	5	0	0	-	4	-	8
MOR177-20	13	22	6	2	5	2	1	-	4	-	8
MOR208-1	18	28	6	3	8	1	1	-	4	-	9
MOR224-5	15	10	0	2	5	0	0	-	4	-	10
MOR176-2	15	46	5	3	6	0	0	-	4	-	5
MOR177-7	17	28	4	4	5	3	1	-	4	-	8

MOR208-2	15	21	6	2	7	1	1	-	4	-	9
MOR208-5	17	28	6	1	6	0	1	-	4	-	9
MOR177-3	12	19	4	2	5	2	1	-	4	-	8
MOR177-14	14	30	8	1	5	2	1	-	4	-	8
MOR177-12	11	22	9	3	5	1	0	-	4	-	8
MOR14-2	15	11	5	3	6	0	0	-	5	-	51
MOR14-7	20	6	6	3	6	0	1	-	5	-	51
MOR14-1	14	12	6	4	10	0	1	-	5	-	51
MOR14-6	15	7	7	3	6	0	1	-	5	-	51
MOR14-4	18	7	6	3	5	0	1	-	5	-	51
MOR23-1	17	9	15	2	6	0	0	-	5	-	52
MOR8-1	10	11	7	3	6	0	0	-	5	-	51
MOR7-2	13	10	5	3	5	0	0	-	5	-	51
MOR4-1	17	3	7	4	5	0	0	-	5	-	51
MOR9-1	9	8	9	4	6	0	0	-	5	-	51
MOR8-3	11	9	8	4	5	0	0	-	5	-	51
MOR2-1	11	1	3	3	7	0	0	-	5	-	51
MOR182-5	15	17	6	2	7	5	1	-	6	-	5
MOR183-9	15	21	9	1	5	5	1	-	6	-	5
MOR183-5	16	21	5	1	5	1	0	-	6	-	5
MOR182-8	14	21	6	2	5	3	2	-	6	-	5
MOR182-13	15	17	6	2	7	5	1	-	6	-	5
MOR182-14	12	21	4	1	6	2	1	-	6	-	5
MOR267-13	21	18	4	1	5	2	1	-	6	-	10
MOR184-3	16	16	2	0	7	0	0	-	6	-	5
MOR183-2	14	20	7	0	6	0	0	-	6	-	5
MOR184-1	18	16	2	1	6	0	0	-	6	-	5
MOR182-12	13	21	6	2	7	5	1	-	6	-	5
MOR182-1	18	17	7	2	6	2	1	-	6	-	5
MOR183-8	17	22	3	1	7	2	0	-	6	-	5
MOR182-10	8	21	6	2	5	3	2	-	6	-	5
MOR106-13	17	22	5	2	5	2	5	-	6	-	11
MOR184-7	19	16	1	1	6	0	0	-	6	-	5
MOR184-5	18	14	3	1	5	0	0	-	6	-	5
MOR183-4	15	19	5	1	6	0	0	-	6	-	5
MOR106-11	18	24	3	0	6	0	0	-	6	-	11
MOR253-7	21	18	1	3	10	1	5	-	7	1	13
MOR253-6	20	18	1	3	11	1	5	-	7	1	13
MOR253-10	19	14	2	2	9	2	5	-	7	1	13
MOR253-8	22	17	1	3	9	0	7	-	7	1	13
MOR253-3	21	15	1	3	10	2	7	-	7	1	13
MOR253-1	20	16	1	3	10	2	5	-	7	1	13
MOR4-2	14	5	7	4	5	0	0	-	7	-	51
MOR253-2	19	14	0	3	8	2	2	-	7	-	13
MOR253-5	20	16	2	3	11	2	3	-	7	-	13
MOR262-8	25	30	1	3	5	1	0	-	7	-	13
MOR253-9	23	20	0	3	5	1	0	-	7	-	13
MOR253-4	20	15	1	3	10	1	3	-	7	-	13
MOR252-2	17	9	2	1	5	0	0	-	7	-	13
MOR113-2	19	24	1	4	5	3	0	-	8	-	6
MOR114-6	16	17	3	3	9	1	0	-	8	-	6
MOR114-2	17	17	3	3	9	1	0	-	8	-	6
MOR114-13	13	12	5	4	8	0	0	-	8	-	6
MOR103-6	14	13	1	0	6	0	0	-	9	-	6
MOR103-8	15	20	7	1	6	0	0	-	9	-	6
MOR103-7	13	23	5	0	6	0	0	-	9	-	6

MOR103-9	13	19	6	0	6	0	0	-	9	-	6
MOR247-4	16	19	7	4	5	0	0	-	10	-	4
MOR245-6	17	11	7	2	5	1	0	-	10	-	4
MOR248-7	15	15	6	2	5	1	1	-	10	-	4
MOR245-5	15	9	4	4	5	0	0	-	10	-	4
MOR245-21	18	13	6	4	6	0	0	-	10	-	4
MOR245-3	19	14	7	4	5	0	0	-	10	-	4
MOR247-2	16	19	7	4	5	0	0	-	10	-	4
MOR248-4	23	16	6	0	5	1	1	-	10	-	4
MOR274-2	16	17	0	3	6	0	0	-	10	-	2
MOR248-10	18	18	5	1	5	1	1	-	10	-	4
MOR248-3	19	14	7	4	5	1	1	-	10	-	4
MOR245-4	23	16	4	4	7	0	0	-	10	-	4
MOR245-12	13	13	2	2	5	0	0	-	10	-	4
MOR136-2	25	20	1	0	7	1	6	-	11	3	1
MOR136-4	26	24	1	3	7	1	6	-	11	3	1
MOR136-3	25	25	1	4	6	0	5	-	11	3	1
MOR136-11	24	27	1	2	5	0	5	-	11	3	1
MOR135-13	21	28	3	2	5	3	2	-	11	-	1
MOR13-2	15	6	1	4	6	1	0	-	11	-	51
MOR136-6	26	29	1	0	7	1	3	-	11	-	1
MOR136-12	22	26	2	4	5	0	4	-	11	-	1
MOR136-1	27	24	1	2	6	1	4	-	11	-	1
MOR136-10	26	27	0	1	8	1	1	-	11	-	1
MOR136-9	25	23	1	1	8	1	4	-	11	-	1
MOR136-8	25	24	0	2	8	0	0	-	11	-	1
MOR136-5	26	21	0	1	8	0	4	-	11	-	1
MOR171-46	14	25	6	3	5	0	0	-	12	-	8
MOR171-24	19	30	12	4	11	2	0	-	12	-	8
MOR167-1	19	25	4	1	11	1	1	-	12	-	8
MOR171-23	20	31	7	4	11	1	0	-	12	-	8
MOR171-19	16	19	5	1	7	1	2	-	12	-	8
MOR162-13	13	29	1	0	6	2	2	-	12	-	8
MOR162-4	14	29	3	1	11	3	1	-	12	-	8
MOR107-1	16	28	0	1	6	2	0	-	12	-	11
MOR171-4	17	32	5	3	6	0	0	-	12	-	8
MOR171-1	18	31	6	3	7	2	0	-	12	-	8
MOR171-12	23	23	6	2	9	0	0	-	12	-	8
MOR164-3	16	28	5	1	9	1	0	-	12	-	8
MOR164-2	18	26	3	1	10	1	0	-	12	-	8
MOR171-28	17	33	5	3	7	2	0	-	12	-	8
MOR167-2	16	34	3	1	16	0	0	-	12	-	8
MOR164-1	18	28	5	0	12	0	0	-	12	-	8
MOR171-22	15	25	6	2	5	0	0	-	12	-	8
MOR165-5	16	24	2	1	6	1	2	-	12	-	8
MOR170-5	15	26	1	3	5	1	1	-	12	-	8
MOR170-6	21	22	2	3	6	1	0	-	12	-	8
MOR165-4	20	34	6	2	9	0	2	-	12	-	8
MOR170-4	18	22	0	2	8	1	1	-	12	-	8
MOR171-20	19	29	6	3	7	2	3	-	12	-	8
MOR168-1	12	32	2	3	6	0	0	-	12	-	8
MOR171-50	15	21	7	2	7	2	1	-	12	-	8
MOR171-25	17	20	7	3	9	1	1	-	12	-	8
MOR171-21	12	20	7	1	6	2	1	-	12	-	8
MOR171-41	16	25	6	2	6	1	3	-	12	-	8
MOR171-5	19	27	11	4	13	2	3	-	12	-	8

MOR171-2	21	36	4	2	6	0	0	-	12	-	8
MOR171-44	19	25	7	2	7	1	2	-	12	-	8
MOR165-10	16	29	4	1	10	0	2	-	12	-	8
MOR161-3	15	34	2	1	7	1	0	-	12	-	8
MOR165-8	20	29	5	1	8	1	1	-	12	-	8
MOR165-1	16	34	3	3	10	1	3	-	12	-	8
MOR167-3	17	35	3	1	16	0	0	-	12	-	8
MOR171-9	15	22	6	3	5	0	0	-	12	-	8
MOR161-4	22	28	7	3	9	1	1	-	12	-	8
MOR161-1	21	26	9	4	12	1	0	-	12	-	8
MOR162-7	14	27	3	2	5	2	3	-	12	-	8
MOR165-7	20	32	3	1	10	1	2	-	12	-	8
MOR161-6	22	30	9	1	16	1	1	-	12	-	8
MOR170-13	19	20	2	2	6	1	0	-	12	-	8
MOR171-18	20	28	7	4	10	1	0	-	12	-	8
MOR171-51	19	27	7	3	7	1	2	-	12	-	8
MOR179-3	14	26	6	2	5	0	1	-	12	-	5
MOR168-2	12	28	3	1	5	0	0	-	12	-	8
MOR162-6	14	24	3	1	11	3	4	-	12	-	8
MOR163-1	19	33	4	1	9	1	0	-	12	-	8
MOR169-1	14	28	2	1	6	0	0	-	12	-	8
MOR162-5	16	35	3	2	8	1	2	-	12	-	8
MOR161-2	22	25	4	3	13	0	1	-	12	-	8
MOR283-3	18	11	1	3	5	6	3	-	12	-	2
MOR260-6	21	25	4	4	5	3	0	-	12	-	2
MOR170-1	18	22	0	2	7	0	1	-	12	-	8
MOR170-10	15	17	1	1	5	1	1	-	12	-	8
MOR161-5	16	30	5	3	13	1	0	-	12	-	8
MOR202-5	14	40	4	3	5	1	0	-	12	-	5
MOR283-2	18	10	2	3	5	3	3	-	12	-	2
MOR170-11	17	20	0	0	5	2	0	-	12	-	8
MOR261-12	20	11	5	3	6	0	0	-	12	-	2
MOR283-6	18	8	0	3	6	6	4	-	12	-	2
MOR283-9	19	10	1	4	5	6	4	-	12	-	2
MOR256-18	13	21	3	4	5	0	1	-	12	-	2
MOR225-14	15	17	3	3	6	2	2	-	12	-	4
MOR162-8	18	29	4	1	5	3	0	-	12	-	8
MOR162-2	13	31	3	0	10	2	4	-	12	-	8
MOR162-12	14	26	2	2	6	2	2	-	12	-	8
MOR225-12	13	16	3	2	5	1	2	-	12	-	4
MOR225-5	15	17	3	3	6	2	2	-	12	-	4
MOR225-15	15	16	3	3	6	2	2	-	12	-	4
MOR204-22	16	24	3	4	2	4	6	-	-	2	5
MOR204-36	14	22	3	2	2	1	6	-	-	2	5
MOR204-34	21	22	2	3	3	1	6	-	-	2	5
MOR204-14	19	28	2	3	2	1	6	-	-	2	5
MOR25-2	11	8	10	2	2	0	0	-	-	-	52
MOR28-1	15	11	7	4	1	0	0	-	-	-	52
MOR32-2	16	10	17	4	4	0	0	-	-	-	52
MOR39-1	12	3	1	4	0	0	0	-	-	-	52
MOR12-4	8	12	8	2	3	0	0	-	-	-	51
MOR30-3	7	9	9	2	1	0	0	-	-	-	52
MOR29-1	14	10	4	3	1	0	0	-	-	-	52
MOR19-1	13	8	3	4	3	0	0	-	-	-	51
MOR33-2	19	14	15	2	1	1	0	-	-	-	52
MOR15-3	10	7	5	4	3	1	0	-	-	-	51

MOR20-1	8	7	5	2	2	0	1	-	-	-	51
MOR31-10	13	12	13	3	2	0	1	-	-	-	52
MOR19-2	6	4	3	3	0	0	0	-	-	-	51
MOR262-12	28	36	1	4	4	2	0	-	-	-	13
MOR262-6	26	35	0	4	3	2	0	-	-	-	13
MOR11-2	10	5	5	4	3	0	0	-	-	-	51
MOR13-4	13	7	11	3	3	0	0	-	-	-	51
MOR13-1	15	6	4	4	3	1	0	-	-	-	51
MOR31-9	13	9	10	4	3	1	1	-	-	-	52
MOR258-6	19	27	1	1	4	0	0	-	-	-	13
MOR258-1	17	24	1	2	3	1	1	-	-	-	13
MOR38-1	7	8	3	1	2	0	0	-	-	-	52
MOR26-2	11	8	9	4	0	2	0	-	-	-	52
MOR18-3	11	5	11	3	1	1	0	-	-	-	51
MOR16-1	12	5	4	1	2	0	0	-	-	-	51
MOR135-28	19	31	6	4	3	3	0	-	-	-	1
MOR42-1	11	3	0	1	0	0	0	-	-	-	55
MOR42-3	11	4	0	1	0	0	0	-	-	-	55
MOR11-1	10	6	5	4	4	0	0	-	-	-	51
MOR15-4	11	7	4	4	3	1	0	-	-	-	51
MOR35-1	7	8	7	4	3	0	0	-	-	-	52
MOR12-1	12	7	9	4	2	0	0	-	-	-	51
MOR187-2	14	22	4	3	4	0	2	-	-	-	8
MOR150-1	21	27	3	2	0	6	0	-	-	-	7
MOR266-3	12	15	2	2	2	0	0	-	-	-	10
MOR127-1	19	26	1	4	3	1	0	-	-	-	1
MOR14-11	14	13	6	2	4	1	0	-	-	-	51
MOR264-6	20	30	4	4	4	0	1	-	-	-	10
MOR217-1	14	34	3	1	2	0	0	-	-	-	13
MOR218-1	16	11	3	3	0	0	2	-	-	-	5
MOR103-4	17	32	3	0	1	0	0	-	-	-	6
MOR103-2	15	28	3	0	2	0	0	-	-	-	6
MOR267-8	17	21	4	0	2	1	0	-	-	-	10
MOR186-1	16	25	2	4	5	0	0	-	-	-	8
MOR243-1	20	32	6	1	0	0	0	-	-	-	4
MOR174-9	16	33	6	3	2	0	1	-	-	-	5
MOR105-7	18	13	0	1	0	0	0	-	-	-	6
MOR101-1	17	16	1	0	2	0	0	-	-	-	2
MOR233-2	23	28	8	3	3	2	3	-	-	-	4
MOR213-3	14	26	8	2	0	0	0	-	-	-	9
MOR40-12	8	4	2	4	1	1	0	-	-	-	56
MOR10-4	13	7	7	4	2	0	0	-	-	-	51
MOR21-1	11	2	3	0	1	0	0	-	-	-	51
MOR223-2	12	19	1	4	4	4	0	-	-	-	10
MOR223-6	19	25	3	2	3	0	0	-	-	-	10
MOR104-2	10	30	1	1	2	0	0	-	-	-	11
MOR260-2	25	25	1	4	3	4	0	-	-	-	2
MOR244-3	18	28	8	3	0	0	0	-	-	-	4
MOR246-2	18	22	4	2	2	1	0	-	-	-	4
MOR165-6	20	32	3	2	4	1	4	-	-	-	8
MOR171-49	10	26	5	2	4	0	1	-	-	-	8
MOR171-53	18	21	5	2	4	0	1	-	-	-	8
MOR223-4	18	20	3	2	3	1	0	-	-	-	10
MOR222-1	12	17	0	0	2	0	1	-	-	-	9
MOR224-9	19	21	0	1	4	0	1	-	-	-	10
MOR268-1	16	23	1	4	2	1	1	-	-	-	10

MOR227-1	23	23	6	1	1	0	0	-	-	-	4
MOR223-9	18	26	3	2	2	0	0	-	-	-	10
MOR264-5	22	27	4	2	2	0	2	-	-	-	10
MOR218-3	12	11	3	1	1	0	1	-	-	-	5
MOR256-4	16	26	3	4	3	0	0	-	-	-	2
MOR266-4	19	12	3	2	3	0	0	-	-	-	10
MOR224-10	19	19	2	4	4	0	0	-	-	-	10
MOR264-19	26	21	4	1	3	0	1	-	-	-	10
MOR267-17	16	24	2	4	3	0	0	-	-	-	10
MOR175-2	25	26	6	2	1	0	0	-	-	-	5
MOR261-10	25	26	4	3	1	2	0	-	-	-	2
MOR202-7	12	39	3	3	4	1	0	-	-	-	5
MOR264-26	18	27	3	0	2	1	0	-	-	-	11
MOR172-7	13	26	0	1	1	0	0	-	-	-	5
MOR152-1	15	19	1	4	2	4	0	-	-	-	7
MOR252-3	17	10	2	0	3	0	0	-	-	-	13
MOR223-5	18	16	2	2	3	0	0	-	-	-	10
MOR258-3	20	21	3	2	2	0	0	-	-	-	13
MOR258-2	19	28	4	2	2	1	0	-	-	-	13
MOR172-1	21	40	8	0	1	0	0	-	-	-	5
MOR233-14	22	20	7	4	3	1	0	-	-	-	4
MOR233-10	23	24	5	4	3	2	2	-	-	-	4
MOR233-20	23	19	7	4	2	1	1	-	-	-	4
MOR228-5	19	20	5	4	1	1	0	-	-	-	4
MOR228-4	19	21	5	4	1	1	0	-	-	-	4
MOR248-2	18	14	3	1	3	1	2	-	-	-	4
MOR248-18	20	19	2	3	1	1	2	-	-	-	4
MOR246-4	21	22	4	2	3	1	0	-	-	-	4
MOR267-15	14	13	2	4	0	0	0	-	-	-	10
MOR267-3	22	20	3	2	3	0	0	-	-	-	10
MOR233-13	26	21	9	4	1	2	2	-	-	-	4
MOR146-1	19	18	2	1	0	2	3	-	-	-	7
MOR102-2	16	14	1	1	1	0	0	-	-	-	13
MOR170-9	9	17	1	1	4	1	0	-	-	-	8
MOR204-15	20	27	3	4	2	1	3	-	-	-	5
MOR202-43	16	19	3	1	1	0	0	-	-	-	5
MOR202-32	16	19	3	1	1	0	0	-	-	-	5
MOR248-11	17	15	5	4	3	0	1	-	-	-	4
MOR184-6	17	16	2	1	4	0	0	-	-	-	5
MOR160-4	18	19	1	0	0	0	0	-	-	-	8
MOR206-6	18	23	4	1	0	0	0	-	-	-	8
MOR174-1	20	33	3	3	0	0	0	-	-	-	5
MOR185-7	20	19	1	3	1	0	1	-	-	-	8
MOR190-2	21	22	4	4	3	0	0	-	-	-	8
MOR194-1	24	29	4	4	3	1	0	-	-	-	8
MOR185-1	17	31	8	3	4	0	1	-	-	-	8
MOR182-2	13	23	4	3	4	2	1	-	-	-	5
MOR146-9	15	21	1	2	1	2	3	-	-	-	7
MOR257-5	25	24	3	1	2	1	0	-	-	-	2
MOR196-1	20	25	2	2	0	0	0	-	-	-	5
MOR215-1	20	36	5	2	1	0	0	-	-	-	5
MOR195-1	15	32	5	3	0	0	0	-	-	-	5
MOR196-4	25	33	4	2	0	0	0	-	-	-	5
MOR175-3	21	28	10	3	0	1	0	-	-	-	5
MOR215-3	19	29	7	2	1	0	0	-	-	-	5
MOR174-11	15	25	3	1	2	0	0	-	-	-	5

MOR210-2	20	28	6	0	1	0	0	-	-	-	9
MOR215-2	16	26	7	1	0	0	0	-	-	-	5
MOR259-9	15	21	3	1	3	1	1	-	-	-	10
MOR259-12	17	25	3	0	4	2	0	-	-	-	10
MOR203-1	20	26	4	2	1	0	0	-	-	-	5
MOR184-4	16	16	3	0	4	0	0	-	-	-	5
MOR259-11	19	19	3	2	4	2	0	-	-	-	10
MOR178-1	19	28	6	2	2	0	0	-	-	-	5
MOR214-5	17	30	6	2	1	0	1	-	-	-	5
MOR136-14	26	27	0	4	3	1	0	-	-	-	1
MOR135-1	21	31	3	4	2	2	1	-	-	-	1
MOR206-2	18	24	4	1	1	0	1	-	-	-	8
MOR188-4	16	28	3	4	3	0	0	-	-	-	8
MOR206-3	20	29	3	1	1	0	0	-	-	-	8
MOR135-6	22	35	2	3	3	3	1	-	-	-	1
MOR255-4	22	21	1	1	3	1	1	-	-	-	3
MOR173-2	20	30	8	2	1	0	1	-	-	-	9
MOR181-2	11	25	4	1	1	3	0	-	-	-	5
MOR172-5	21	36	2	1	1	0	2	-	-	-	5
MOR213-6	13	21	6	0	6	0	0	-	-	-	9
MOR203-2	22	23	2	1	2	1	0	-	-	-	5
MOR267-4	19	18	2	0	2	0	0	-	-	-	10
MOR248-15	19	13	2	1	4	1	1	-	-	-	4
MOR177-9	15	30	5	1	3	1	0	-	-	-	8
MOR176-3	15	31	3	2	3	0	0	-	-	-	5
MOR214-2	13	32	7	1	2	0	1	-	-	-	5
MOR204-5	16	32	2	3	3	0	1	-	-	-	5
MOR271-1	17	17	3	1	1	2	0	-	-	-	2
MOR204-3	23	31	4	4	1	0	1	-	-	-	5
MOR204-1	15	17	2	2	0	0	1	-	-	-	5
MOR204-4	19	30	4	4	3	0	1	-	-	-	5
MOR214-3	17	26	5	2	2	0	1	-	-	-	5
MOR227-5	23	23	5	1	0	0	0	-	-	-	4
MOR236-1	20	19	5	4	2	1	0	-	-	-	4
MOR145-1	24	25	3	3	4	2	1	-	-	-	7
MOR147-3	22	20	0	3	2	2	0	-	-	-	7
MOR152-4	22	23	2	2	1	2	0	-	-	-	7
MOR262-7	17	24	1	2	3	1	1	-	-	-	13
MOR147-1	22	19	2	4	0	2	0	-	-	-	7
MOR151-1	26	24	3	3	0	0	1	-	-	-	7
MOR143-1	15	21	1	2	2	2	0	-	-	-	7
MOR262-2	22	26	1	1	4	0	0	-	-	-	13
MOR106-12	16	23	3	1	4	1	0	-	-	-	11
MOR138-6	24	31	4	1	3	0	0	-	-	-	1
MOR154-1	24	29	3	2	2	4	0	-	-	-	7
MOR174-6	16	19	3	0	2	0	0	-	-	-	5
MOR172-2	17	33	4	1	2	0	2	-	-	-	5
MOR248-9	16	14	5	2	4	0	1	-	-	-	4
MOR279-2	21	11	3	2	3	1	0	-	-	-	2
MOR281-1	20	22	3	2	2	0	0	-	-	-	2
MOR224-12	17	13	0	2	4	0	0	-	-	-	10
MOR175-5	18	25	4	1	0	0	0	-	-	-	5
MOR201-2	20	37	7	4	1	0	0	-	-	-	5
MOR175-4	23	26	2	0	2	0	0	-	-	-	5
MOR209-1	16	32	4	1	1	1	1	-	-	-	9
MOR133-6	19	22	2	4	2	0	0	-	-	-	1

MOR129-2	21	32	3	2	3	0	0	-	-	-	1
MOR105-5	22	15	0	1	2	1	0	-	-	-	6
MOR135-8	17	31	3	4	2	3	2	-	-	-	1
MOR267-19	18	20	5	1	2	0	0	-	-	-	10
MOR104-1	21	20	0	0	2	1	0	-	-	-	6
MOR225-11	22	13	8	3	2	1	0	-	-	-	4
MOR223-8	13	24	4	1	3	0	0	-	-	-	10
MOR174-3	19	32	4	2	3	0	0	-	-	-	5
MOR177-10	8	11	4	1	2	1	0	-	-	-	8
MOR121-1	22	17	2	0	1	0	0	-	-	-	11
MOR160-2	19	24	4	2	0	1	1	-	-	-	8
MOR255-6	19	20	1	1	3	1	1	-	-	-	3
MOR262-10	18	22	2	3	1	0	0	-	-	-	13
MOR138-2	28	29	3	3	1	0	1	-	-	-	1
MOR160-1	23	21	3	1	0	3	0	-	-	-	8
MOR260-10	25	28	1	3	3	2	0	-	-	-	2
MOR177-1	19	28	6	4	4	3	1	-	-	-	8
MOR177-6	18	29	5	1	4	0	0	-	-	-	8
MOR208-3	15	24	7	2	4	1	1	-	-	-	9
MOR260-4	21	25	1	4	3	0	0	-	-	-	2
MOR260-3	24	21	1	2	2	0	0	-	-	-	2
MOR174-16	16	21	3	0	2	1	0	-	-	-	5
MOR211-2	21	28	4	1	3	0	2	-	-	-	9
MOR174-13	16	20	3	1	3	0	0	-	-	-	5
MOR174-2	19	32	2	3	3	0	1	-	-	-	5
MOR212-1	18	26	1	2	1	0	0	-	-	-	9
MOR212-5	18	24	2	2	1	0	0	-	-	-	9
MOR216-1	15	22	0	1	1	0	0	-	-	-	5
MOR260-7	24	21	6	3	4	3	0	-	-	-	2
MOR192-1	19	32	4	3	3	0	0	-	-	-	8
MOR179-5	12	21	8	1	2	0	1	-	-	-	5
MOR173-1	20	27	8	2	1	0	1	-	-	-	9
MOR264-23	23	26	4	3	3	0	2	-	-	-	10
MOR174-5	19	13	2	2	2	0	0	-	-	-	5
MOR245-14	15	22	8	3	3	1	0	-	-	-	5
MOR245-25	17	22	8	3	1	1	0	-	-	-	5
MOR36-1	12	5	8	2	4	0	0	-	-	-	52
MOR218-8	19	9	0	1	0	0	0	-	-	-	5
MOR213-4	15	28	7	1	2	0	0	-	-	-	9
MOR127-4	23	20	1	3	3	0	0	-	-	-	1
MOR160-5	15	22	5	3	1	5	0	-	-	-	8
MOR135-27	18	27	4	4	3	3	1	-	-	-	1
MOR135-10	22	35	3	3	3	2	0	-	-	-	1
MOR225-3	17	15	6	2	2	0	0	-	-	-	4
MOR103-14	14	33	4	0	0	0	0	-	-	-	6
MOR130-2	20	20	0	1	3	0	0	-	-	-	1
MOR257-10	24	23	4	1	2	0	0	-	-	-	2
MOR260-5	24	32	2	4	1	0	0	-	-	-	2
MOR227-3	23	21	10	1	0	0	0	-	-	-	4
MOR30-1	10	10	9	4	1	0	0	-	-	-	52
MOR261-2	17	18	1	2	3	1	1	-	-	-	2
MOR135-26	18	30	3	3	4	3	2	-	-	-	1
MOR183-1	17	21	5	1	4	1	0	-	-	-	5
MOR135-7	18	34	2	4	4	2	1	-	-	-	1
MOR263-10	16	20	2	0	0	0	0	-	-	-	10
MOR245-8	20	9	4	3	2	0	1	-	-	-	4

MOR41-1	16	7	0	1	2	0	0	-	-	-	52
MOR283-8	18	10	0	4	4	1	2	-	-	-	2
MOR106-6	18	23	5	2	2	2	3	-	-	-	11
MOR106-14	14	24	3	3	3	0	5	-	-	-	11
MOR118-1	20	24	0	2	1	0	0	-	-	-	6
MOR244-1	21	18	6	0	2	0	0	-	-	-	4
MOR256-62	17	18	5	3	1	0	0	-	-	-	2
MOR42-2	20	3	0	1	1	0	0	-	-	-	55
MOR25-1	11	6	8	3	4	0	0	-	-	-	52
MOR40-3	11	4	1	4	1	1	0	-	-	-	56
MOR155-1	19	22	3	1	1	2	1	-	-	-	7
MOR259-3	18	19	4	2	4	2	0	-	-	-	10
MOR267-20	16	24	2	4	3	0	0	-	-	-	10
MOR267-1	14	11	0	3	4	0	0	-	-	-	10
MOR254-2	21	25	2	0	3	0	2	-	-	-	3
MOR248-5	20	16	6	2	3	2	1	-	-	-	4
MOR267-16	18	22	3	2	4	0	0	-	-	-	10
MOR256-39	17	20	1	3	1	0	0	-	-	-	2
MOR261-1	30	23	1	0	0	2	0	-	-	-	2
MOR103-17	20	28	4	1	1	0	0	-	-	-	6
MOR40-16	14	3	1	4	1	0	0	-	-	-	56
MOR232-8	17	15	2	1	1	0	1	-	-	-	10
MOR241-2	14	15	6	3	0	0	0	-	-	-	4
MOR172-3	18	29	5	2	3	0	2	-	-	-	5
MOR221-4	18	20	3	1	1	0	2	-	-	-	5
MOR222-3	15	18	1	1	1	0	0	-	-	-	9
MOR206-1	19	23	2	1	1	0	0	-	-	-	8
MOR101-2	16	15	1	1	4	0	0	-	-	-	2
MOR256-29	18	20	2	3	0	0	0	-	-	-	2
MOR145-4	21	17	1	2	2	0	0	-	-	-	7
MOR135-4	21	29	4	2	3	1	1	-	-	-	1
MOR241-1	16	18	5	4	0	0	0	-	-	-	4
MOR159-3	20	19	1	0	1	0	0	-	-	-	12
MOR146-2	21	21	1	2	1	2	3	-	-	-	7
MOR219-5	15	16	4	0	1	2	0	-	-	-	5
MOR219-2	16	14	4	1	1	2	0	-	-	-	5
MOR219-4	11	15	3	0	0	2	0	-	-	-	5
MOR219-3	15	14	3	0	1	1	0	-	-	-	5
MOR251-4	13	19	2	0	2	2	1	-	-	-	13
MOR203-7	17	22	4	1	3	0	0	-	-	-	5
MOR207-1	15	27	3	4	2	0	0	-	-	-	8
MOR192-3	19	34	5	2	4	0	0	-	-	-	8
MOR212-3	20	24	6	1	2	0	0	-	-	-	9
MOR182-6	18	21	4	1	3	1	1	-	-	-	5
MOR255-3	21	25	1	1	3	3	0	-	-	-	3
MOR210-4	16	30	2	0	4	0	1	-	-	-	9
MOR146-8	19	18	2	2	1	2	2	-	-	-	7
MOR148-1	18	22	2	3	0	2	1	-	-	-	7
MOR261-5	26	24	4	3	0	2	0	-	-	-	2
MOR173-3	20	17	7	1	2	0	1	-	-	-	9
MOR129-3	22	32	1	1	2	0	0	-	-	-	1
MOR197-1	26	20	2	4	0	0	0	-	-	-	5
MOR106-1	24	24	1	0	2	0	0	-	-	-	11
MOR213-9	17	21	3	0	2	0	0	-	-	-	9
MOR202-6	9	24	4	2	4	3	2	-	-	-	5
MOR174-8	20	26	7	1	1	0	0	-	-	-	5

MOR106-2	15	27	4	2	2	1	4	-	-	-	11
MOR177-2	18	25	4	1	4	3	1	-	-	-	8
MOR177-5	15	28	10	2	4	2	1	-	-	-	8
MOR127-5	20	22	5	4	1	0	1	-	-	-	1
MOR220-2	17	19	2	1	1	0	2	-	-	-	5
MOR221-2	18	16	2	1	1	0	2	-	-	-	5
MOR175-1	24	27	6	4	1	0	0	-	-	-	5
MOR211-9	17	26	3	0	2	0	1	-	-	-	9
MOR262-1	22	24	1	2	4	1	0	-	-	-	2
MOR203-4	21	28	1	2	2	1	0	-	-	-	5
MOR219-1	18	13	3	0	0	2	0	-	-	-	5
MOR103-18	20	19	3	0	2	0	0	-	-	-	6
MOR150-3	21	13	0	4	0	0	1	-	-	-	7
MOR251-2	16	12	3	1	3	2	2	-	-	-	13
MOR129-1	24	30	5	2	2	0	0	-	-	-	1
MOR246-6	18	15	4	4	2	0	1	-	-	-	4
MOR264-7	22	20	7	2	4	0	1	-	-	-	10
MOR160-3	17	17	1	1	0	2	0	-	-	-	8
MOR261-9	24	27	5	1	1	1	0	-	-	-	2
MOR224-4	18	17	3	0	2	0	0	-	-	-	10
MOR266-9	19	18	3	0	2	0	1	-	-	-	10
MOR119-2	11	13	1	1	2	0	0	-	-	-	6
MOR174-14	19	28	8	1	1	0	0	-	-	-	5
MOR122-3	19	28	2	1	0	0	1	-	-	-	11
MOR104-4	22	21	5	1	0	0	0	-	-	-	6
MOR254-1	21	26	3	0	3	0	2	-	-	-	3
MOR264-21	22	27	1	4	0	0	1	-	-	-	10
MOR156-5	22	16	3	0	0	1	1	-	-	-	1
MOR156-3	24	15	2	0	1	1	1	-	-	-	1
MOR249-2	14	24	4	1	1	0	0	-	-	-	5
MOR267-2	18	20	2	2	2	0	0	-	-	-	10
MOR147-2	25	21	0	4	1	2	1	-	-	-	7
MOR179-7	11	27	10	1	3	1	1	-	-	-	5
MOR175-9	22	28	3	0	2	0	0	-	-	-	5
MOR135-3	21	25	3	2	3	3	1	-	-	-	1
MOR172-4	17	32	4	2	3	0	2	-	-	-	5
MOR202-19	12	33	8	3	4	2	2	-	-	-	5
MOR255-2	22	18	2	1	4	1	0	-	-	-	3
MOR255-1	25	19	1	0	3	0	0	-	-	-	3
MOR215-4	15	34	4	1	0	0	0	-	-	-	5
MOR261-13	22	20	3	2	2	0	1	-	-	-	2
MOR256-5	18	21	0	4	2	0	0	-	-	-	2
MOR106-16	24	24	1	0	1	0	0	-	-	-	11
MOR174-4	17	28	5	3	3	0	1	-	-	-	5
MOR214-4	17	22	5	1	3	0	1	-	-	-	5
MOR267-10	16	24	2	4	3	0	0	-	-	-	10
MOR149-1	20	18	1	1	1	1	1	-	-	-	7
MOR264-24	25	33	1	1	3	0	0	-	-	-	13
MOR251-1	17	10	3	1	1	1	1	-	-	-	13
MOR267-6	10	21	2	0	2	0	0	-	-	-	10
MOR141-3	21	33	2	4	3	1	0	-	-	-	7
MOR242-1	16	21	7	0	2	0	0	-	-	-	4
MOR266-8	19	22	5	3	2	0	1	-	-	-	10
MOR256-17	15	23	5	2	3	0	0	-	-	-	2
MOR225-1	20	11	5	3	2	1	0	-	-	-	4
MOR251-5	14	22	4	2	2	1	0	-	-	-	13

MOR149-3	16	19	2	4	0	2	0	-	-	-	7
MOR143-2	15	27	3	1	2	1	0	-	-	-	7
MOR146-3	18	22	2	2	1	2	3	-	-	-	7
MOR264-1	24	25	1	4	0	0	2	-	-	-	10
MOR145-2	18	28	1	1	1	1	0	-	-	-	7
MOR258-5	23	24	3	3	1	1	1	-	-	-	13
MOR262-9	26	40	1	2	2	0	0	-	-	-	13
MOR145-3	21	21	1	1	2	1	0	-	-	-	7
MOR264-2	18	25	5	3	3	0	1	-	-	-	10
MOR264-18	16	21	4	1	1	0	2	-	-	-	10
MOR152-2	15	16	1	4	3	2	0	-	-	-	7
MOR126-1	24	20	2	1	0	0	0	-	-	-	1
MOR267-18	15	24	2	4	3	0	0	-	-	-	10
MOR155-2	15	17	1	1	1	2	0	-	-	-	7
MOR227-6	15	23	3	1	2	0	2	-	-	-	5
MOR125-1	17	16	1	4	1	0	0	-	-	-	1
MOR135-9	26	32	3	1	4	2	2	-	-	-	1
MOR145-6	20	21	1	2	2	1	0	-	-	-	7
MOR103-1	18	23	2	1	0	0	0	-	-	-	6
MOR257-3	18	16	3	2	0	0	0	-	-	-	2
MOR138-3	19	22	3	4	3	0	1	-	-	-	1
MOR122-1	17	24	4	1	0	0	0	-	-	-	11
MOR233-1	23	26	7	3	3	0	3	-	-	-	4
MOR156-1	25	15	4	0	0	1	1	-	-	-	1
MOR240-2	16	17	5	2	2	1	0	-	-	-	4
MOR119-1	17	13	2	1	2	0	0	-	-	-	6
MOR103-10	15	22	1	1	1	0	0	-	-	-	6
MOR103-5	19	25	0	0	0	0	0	-	-	-	6
MOR256-48	18	20	0	4	2	0	0	-	-	-	2
MOR273-2	14	16	2	2	2	0	0	-	-	-	2
MOR285-2	19	11	2	2	0	0	0	-	-	-	2
MOR233-3	22	25	8	3	2	2	2	-	-	-	4
MOR233-8	25	21	7	2	1	2	1	-	-	-	4
MOR279-1	18	11	3	2	2	1	1	-	-	-	2
MOR260-1	26	29	1	4	3	2	0	-	-	-	2
MOR248-6	20	16	5	1	4	1	1	-	-	-	4
MOR267-5	17	21	2	0	3	0	0	-	-	-	10
MOR282-1	18	17	1	3	1	0	0	-	-	-	2
MOR220-3	15	15	2	1	2	0	3	-	-	-	5
MOR261-11	21	15	0	1	3	0	1	-	-	-	2
MOR261-6	29	20	1	0	1	0	0	-	-	-	2
MOR266-6	12	16	1	2	2	0	1	-	-	-	10
MOR270-1	15	19	3	1	1	2	0	-	-	-	2
MOR280-1	15	8	1	3	1	0	1	-	-	-	2
MOR256-23	13	24	3	2	2	0	2	-	-	-	2
MOR256-14	19	19	4	4	1	1	0	-	-	-	2
MOR264-11	24	20	5	1	3	0	1	-	-	-	10
MOR103-16	16	27	0	0	3	0	0	-	-	-	6
MOR128-2	21	22	6	2	1	1	0	-	-	-	1
MOR103-15	21	34	2	0	1	0	0	-	-	-	6
MOR210-1	20	29	6	0	1	0	0	-	-	-	9
MOR179-4	14	29	10	2	2	2	0	-	-	-	5
MOR283-7	20	8	2	1	3	6	4	-	-	-	2
MOR257-4	19	26	1	0	2	0	0	-	-	-	2
MOR268-5	16	23	1	4	3	2	3	-	-	-	10
MOR120-1	12	16	4	0	0	0	1	-	-	-	9

MOR106-9	16	23	3	1	4	1	0	-	-	-	11
MOR158-1	7	18	1	3	2	0	0	-	-	-	1
MOR133-1	20	16	1	4	0	0	0	-	-	-	1
MOR248-8	19	16	7	2	3	1	2	-	-	-	4
MOR272-1	17	20	2	0	1	2	1	-	-	-	2
MOR276-2	18	11	0	3	1	0	0	-	-	-	2
MOR284-2	18	11	0	0	1	0	1	-	-	-	2
MOR206-4	17	29	4	0	1	0	0	-	-	-	8
MOR202-9	19	19	4	1	3	0	0	-	-	-	5
MOR282-2	18	15	1	3	1	0	0	-	-	-	2
MOR283-11	15	12	2	0	2	6	1	-	-	-	2
MOR283-4	17	12	2	2	3	5	3	-	-	-	2
MOR250-4	22	13	1	0	2	0	0	-	-	-	12
MOR239-5	20	13	4	4	1	0	0	-	-	-	4
MOR266-1	21	23	4	3	2	0	1	-	-	-	10
MOR246-1	16	20	6	2	1	0	1	-	-	-	4
MOR266-5	19	17	2	1	3	1	0	-	-	-	10
MOR263-5	17	31	4	2	2	0	0	-	-	-	10
MOR283-1	19	10	2	2	3	6	1	-	-	-	2
MOR283-12	18	11	1	4	4	7	4	-	-	-	2
MOR122-2	20	25	1	0	0	0	0	-	-	-	11
MOR263-12	16	30	4	2	2	0	0	-	-	-	10
MOR263-9	16	21	2	2	0	0	0	-	-	-	10
MOR212-2	17	26	5	1	2	0	0	-	-	-	9
MOR179-6	15	34	8	3	3	0	0	-	-	-	5
MOR283-5	13	9	1	2	3	5	1	-	-	-	2
MOR256-63	12	21	1	3	1	0	1	-	-	-	2
MOR239-6	17	17	8	3	3	0	0	-	-	-	4
MOR179-2	16	28	8	2	3	1	0	-	-	-	5
MOR222-2	14	23	0	0	2	0	1	-	-	-	9
MOR102-1	13	22	2	0	2	0	0	-	-	-	13
MOR170-14	16	17	4	0	3	0	0	-	-	-	8
MOR159-4	21	22	1	0	0	0	0	-	-	-	12
MOR233-15	21	26	6	4	2	2	2	-	-	-	4
MOR283-10	18	15	1	1	4	4	1	-	-	-	2
MOR267-14	12	9	4	2	0	1	0	-	-	-	10
MOR256-54	8	22	2	3	4	0	2	-	-	-	2
MOR256-22	11	26	6	3	4	0	1	-	-	-	2
MOR256-1	16	31	2	4	2	0	1	-	-	-	2
MOR256-58	10	23	4	3	3	0	1	-	-	-	2
MOR256-57	11	24	4	3	2	0	1	-	-	-	2
MOR256-26	11	24	3	3	3	0	1	-	-	-	2
MOR251-3	15	22	4	2	0	1	0	-	-	-	13
MOR239-3	18	15	4	3	1	1	0	-	-	-	4
MOR123-1	15	13	1	1	2	0	0	-	-	-	10
MOR123-2	13	16	0	1	2	0	0	-	-	-	10
MOR105-10	18	17	2	0	1	0	0	-	-	-	6
MOR106-3	18	28	6	3	3	2	4	-	-	-	11
MOR256-20	19	19	1	2	1	0	0	-	-	-	2
MOR248-19	19	15	3	2	4	1	2	-	-	-	4
MOR248-20	19	15	3	2	4	1	2	-	-	-	4
MOR245-9	16	13	4	3	3	1	0	-	-	-	4
MOR177-4	14	29	10	2	4	1	0	-	-	-	8
MOR106-5	16	19	2	1	4	0	0	-	-	-	11
MOR105-1	24	26	1	1	3	0	0	-	-	-	6
MOR220-1	18	16	2	0	1	0	2	-	-	-	5

MOR245-13	13	19	1	2	4	1	0	-	-	-	4
MOR105-2	19	13	0	1	2	1	0	-	-	-	6
MOR233-5	26	26	11	3	2	2	2	-	-	-	4
MOR218-2	16	13	4	1	0	0	3	-	-	-	5
MOR120-2	11	12	4	0	0	0	0	-	-	-	9
MOR233-21	21	20	10	4	1	1	1	-	-	-	4
MOR257-9	18	21	0	1	2	1	0	-	-	-	2
MOR266-10	23	14	3	1	2	0	0	-	-	-	10
MOR218-10	13	17	4	1	0	0	2	-	-	-	5
MOR205-2	16	16	4	0	1	0	0	-	-	-	5
MOR285-5	19	13	1	2	1	1	0	-	-	-	2
MOR256-49	8	18	2	4	1	0	1	-	-	-	2
MOR222-4	12	22	0	0	1	0	1	-	-	-	9
MOR269-2	14	15	2	0	2	0	1	-	-	-	10
MOR105-4	19	15	0	0	1	0	0	-	-	-	6
MOR106-15	16	21	5	2	2	2	3	-	-	-	11
MOR218-12	18	12	4	3	0	0	3	-	-	-	5
MOR218-13	16	13	4	3	0	0	2	-	-	-	5
MOR262-4	20	29	5	3	3	0	0	-	-	-	13
MOR268-3	15	27	1	4	4	3	2	-	-	-	10
MOR105-11	20	13	0	1	2	0	0	-	-	-	6
MOR225-7	14	14	8	1	1	0	0	-	-	-	4
MOR231-5	20	12	3	4	0	3	1	-	-	-	4
MOR218-11	13	13	4	3	0	0	2	-	-	-	5
MOR157-1	10	12	1	1	1	0	0	-	-	-	1
MOR245-2	16	11	4	2	4	0	0	-	-	-	4
MOR250-1	22	19	2	0	1	0	0	-	-	-	12
MOR268-4	18	22	1	3	0	4	2	-	-	-	10
MOR267-7	2	19	0	1	2	0	1	-	-	-	10
MOR202-41	16	19	3	1	1	0	0	-	-	-	5
MOR248-1	20	21	4	3	3	1	2	-	-	-	4
MOR268-6	7	16	1	1	2	1	0	-	-	-	10
MOR204-20	8	21	1	3	3	0	0	-	-	-	5
MOR218-9	12	16	4	3	0	0	2	-	-	-	5
MOR269-1	18	17	1	1	1	0	0	-	-	-	10
MOR124-1	18	10	0	1	1	0	0	-	-	-	2
MOR241-3	15	17	5	2	1	1	0	-	-	-	4
MOR246-5	21	14	2	2	4	0	0	-	-	-	4
MOR245-7	19	11	6	4	4	1	0	-	-	-	4
MOR247-1	16	12	2	1	2	0	0	-	-	-	4
