# Supplementary material for: Common peptides shed light on evolution of Olfactory Receptors
Source: BMC Evol Biol. 2009 May 5;9:91. doi: 10.1186/1471-2148-9-91 (PMC2681464; doi:10.1186/1471-2148-9-91)
Supplement: Additional file 9 — Human ORs CP numbers and cluster assignment. Number of CPs from each ancestor occurring in each Human OR and cluster assignment for each Human OR. [file 1471-2148-9-91-S9.pdf]

Legend

A	Number of A1 CPs
B	Number of A2 novel CPs
C	Number of A3 novel CPs
D	Number of A4 novel CPs
E	Number of A5 novel CPs
F	Number of A6 novel CPs
G	Number of human novel CPs
H	Cluster number A4 novel CPs
I	Cluster number A5 novel CPs
J	Family

Name	A	B	C	D	E	F	G	H	I	J
OR1E1	17	31	4	6	3	3	0	1	-	1
OR1E2	15	28	5	6	3	3	0	1	-	1
OR2D2	26	22	1	5	4	2	1	1	-	2
OR2D3	18	25	4	5	3	4	0	1	-	2
OR5M8	18	24	6	7	2	0	0	1	-	5
OR6C76	19	22	4	10	5	2	0	2	4	6
OR6C2	22	16	2	6	10	3	0	2	4	6
OR2AP1	22	22	1	11	1	0	0	2	-	2
OR6C4	24	19	5	11	5	0	0	2	-	6
OR6C6	23	15	2	7	11	1	0	2	-	6
OR6C74	19	24	2	7	6	3	0	2	-	6
OR6C75	19	20	5	9	4	2	0	2	-	6
OR6C70	17	15	2	10	4	2	0	2	-	6
OR6C68	17	14	4	6	9	1	0	2	-	6
OR6C65	20	17	3	9	4	1	0	2	-	6
OR6C1	24	23	2	7	2	0	0	2	-	6
OR6C3	20	25	3	8	7	0	0	2	-	6
OR51B5	16	2	6	7	7	0	0	3	-	51
OR51B6	12	3	7	5	3	0	0	3	-	51
OR51B4	9	1	5	5	2	0	0	3	-	51
OR51I2	15	9	5	7	3	0	0	3	-	51
OR2B11	19	14	3	6	1	0	0	4	-	2
OR2B6	22	27	0	6	2	0	0	4	-	2
OR2G3	19	31	2	5	2	0	0	4	-	2
OR2J1P	12	21	1	7	2	1	0	4	-	2
OR2J3	12	21	4	5	3	1	0	4	-	2
OR2B2	21	28	0	7	3	0	0	4	-	2
OR1L4	18	27	3	6	2	0	0	5	-	1
OR1L6	19	26	4	6	2	0	0	5	-	1
OR4C11	20	12	8	12	4	2	0	5	-	4
OR5M9	13	20	7	7	2	0	0	5	-	5
OR7D2	24	34	3	7	0	1	0	5	-	7
OR7G3	20	21	6	6	2	1	0	5	-	7
OR7C2	19	32	4	8	2	1	0	5	-	7
OR7C1	23	32	2	6	4	1	0	5	-	7
OR7A5	22	31	4	11	9	2	0	5	-	7
OR7A10	22	36	5	8	6	5	0	5	-	7
OR7A17	25	29	3	9	5	5	0	5	-	7

OR4A47	22	20	6	6	1	3	0	6	-	4
OR4C3	19	13	6	5	2	1	0	6	-	4
OR4A15	21	16	4	10	2	1	0	6	-	4
OR4A16	16	9	3	6	1	2	0	6	-	4
OR4D9	19	19	8	5	1	2	0	6	-	4
OR4D11	12	12	6	5	0	0	1	6	-	4
OR4C6	27	17	6	5	0	0	0	6	-	4
OR4C12	18	18	5	10	2	0	0	6	-	4
OR4C13	20	22	4	7	2	0	0	6	-	4
OR4C15	25	16	4	7	1	0	0	6	-	4
OR4C16	17	16	4	5	1	1	0	6	-	4
OR2G6	20	28	5	7	3	1	0	7	-	2
OR5R1	14	35	5	7	2	0	0	7	-	5
OR5M10	22	30	0	2	0	0	0	7	-	5
OR8U9	17	34	5	5	3	1	0	7	-	8
OR51A2	7	9	5	5	3	0	0	8	-	51
OR51A4	10	10	5	5	4	0	0	8	-	51
OR51E1	19	7	5	6	2	0	0	8	-	51
OR51E2	13	5	3	5	1	0	0	8	-	51
OR51G1	8	8	9	5	5	1	0	8	-	51
OR52N1	4	5	3	5	5	1	0	8	-	52
OR52E4	19	9	17	5	4	0	0	8	-	52
OR52E5	16	7	13	5	5	1	0	8	-	52
OR52N4	9	4	10	8	4	0	0	8	-	52
OR52N5	10	10	4	6	4	0	0	8	-	52
OR52H1	10	11	6	7	8	1	0	8	-	52
OR52B2	13	9	8	8	5	0	0	8	-	52
OR52K1	13	14	6	5	1	0	0	8	-	52
OR52K2	14	15	7	7	2	0	0	8	-	52
OR52A5	11	6	11	10	2	0	0	8	-	52
OR10H5	17	22	1	5	3	0	0	9	-	10
OR10H1	17	22	1	5	3	0	0	9	-	10
OR8K3	14	25	5	5	6	0	0	10	3	8
OR8G1P	17	35	5	6	12	2	0	10	3	8
OR1N1	24	22	0	8	1	0	0	10	-	1
OR1S2	14	25	0	6	1	0	0	10	-	1
OR1D2	16	21	3	5	1	0	0	10	-	1
OR1L3	19	25	3	5	0	0	0	10	-	1
OR5P2	19	27	2	6	3	0	0	10	-	5
OR8K1	21	24	2	5	4	1	0	10	-	8
OR8D1	16	31	6	8	2	0	0	10	-	8
OR56A1	16	3	4	10	5	0	0	11	-	56
OR56A4	16	2	4	8	8	0	1	11	-	56
OR56A5	11	2	2	10	7	0	0	11	-	56
OR56A3	13	2	3	7	5	0	0	11	-	56
OR56B1	18	2	2	7	1	1	0	11	-	56
OR2T29	20	24	1	7	7	0	2	12	5	2
OR2T1	19	25	1	7	9	0	0	12	5	2
OR2T5	20	24	1	7	7	0	2	12	5	2
OR2T12	13	15	2	5	1	1	1	12	-	2
OR2T27	18	25	0	5	3	0	0	12	-	2
OR2T4	20	23	0	8	3	1	2	12	-	2
OR2T6	18	18	0	5	2	0	0	12	-	2
OR2T11	16	24	2	7	3	0	0	12	-	2
OR4C46	20	16	6	9	2	0	0	12	-	4
OR4C5	17	29	8	7	0	1	0	12	-	4

OR4L1	13	16	5	5	4	0	0	12	-	4
OR4A5	21	16	5	6	0	2	0	12	-	4
OR4P4	18	15	3	6	2	1	0	12	-	4
OR2T35	17	30	2	4	6	1	0	-	1	2
OR2T2	17	30	2	4	6	1	0	-	1	2
OR10R2	11	28	3	1	5	0	1	-	1	10
OR11H12	8	22	2	2	8	0	1	-	1	11
OR11H2	8	22	3	2	8	0	1	-	1	11
OR11H1	8	22	2	2	8	0	1	-	1	11
OR4F29	12	12	4	3	7	2	0	-	2	4
OR4F5	19	15	5	4	5	0	0	-	2	4
OR4F15	14	10	5	3	5	2	0	-	2	4
OR4F16	12	12	4	3	7	2	0	-	2	4
OR4F3	12	12	4	3	7	2	0	-	2	4
OR4F17	21	15	5	4	5	0	0	-	2	4
OR4F4	21	15	5	4	5	0	0	-	2	4
OR4F21	12	11	4	3	7	2	0	-	2	4
OR5K4	16	13	1	1	5	0	0	-	3	5
OR5K1	19	17	1	0	5	0	0	-	3	5
OR8D4	16	22	3	3	6	0	0	-	3	8
OR8B8	22	29	6	2	8	1	0	-	3	8
OR8B3	15	30	4	1	21	0	0	-	3	8
OR8D2	16	24	4	4	5	2	0	-	3	8
OR8B2	14	26	4	1	20	0	0	-	3	8
OR8A1	19	38	5	3	5	0	0	-	3	8
OR8B12	18	34	6	3	8	0	0	-	3	8
OR8G5	16	28	8	4	14	1	0	-	3	8
OR5B21	21	40	3	2	6	0	0	-	4	5
OR5B12	14	36	7	1	7	1	1	-	4	5
OR5B2	17	23	5	2	8	0	0	-	4	5
OR5B3	15	21	6	3	10	0	0	-	4	5
OR5B17	9	23	8	2	9	1	0	-	4	5
OR2T34	17	16	1	1	5	2	0	-	5	2
OR2T3	16	14	1	1	6	2	1	-	5	2
OR1D5	15	16	3	2	0	0	0	-	-	1
OR1F12	23	20	1	2	1	1	0	-	-	1
OR1M1	21	23	2	2	3	0	0	-	-	1
OR1F1	21	34	2	2	0	0	1	-	-	1
OR1S1	9	32	0	5	1	0	0	-	-	1
OR1L8	27	23	1	3	2	0	0	-	-	1
OR1K1	14	27	2	3	1	0	0	-	-	1
OR1J2	25	28	1	2	4	0	0	-	-	1
OR1J1	25	26	0	0	3	0	0	-	-	1
OR1N2	24	19	2	3	2	0	0	-	-	1
OR1J4	26	26	1	4	4	0	0	-	-	1
OR1I1	14	16	0	2	1	0	0	-	-	1
OR1Q1	15	22	4	1	1	0	0	-	-	1
OR1B1	7	12	1	1	4	1	0	-	-	1
OR1L1	23	28	4	2	3	0	0	-	-	1
OR1C1	17	25	3	1	1	0	0	-	-	1
OR1D4	17	14	3	2	0	0	0	-	-	1
OR1A1	20	22	0	4	1	0	0	-	-	1
OR1A2	17	16	0	4	0	0	0	-	-	1
OR1G1	19	33	3	3	1	1	1	-	-	1
OR2M3	17	19	1	2	1	3	1	-	-	2
OR2M7	17	16	2	2	1	3	2	-	-	2

OR2A42	25	24	4	3	2	4	0	-	-	2
OR2T33	14	17	2	4	1	1	1	-	-	2
OR2Y1	13	30	4	2	5	0	0	-	-	2
OR2H1	15	20	2	3	3	0	0	-	-	2
OR2AG1	17	11	1	2	2	5	0	-	-	2
OR2AG2	20	10	1	2	2	5	0	-	-	2
OR2F2	21	19	4	0	3	0	1	-	-	2
OR2A4	15	16	1	2	2	3	0	-	-	2
OR2V2	18	15	1	2	1	2	0	-	-	2
OR2V1	18	13	2	3	1	2	0	-	-	2
OR2L3	15	17	4	2	2	3	0	-	-	2
OR2L2	18	19	1	1	3	2	0	-	-	2
OR2L8	16	18	5	1	2	4	0	-	-	2
OR2T7	14	26	0	4	3	0	0	-	-	2
OR2C1	12	19	6	4	1	0	0	-	-	2
OR2A12	16	17	5	3	6	0	0	-	-	2
OR2G2	10	24	3	3	1	0	0	-	-	2
OR2A14	21	14	3	2	2	1	0	-	-	2
OR2AJ1	14	15	2	2	0	0	0	-	-	2
OR2C3	19	25	4	4	2	0	0	-	-	2
OR2T8	14	17	2	4	1	1	1	-	-	2
OR2W5	5	13	1	0	1	0	0	-	-	2
OR2F1	25	20	3	0	2	0	1	-	-	2
OR2Z1	15	14	0	2	1	2	0	-	-	2
OR2H2	15	22	2	4	2	1	0	-	-	2
OR2K2	21	23	2	2	3	0	0	-	-	2
OR2A2	25	10	0	1	2	2	0	-	-	2
OR2S2	22	28	1	4	3	1	0	-	-	2
OR2AE1	12	16	1	1	1	2	0	-	-	2
OR2A5	24	24	3	2	3	2	0	-	-	2
OR2B3	18	24	1	4	3	1	0	-	-	2
OR2J2	13	20	2	4	3	1	0	-	-	2
OR2M2	21	16	0	2	0	3	2	-	-	2
OR2A1	25	24	4	3	2	4	0	-	-	2
OR2A7	15	18	1	2	2	3	0	-	-	2
OR2M4	22	13	2	2	1	2	1	-	-	2
OR2W1	16	22	1	4	1	0	0	-	-	2
OR2A25	28	23	1	1	2	4	0	-	-	2
OR2AK2	18	16	2	1	0	0	0	-	-	2
OR2L5	18	20	0	1	3	3	0	-	-	2
OR2W3	15	24	4	3	1	0	0	-	-	2
OR2T10	19	23	2	3	1	1	1	-	-	2
OR2M5	21	14	1	1	2	3	2	-	-	2
OR2L13	18	17	0	1	2	2	0	-	-	2
OR2AT4	16	15	1	1	1	0	0	-	-	2
OR3A3	16	20	0	1	2	2	0	-	-	3
OR3A4	18	17	1	1	1	1	0	-	-	3
OR3A1	16	25	1	1	3	2	0	-	-	3
OR3A2	17	19	1	1	2	2	0	-	-	3
OR4C45	18	14	3	3	0	0	0	-	-	4
OR4B1	20	19	5	0	1	0	0	-	-	4
OR4D5	20	19	6	3	3	0	0	-	-	4
OR4X1	21	12	1	4	0	0	1	-	-	4
OR4X2	18	15	4	3	1	0	0	-	-	4
OR4F6	15	12	5	3	4	0	0	-	-	4
OR4M2	11	16	5	1	2	0	0	-	-	4

OR4N4	14	16	5	2	0	0	1	-	-	4
OR4S1	23	11	4	4	0	0	0	-	-	4
OR4N5	15	21	5	3	2	0	1	-	-	4
OR4D1	16	15	7	4	1	0	0	-	-	4
OR4D6	20	8	4	2	1	0	0	-	-	4
OR4D2	11	16	7	4	1	0	0	-	-	4
OR4K13	19	25	3	2	3	0	0	-	-	4
OR4K14	20	12	6	4	3	0	1	-	-	4
OR4K15	18	19	5	2	3	1	0	-	-	4
OR4K17	22	19	7	3	2	0	0	-	-	4
OR4K1	17	19	6	2	1	0	0	-	-	4
OR4E2	16	30	3	4	0	0	0	-	-	4
OR4K2	21	15	4	1	2	0	0	-	-	4
OR4D10	16	13	6	4	0	1	0	-	-	4
OR4Q3	20	29	5	2	0	0	0	-	-	4
OR4M1	18	17	4	1	2	0	0	-	-	4
OR4N2	16	18	6	2	0	0	1	-	-	4
OR4K5	19	14	7	4	3	0	0	-	-	4
OR4S2	26	24	6	2	0	0	0	-	-	4
OR5K3	15	14	2	0	5	0	0	-	-	5
OR5H14	14	17	2	1	4	3	3	-	-	5
OR5H15	15	15	2	2	2	3	3	-	-	5
OR5H6	11	18	2	2	3	4	3	-	-	5
OR5K2	16	15	1	0	4	0	0	-	-	5
OR5P3	21	32	2	6	2	1	0	-	-	5
OR5M3	16	28	2	6	3	1	0	-	-	5
OR5T1	17	28	9	2	2	1	1	-	-	5
OR5T2	15	22	11	0	2	0	0	-	-	5
OR5M11	22	30	4	4	2	0	0	-	-	5
OR5W2	14	23	11	0	6	1	0	-	-	5
OR5D13	15	14	8	2	1	0	0	-	-	5
OR5D14	19	29	8	3	1	0	0	-	-	5
OR5L2	24	25	3	1	1	0	0	-	-	5
OR5D16	16	27	5	4	2	0	0	-	-	5
OR5D18	20	33	3	3	4	0	0	-	-	5
OR5L1	22	29	5	1	0	0	0	-	-	5
OR5AR1	16	25	8	3	6	0	0	-	-	5
OR5A1	17	33	4	2	2	1	1	-	-	5
OR5M1	22	29	0	2	0	0	0	-	-	5
OR5AP2	19	31	7	2	1	0	0	-	-	5
OR5BF1	12	18	1	1	3	0	1	-	-	5
OR5AX1	10	19	2	1	1	2	1	-	-	5
OR5A2	13	40	3	1	0	0	0	-	-	5
OR5AY1	14	12	3	2	0	1	0	-	-	5
OR5AT1	11	16	4	2	2	0	0	-	-	5
OR5U1	17	17	1	1	0	0	0	-	-	5
OR5V1	15	26	0	1	1	0	0	-	-	5
OR5AK2	15	26	2	0	1	0	0	-	-	5
OR5F1	19	31	6	3	0	0	0	-	-	5
OR5AS1	15	29	5	2	1	2	1	-	-	5
OR5H1	15	16	2	3	3	3	3	-	-	5
OR5I1	22	30	3	2	1	0	0	-	-	5
OR5AC2	13	18	7	1	4	1	0	-	-	5
OR5AN1	18	30	6	0	2	0	0	-	-	5
OR5C1	16	23	7	5	4	1	0	-	-	5
OR5T3	12	27	10	1	3	1	1	-	-	5

OR5AU1	14	18	2	0	1	0	1	-	-	5
OR5BU1	21	14	3	1	1	0	1	-	-	5
OR5H2	15	19	6	2	4	4	3	-	-	5
OR5J2	13	34	5	1	1	0	0	-	-	5
OR6B1	16	24	2	0	1	0	0	-	-	6
OR6Y1	19	26	1	2	1	0	0	-	-	6
OR6T1	14	12	2	0	1	0	0	-	-	6
OR6A2	18	34	4	0	1	0	0	-	-	6
OR6V1	11	18	1	0	1	1	0	-	-	6
OR6Q1	18	32	1	1	0	0	0	-	-	6
OR6P1	12	23	2	0	2	0	0	-	-	6
OR6K2	15	13	1	0	1	0	0	-	-	6
OR6K3	18	13	0	0	0	2	0	-	-	6
OR6K6	19	11	0	2	1	0	0	-	-	6
OR6N1	25	20	0	1	3	0	0	-	-	6
OR6N2	14	17	0	3	2	0	0	-	-	6
OR6M1	13	30	1	0	1	0	0	-	-	6
OR6F1	17	21	2	0	2	0	0	-	-	6
OR6S1	22	17	4	0	2	0	0	-	-	6
OR6B2	14	29	4	0	1	0	0	-	-	6
OR6B3	15	28	4	0	2	0	0	-	-	6
OR6J1	22	22	4	0	1	0	0	-	-	6
OR6X1	12	19	1	2	1	1	0	-	-	6
OR7E24	24	30	2	4	1	2	0	-	-	7
OR7D4	22	33	5	3	2	0	0	-	-	7
OR7G1	22	16	5	3	0	6	0	-	-	7
OR7G2	22	21	1	4	0	5	0	-	-	7
OR8U8	20	30	6	4	1	2	0	-	-	8
OR8H1	22	25	3	1	1	1	0	-	-	8
OR8J1	18	32	4	2	2	0	0	-	-	8
OR8H2	16	25	3	1	0	0	0	-	-	8
OR8I2	14	25	5	2	3	0	0	-	-	8
OR8H3	17	23	3	1	0	1	0	-	-	8
OR8J3	18	24	6	4	2	0	0	-	-	8
OR8K5	17	26	3	3	0	0	0	-	-	8
OR8B4	17	34	4	2	10	0	0	-	-	8
OR8S1	10	28	2	2	0	2	1	-	-	8
OR8U1	17	32	6	6	2	2	0	-	-	8
OR9G9	16	20	2	1	4	0	0	-	-	9
OR9A2	11	11	5	0	0	0	0	-	-	9
OR9Q2	16	23	1	1	1	0	0	-	-	9
OR9G1	12	21	2	1	4	0	0	-	-	9
OR9G4	15	22	7	0	1	0	0	-	-	9
OR9A4	12	8	1	0	0	0	0	-	-	9
OR9I1	21	27	3	1	1	0	0	-	-	9
OR9Q1	15	16	2	1	1	0	0	-	-	9
OR9K2	18	19	6	0	1	0	0	-	-	9
OR10K1	20	21	0	0	1	0	0	-	-	10
OR10S1	19	16	3	2	2	1	1	-	-	10
OR10G4	16	22	1	2	4	5	0	-	-	10
OR10T2	16	25	5	1	2	1	0	-	-	10
OR10AD1	9	19	0	1	0	0	0	-	-	10
OR10K2	19	16	1	0	0	0	0	-	-	10
OR10G6	10	26	1	1	2	1	0	-	-	10
OR10G7	15	23	1	4	5	6	0	-	-	10
OR10G8	15	17	1	1	3	7	0	-	-	10

OR10G9	16	22	0	3	6	5	0	-	-	10
OR10A2	16	23	6	0	3	0	0	-	-	10
OR10A4	14	28	6	2	1	1	0	-	-	10
OR10C1	22	18	4	1	1	0	0	-	-	10
OR10A5	16	30	5	2	3	0	0	-	-	10
OR10P1	21	15	2	0	1	0	0	-	-	10
OR10Q1	14	23	5	1	2	1	0	-	-	10
OR10X1	7	19	0	1	2	0	0	-	-	10
OR10Z1	9	22	1	2	2	0	0	-	-	10
OR10H4	14	10	0	3	3	1	0	-	-	10
OR10J3	15	18	2	1	7	0	0	-	-	10
OR10J1	17	23	6	0	3	0	1	-	-	10
OR10H2	17	19	1	4	5	0	0	-	-	10
OR10H3	9	11	0	4	3	1	0	-	-	10
OR10A6	12	17	1	3	1	4	0	-	-	10
OR10A3	15	19	3	3	3	1	0	-	-	10
OR10W1	13	16	3	2	1	0	0	-	-	10
OR10V1	17	11	3	2	1	0	0	-	-	10
OR10A7	21	23	4	2	0	0	0	-	-	10
OR10J5	21	15	4	0	3	0	0	-	-	10
OR10G3	18	14	1	3	3	0	0	-	-	10
OR10G2	22	25	2	2	3	0	0	-	-	10
OR10AG1	21	26	2	2	0	0	0	-	-	10
OR11A1	18	14	0	1	0	0	0	-	-	11
OR11H4	20	26	1	0	2	1	0	-	-	11
OR11G2	18	25	3	0	2	2	1	-	-	11
OR11H6	24	21	2	2	5	0	0	-	-	11
OR11L1	14	26	1	1	4	1	0	-	-	11
OR12D3	25	13	2	0	0	0	1	-	-	12
OR12D2	16	20	1	1	1	0	0	-	-	12
OR13H1	13	13	0	0	2	0	0	-	-	13
OR13G1	12	18	2	0	0	2	0	-	-	13
OR13A1	19	23	0	2	5	1	0	-	-	13
OR13D1	21	34	1	1	3	1	0	-	-	13
OR13C2	20	26	3	1	3	0	0	-	-	13
OR13C3	23	30	0	4	4	0	0	-	-	13
OR13J1	12	23	2	2	2	0	0	-	-	13
OR13C5	19	21	3	0	3	0	0	-	-	13
OR13C4	23	31	0	3	5	0	0	-	-	13
OR13C8	23	19	2	0	5	0	0	-	-	13
OR13C9	21	30	2	1	3	0	0	-	-	13
OR13F1	18	24	3	1	3	0	0	-	-	13
OR51V1	17	6	8	3	1	0	0	-	-	51
OR51J1	5	6	5	2	2	0	0	-	-	51
OR51L1	14	5	7	3	1	0	0	-	-	51
OR51M1	9	2	5	3	1	1	0	-	-	51
OR51Q1	3	7	5	3	4	0	0	-	-	51
OR51F1	12	8	3	3	4	0	0	-	-	51
OR51T1	15	7	4	2	3	0	0	-	-	51
OR51A7	11	7	8	3	2	1	0	-	-	51
OR51B2	15	4	1	4	0	0	0	-	-	51
OR51G2	14	9	8	3	4	0	0	-	-	51
OR51S1	6	2	4	0	0	0	0	-	-	51
OR51F2	16	9	5	0	3	0	0	-	-	51
OR51D1	10	6	9	3	0	1	0	-	-	51
OR51I1	12	6	7	3	3	0	0	-	-	51

OR52E2	14	13	11	3	5	0	0	-	-	52
OR52L1	8	9	7	3	1	0	0	-	-	52
OR52J3	12	11	11	4	2	0	0	-	-	52
OR52N2	8	4	5	4	6	1	0	-	-	52
OR52E6	17	7	11	2	4	2	0	-	-	52
OR52R1	9	9	9	2	1	1	0	-	-	52
OR52I1	18	4	2	0	3	0	0	-	-	52
OR52I2	18	5	1	0	3	0	0	-	-	52
OR52M1	11	7	5	3	4	0	0	-	-	52
OR52W1	13	6	8	2	3	0	0	-	-	52
OR52B4	10	8	4	2	4	0	0	-	-	52
OR52A1	11	5	10	4	3	1	0	-	-	52
OR52B6	18	8	6	1	5	0	0	-	-	52
OR52D1	16	13	11	3	1	1	0	-	-	52
OR52E8	20	9	16	4	6	1	0	-	-	52
OR52A4	7	3	4	2	0	0	0	-	-	52
OR56B4	11	2	2	4	1	1	0	-	-	56
