# Supplementary material for: Common peptides shed light on evolution of Olfactory Receptors
Source: BMC Evol Biol. 2009 May 5;9:91. doi: 10.1186/1471-2148-9-91 (PMC2681464; doi:10.1186/1471-2148-9-91)
Supplement: Additional file 10 — Pufferfish ORs CP numbers and cluster assignment. Number of CPs from each ancestor occurring in each pufferfish OR and cluster assignment for each pufferfish OR. [file 1471-2148-9-91-S10.pdf]

Name	Number of A1 CPs	Number of A8 novel CPs	Number of pufferfish novel CPs	Cluster number A1 novel CPs
Fr3OR117.2	9	0	1	1
Fr3OR2346.1	10	0	0	1
Fr3OR2346.3	17	2	4	1
Fr3OR2346.4	12	1	0	1
Fr3OR3630.1	21	0	1	1
Fr3OR4429.2	20	0	1	1
Fr3OR7149.1	10	0	0	1
Fr3OR3905.1	8	1	1	2
AB031380	11	1	6	3
AB031383	10	1	6	3
AB031384	7	1	6	3
Fr3OR4479.2	10	1	7	3
Fr3OR6269.1	8	1	6	3
Fr3OR119.1	10	1	0	4
Fr3OR142.7	9	0	0	4
Fr3OR4429.1	7	0	0	5
Fr3OR5287.2	16	0	0	5
Fr3OR117.1	19	2	0	6
Fr3OR123.1	18	3	2	6
Fr3OR2346.2	15	3	2	6
Fr3OR2346.7	18	1	6	6
Fr3OR3763.1	17	1	5	6
Fr3OR3763.2	17	2	8	6
Fr3OR4133.2	20	2	7	6
Fr3OR6765.1	18	1	5	6
Fr3OR142.1	10	0	0	7
Fr3OR6818.1	5	1	1	7
Fr3OR7011.1	7	1	0	7
AB031385	14	2	1	8
Fr3OR123.2	13	0	0	8
Fr3OR5510.2	19	0	1	8
Fr3OR5511.2	18	0	0	8
Fr3OR142.3	12	0	2	9
Fr3OR142.6	9	0	0	9
Fr3OR1026.2	16	1	2	10
Fr3OR2346.5	9	0	0	10
Fr3OR4563.4	12	0	2	10
Fr3OR4563.5	14	1	1	10
Fr3OR8298.1	17	1	1	10
Fr3OR18.1	3	0	0	-
Fr3OR217.1	4	1	1	-
Fr3OR59.1	1	0	1	-
Fr3OR6030.1	1	0	0	-
Fr3OR633.1	0	0	1	-
