# Supplementary material for: Common peptides shed light on evolution of Olfactory Receptors
Source: BMC Evol Biol. 2009 May 5;9:91. doi: 10.1186/1471-2148-9-91 (PMC2681464; doi:10.1186/1471-2148-9-91)
Supplement: Additional file 11 — Lizard ORs CP numbers and cluster assignment. Number of CPs from each ancestor occurring in each Lizard OR and cluster assignment for each Lizard OR. [file 1471-2148-9-91-S11.pdf]

Name	Number of A1 CPs	Number of A2 novel CPs	Number of A3 novel CPs	Number of A7 novel CPs	Number of lizard novel CPs	Cluster number lizard novel CPs
ORcand.24	11	29	3	0	2	1
ORcand.53	11	29	3	0	2	1
ORcand.13	19	20	5	0	3	2
ORcand.16	12	19	1	0	2	2
ORcand.22	20	17	2	0	2	2
ORcand.9	20	21	2	0	2	2
ORcand.128	17	31	1	0	2	3
ORcand.3	16	26	5	0	3	3
ORcand.42	17	26	5	0	3	3
ORcand.71	16	26	2	0	2	3
ORcand.30	13	11	2	0	2	4
ORcand.48	7	13	2	0	2	4
ORcand.49	15	9	3	0	2	4
ORcand.70	14	8	3	0	2	4
ORcand.78	12	11	2	0	3	4
ORcand.101	14	19	3	0	2	5
ORcand.102	20	12	4	0	4	5
ORcand.104	21	11	6	0	5	5
ORcand.106	20	10	1	1	7	5
ORcand.110	17	10	2	0	4	5
ORcand.117	15	17	2	1	2	5
ORcand.12	12	11	2	0	11	5
ORcand.120	10	11	2	1	4	5
ORcand.122	20	22	2	0	2	5
ORcand.132	20	23	8	0	2	5
ORcand.133	14	9	4	1	7	5
ORcand.28	13	4	3	0	6	5
ORcand.31	13	11	2	0	11	5
ORcand.32	12	10	2	0	10	5
ORcand.39	13	13	2	0	11	5
ORcand.44	18	11	4	1	6	5
ORcand.47	13	14	4	0	6	5
ORcand.5	13	7	3	0	7	5
ORcand.52	9	10	8	1	4	5
ORcand.59	13	12	2	0	11	5
ORcand.61	13	10	4	0	6	5
ORcand.89	19	7	4	0	5	5
ORcand.96	19	14	5	0	6	5
ORcand.107	16	14	3	1	2	6
ORcand.90	18	26	1	0	2	6
ORcand.134	12	27	1	0	2	7
ORcand.138	13	25	3	0	3	7
ORcand.139	19	24	2	0	2	7
ORcand.100	13	7	5	0	1	-
ORcand.103	23	26	12	0	0	-
ORcand.11	23	23	2	0	1	-
ORcand.111	12	20	2	0	1	-
ORcand.114	18	17	0	0	0	-
ORcand.118	15	18	2	0	0	-
ORcand.119	18	31	4	0	1	-
ORcand.121	21	28	2	0	1	-
ORcand.123	19	26	3	0	2	-

ORcand.124	19	8	2	0	0	-
ORcand.125	13	26	3	0	0	-
ORcand.126	19	18	2	0	1	-
ORcand.131	13	17	0	0	1	-
ORcand.135	13	29	1	0	0	-
ORcand.136	23	25	8	0	2	-
ORcand.137	15	16	5	1	1	-
ORcand.140	20	34	1	0	0	-
ORcand.141	17	18	2	0	1	-
ORcand.142	19	18	0	0	1	-
ORcand.143	19	18	2	1	0	-
ORcand.144	26	21	1	0	0	-
ORcand.145	17	25	6	0	0	-
ORcand.146	24	17	0	0	0	-
ORcand.149	26	26	2	0	0	-
ORcand.150	24	20	3	0	1	-
ORcand.151	18	13	2	0	3	-
ORcand.154	15	24	1	0	3	-
ORcand.155	17	27	0	0	2	-
ORcand.156	11	16	1	0	1	-
ORcand.157	24	24	6	0	0	-
ORcand.158	13	26	3	0	0	-
ORcand.17	13	22	5	0	0	-
ORcand.20	11	15	1	0	0	-
ORcand.21	22	23	2	0	1	-
ORcand.25	13	35	4	0	0	-
ORcand.27	0	0	0	0	0	-
ORcand.29	21	35	0	0	0	-
ORcand.33	15	28	3	1	1	-
ORcand.34	21	26	1	0	0	-
ORcand.35	12	14	3	0	2	-
ORcand.36	19	35	9	0	3	-
ORcand.37	19	17	0	0	1	-
ORcand.38	16	29	2	0	1	-
ORcand.40	14	28	2	0	1	-
ORcand.41	17	18	6	0	1	-
ORcand.43	17	26	0	0	0	-
ORcand.45	19	28	3	0	0	-
ORcand.46	4	3	0	0	1	-
ORcand.50	21	23	3	0	0	-
ORcand.54	25	29	8	0	1	-
ORcand.55	18	29	7	0	1	-
ORcand.58	13	11	6	0	1	-
ORcand.6	24	27	1	0	1	-
ORcand.60	16	22	6	0	0	-
ORcand.62	20	30	7	0	1	-
ORcand.63	21	28	3	0	1	-
ORcand.64	22	16	3	0	2	-
ORcand.65	23	14	0	0	0	-
ORcand.66	13	22	5	0	0	-
ORcand.67	16	9	6	0	1	-
ORcand.69	21	8	3	0	1	-
ORcand.7	6	7	1	1	0	-
ORcand.73	20	19	3	0	1	-
ORcand.74	22	12	5	0	0	-
ORcand.75	25	18	1	0	0	-

ORcand.76	19	34	1	0	0	-
ORcand.77	24	18	9	0	0	-
ORcand.79	16	11	1	0	1	-
ORcand.8	20	28	0	0	1	-
ORcand.80	18	16	4	0	1	-
ORcand.81	10	23	4	0	1	-
ORcand.83	18	34	6	0	1	-
ORcand.84	18	18	4	0	1	-
ORcand.85	23	24	5	0	1	-
ORcand.86	18	26	2	0	0	-
ORcand.91	14	21	3	0	0	-
ORcand.98	19	16	3	0	2	-
