# Supplementary material for: Common peptides shed light on evolution of Olfactory Receptors
Source: BMC Evol Biol. 2009 May 5;9:91. doi: 10.1186/1471-2148-9-91 (PMC2681464; doi:10.1186/1471-2148-9-91)
Supplement: Additional file 12 — Platypus ORs CP numbers and cluster assignment. Number of CPs from each ancestor occurring in each Platypus OR and cluster assignment for each Platypus OR. [file 1471-2148-9-91-S12.pdf]

Legend

A	Number of A1 CPs
B	Number of A2 novel CPs
C	Number of A3 novel CPs
D	Number of A4 novel CPs
E	Number of platypus novel CPs
F	Cluster number A3 novel CPs
G	Cluster number A4 novel CPs

Name	A	B	C	D	E	F	G
Plat_OR599	25	18	5	8	0	1	3
Plat_OR609	23	18	7	11	0	1	3
Plat_OR610	28	20	6	6	0	1	3
Plat_OR611	22	18	7	12	0	1	3
Plat_OR613	21	21	5	5	0	1	3
Plat_OR616	24	16	8	7	0	1	3
Plat_OR593	25	25	9	4	0	1	-
Plat_OR136	20	20	5	6	4	2	6
Plat_OR357	19	16	7	5	3	2	6
Plat_OR476	24	21	5	6	5	2	6
Plat_OR477	20	17	5	2	6	2	6
Plat_OR371	24	21	5	3	0	2	-
Plat_OR396	15	33	5	2	0	2	-
Plat_OR406	22	20	7	3	5	2	-
Plat_OR475	27	21	7	6	3	2	-
Plat_OR493	25	22	6	3	3	2	-
Plat_OR542	11	8	5	4	1	2	-
Plat_OR553	22	18	5	4	6	2	-
Plat_OR556	17	13	6	4	7	2	-
Plat_OR583	24	23	7	2	4	2	-
Plat_OR712	19	23	6	3	4	2	-
Plat_OR713	22	25	5	2	5	2	-
Plat_OR714	25	25	6	3	5	2	-
Plat_OR715	20	28	6	3	2	2	-
Plat_OR244	17	24	7	2	0	3	-
Plat_OR479	22	29	8	3	0	3	-
Plat_OR689	15	43	6	4	0	3	-
Plat_OR697	20	38	8	0	0	3	-
Plat_OR728	21	43	6	1	0	3	-
Plat_OR729	21	44	6	2	0	3	-
Plat_OR738	15	26	6	2	0	3	-
Plat_OR739	20	29	6	2	1	3	-
Plat_OR744	19	29	7	1	0	3	-
Plat_OR745	20	38	5	1	1	3	-
Plat_OR746	22	35	6	3	0	3	-
Plat_OR334	13	7	8	5	0	4	1
Plat_OR202	10	7	6	8	1	4	2
Plat_OR39	16	8	11	9	0	4	6
Plat_OR403	19	7	13	6	0	4	6
Plat_OR344	11	7	8	4	0	4	-
Plat_OR379	12	2	5	5	0	4	-
Plat_OR430	17	5	9	5	0	4	-

Plat_OR687	27	26	6	11	0	5	3
Plat_OR348	23	25	5	2	0	5	-
Plat_OR349	22	28	5	2	0	5	-
Plat_OR199	17	18	9	8	0	6	3
Plat_OR205	20	15	8	6	1	6	3
Plat_OR378	19	20	9	6	1	6	3
Plat_OR424	20	15	6	7	1	6	3
Plat_OR500	19	11	7	9	1	6	3
Plat_OR520	18	13	9	10	0	6	3
Plat_OR162	19	17	7	3	0	6	-
Plat_OR591	21	23	6	1	0	6	-
Plat_OR702	17	15	6	4	1	6	-
Plat_OR230	22	27	6	6	1	7	1
Plat_OR531	25	36	5	9	0	7	1
Plat_OR517	17	21	6	6	0	7	5
Plat_OR337	14	24	6	7	0	7	-
Plat_OR91	25	26	5	3	4	7	-
Plat_OR596	19	20	8	5	0	8	3
Plat_OR600	27	23	7	9	0	8	3
Plat_OR618	25	15	8	8	0	8	3
Plat_OR594	19	25	6	3	0	8	-
Plat_OR606	23	14	5	4	0	8	-
Plat_OR659	22	35	8	7	0	9	-
Plat_OR719	19	27	6	4	0	9	-
Plat_OR725	12	38	11	3	0	9	-
Plat_OR726	16	35	11	2	0	9	-
Plat_OR742	16	26	5	3	0	9	-
Plat_OR204	16	17	5	5	7	10	6
Plat_OR289	22	20	6	8	5	10	6
Plat_OR374	20	20	6	8	6	10	6
Plat_OR397	21	20	5	8	6	10	6
Plat_OR446	14	17	5	5	7	10	6
Plat_OR101	25	23	5	2	4	10	-
Plat_OR181	14	17	6	2	11	10	-
Plat_OR227	15	15	6	2	10	10	-
Plat_OR251	15	17	6	2	10	10	-
Plat_OR298	16	15	6	2	11	10	-
Plat_OR317	20	21	5	3	0	10	-
Plat_OR459	17	14	6	2	8	10	-
Plat_OR214	9	11	7	8	0	11	1
Plat_OR196	17	16	11	5	0	11	5
Plat_OR208	12	6	6	6	0	11	5
Plat_OR325	10	8	5	5	0	11	5
Plat_OR342	12	9	10	8	0	11	5
Plat_OR389	15	14	8	5	0	11	5
Plat_OR193	11	9	8	2	0	11	-
Plat_OR250	17	11	12	4	0	11	-
Plat_OR254	16	17	13	2	0	11	-
Plat_OR528	14	6	7	4	0	11	-
Plat_OR305	10	22	5	2	2	12	-
Plat_OR335	11	23	6	2	2	12	-
Plat_OR639	21	25	6	2	2	12	-

Plat_OR734	22	26	6	4	2	12	-
Plat_OR215	26	29	1	6	2	-	1
Plat_OR328	25	33	0	10	2	-	1
Plat_OR355	23	31	1	5	2	-	1
Plat_OR414	20	34	1	10	1	-	1
Plat_OR435	22	36	1	6	1	-	1
Plat_OR484	17	36	0	6	3	-	1
Plat_OR50	25	34	1	7	3	-	1
Plat_OR80	20	26	0	7	1	-	1
Plat_OR245	14	3	4	8	5	-	2
Plat_OR422	14	4	3	8	4	-	2
Plat_OR423	13	5	3	6	0	-	2
Plat_OR525	15	4	4	8	3	-	2
Plat_OR540	13	4	3	5	4	-	2
Plat_OR548	13	4	4	9	4	-	2
Plat_OR67	13	3	3	9	0	-	2
Plat_OR213	19	11	2	7	0	-	3
Plat_OR224	18	8	4	6	1	-	3
Plat_OR598	28	22	3	10	0	-	3
Plat_OR602	18	17	4	6	0	-	3
Plat_OR607	24	14	3	7	1	-	3
Plat_OR608	20	27	4	7	0	-	3
Plat_OR614	22	12	6	6	0	-	3
Plat_OR749	25	29	2	8	0	-	3
Plat_OR751	24	21	4	8	0	-	3
Plat_OR764	25	22	4	7	0	-	3
Plat_OR257	25	24	1	11	0	-	4
Plat_OR288	19	22	2	17	0	-	4
Plat_OR297	16	16	3	16	0	-	4
Plat_OR322	19	26	2	10	0	-	4
Plat_OR326	16	20	3	8	0	-	4
Plat_OR426	20	22	2	7	0	-	4
Plat_OR427	25	25	3	6	0	-	4
Plat_OR545	13	24	4	9	0	-	4
Plat_OR552	17	23	4	11	0	-	4
Plat_OR58	24	23	1	11	0	-	4
Plat_OR158	10	2	4	6	0	-	5
Plat_OR388	14	4	5	7	0	-	5
Plat_OR437	6	17	3	10	1	-	5
Plat_OR490	17	22	3	7	0	-	5
Plat_OR568	17	30	0	8	0	-	5
Plat_OR681	17	30	4	7	0	-	5
Plat_OR691	15	22	4	7	0	-	5
Plat_OR692	15	26	2	5	0	-	5
Plat_OR151	31	28	3	5	8	-	6
Plat_OR165	20	18	2	7	4	-	6
Plat_OR562	13	13	4	5	4	-	6
Plat_OR118	17	25	3	1	0	-	-
Plat_OR123	19	26	3	2	0	-	-
Plat_OR131	22	18	4	2	0	-	-
Plat_OR145	16	14	4	8	0	-	-
Plat_OR152	21	15	1	0	0	-	-

Plat_OR157	30	20	4	3	9	-	-
Plat_OR167	17	17	1	0	0	-	-
Plat_OR168	17	20	0	3	0	-	-
Plat_OR184	23	27	2	2	2	-	-
Plat_OR189	18	20	4	3	0	-	-
Plat_OR190	13	13	4	1	2	-	-
Plat_OR201	17	14	4	6	0	-	-
Plat_OR212	22	26	3	2	0	-	-
Plat_OR233	17	29	2	3	1	-	-
Plat_OR238	20	20	3	4	0	-	-
Plat_OR260	19	27	4	3	5	-	-
Plat_OR262	25	19	4	4	5	-	-
Plat_OR271	15	25	3	1	1	-	-
Plat_OR278	13	17	4	3	4	-	-
Plat_OR281	30	22	4	3	7	-	-
Plat_OR282	10	23	4	1	2	-	-
Plat_OR290	15	22	4	3	6	-	-
Plat_OR30	16	9	3	0	1	-	-
Plat_OR308	17	22	3	1	0	-	-
Plat_OR314	18	22	3	3	6	-	-
Plat_OR330	19	19	2	4	8	-	-
Plat_OR336	18	18	3	1	0	-	-
Plat_OR34	18	21	2	3	5	-	-
Plat_OR347	12	23	5	3	0	-	-
Plat_OR35	20	17	4	1	0	-	-
Plat_OR363	10	19	4	2	0	-	-
Plat_OR369	18	25	3	3	0	-	-
Plat_OR382	22	12	1	0	0	-	-
Plat_OR383	15	22	4	4	0	-	-
Plat_OR399	15	23	3	4	0	-	-
Plat_OR405	18	25	4	2	0	-	-
Plat_OR44	30	20	4	3	10	-	-
Plat_OR460	11	16	0	2	0	-	-
Plat_OR488	15	17	2	1	0	-	-
Plat_OR491	24	23	3	4	10	-	-
Plat_OR510	20	16	4	4	8	-	-
Plat_OR519	8	2	1	2	0	-	-
Plat_OR527	20	11	4	0	0	-	-
Plat_OR536	25	18	2	1	0	-	-
Plat_OR539	13	12	0	0	2	-	-
Plat_OR558	13	18	1	2	0	-	-
Plat_OR560	15	18	2	3	0	-	-
Plat_OR563	14	12	0	3	0	-	-
Plat_OR566	21	18	1	3	0	-	-
Plat_OR570	16	31	2	3	0	-	-
Plat_OR571	13	17	2	3	0	-	-
Plat_OR573	16	14	1	2	0	-	-
Plat_OR575	18	31	2	3	0	-	-
Plat_OR578	15	23	2	0	0	-	-
Plat_OR579	16	31	1	2	0	-	-
Plat_OR584	26	15	4	2	2	-	-
Plat_OR585	27	18	4	2	4	-	-

Plat_OR590	15	21	3	3	0	-	-
Plat_OR601	27	18	4	2	0	-	-
Plat_OR612	20	20	3	3	0	-	-
Plat_OR619	17	30	2	4	3	-	-
Plat_OR621	17	28	3	3	4	-	-
Plat_OR623	19	29	4	3	3	-	-
Plat_OR624	20	24	1	2	4	-	-
Plat_OR626	20	27	2	3	2	-	-
Plat_OR630	19	31	2	3	2	-	-
Plat_OR631	29	29	2	0	0	-	-
Plat_OR633	27	28	2	0	0	-	-
Plat_OR636	11	29	0	1	1	-	-
Plat_OR638	16	29	2	1	1	-	-
Plat_OR641	21	25	3	2	1	-	-
Plat_OR642	25	29	3	0	1	-	-
Plat_OR643	24	32	0	2	0	-	-
Plat_OR646	24	27	1	2	2	-	-
Plat_OR647	27	26	1	2	2	-	-
Plat_OR648	28	23	1	3	2	-	-
Plat_OR649	27	25	1	3	1	-	-
Plat_OR65	10	3	4	3	5	-	-
Plat_OR650	17	40	4	2	2	-	-
Plat_OR651	17	37	4	2	2	-	-
Plat_OR652	18	36	4	3	2	-	-
Plat_OR653	22	29	1	1	1	-	-
Plat_OR657	23	27	2	5	0	-	-
Plat_OR690	13	29	3	5	0	-	-
Plat_OR698	17	25	4	3	0	-	-
Plat_OR699	16	14	4	2	0	-	-
Plat_OR70	14	27	4	4	0	-	-
Plat_OR700	16	14	4	4	0	-	-
Plat_OR704	14	11	1	4	0	-	-
Plat_OR709	21	24	4	2	0	-	-
Plat_OR711	24	20	4	2	3	-	-
Plat_OR716	24	29	3	1	0	-	-
Plat_OR718	19	24	4	5	0	-	-
Plat_OR720	27	27	3	1	0	-	-
Plat_OR721	26	31	3	3	0	-	-
Plat_OR722	18	28	4	2	0	-	-
Plat_OR724	14	27	4	4	0	-	-
Plat_OR727	19	23	2	0	0	-	-
Plat_OR731	13	25	4	3	0	-	-
Plat_OR735	17	23	0	3	0	-	-
Plat_OR740	18	29	4	2	0	-	-
Plat_OR743	17	30	1	2	0	-	-
Plat_OR747	18	29	2	5	0	-	-
Plat_OR748	17	31	3	4	0	-	-
Plat_OR753	14	22	4	2	0	-	-
Plat_OR87	23	14	1	1	0	-	-
Plat_OR94	18	18	3	1	0	-	-
Plat_OR96	18	27	4	3	5	-	-
Plat_OR99	19	20	3	3	4	-	-
